# Synthesis of 6-Alkynylated Purine-Containing DNA via On-Column Sonogashira Coupling and Investigation of Their Base-Pairing Properties

**DOI:** 10.3390/molecules28041766

**Published:** 2023-02-13

**Authors:** Hidenori Okamura, Giang Hoang Trinh, Zhuoxin Dong, Wenjue Fan, Fumi Nagatsugi

**Affiliations:** 1 Institute of Multidisciplinary Research for Advanced Materials, Tohoku University, 2-1-1 Katahira, Aoba-ku, Sendai 980-8577, Miyagi, Japan; 2 Department of Chemistry, Graduate School of Science, Tohoku University, 6-3 Aramaki Aza-Aoba, Aoba-ku, Sendai 980-8577, Miyagi, Japan

**Keywords:** unnatural base pair, DNA, solid-phase synthesis, Sonogashira coupling, modified nucleosides, DNA major groove, hydrogen bonds

## Abstract

Synthetic unnatural base pairs have been proven to be attractive tools for the development of DNA-based biotechnology. Our group has very recently reported on alkynylated purine–pyridazine pairs, which exhibit selective and stable base-pairing via hydrogen bond formation between pseudo-nucleobases in the major groove of duplex DNA. In this study, we attempted to develop an on-column synthesis methodology of oligodeoxynucleotides (ODNs) containing alkynylated purine derivatives to systematically explore the relationship between the structure and the corresponding base-pairing ability. Through Sonogashira coupling of the ethynyl pseudo-nucleobases and CPG-bound ODNs containing 6-iodopurine, we have demonstrated the synthesis of the ODNs containing three ^N^Pu derivatives (^N^Pu1, ^N^Pu2, ^N^Pu3) as well as three ^O^Pu derivatives (^O^Pu1, ^O^Pu2, ^O^Pu3). The base-pairing properties of each alkynylated purine derivative revealed that the structures of pseudo-nucleobases influence the base pair stability and selectivity. Notably, we found that ^O^Pu1 bearing 2-pyrimidinone exhibits higher stability to the complementary ^N^Pz than the original ^O^Pu, thereby demonstrating the potential of the on-column strategy for convenient screening of the alkynylated purine derivatives with superior pairing ability.

## 1. Introduction

DNA is an essential biopolymer that plays a pivotal role in gene expression through replication and transferring genetic information. The functional basis of DNA lies in the formation of base pairs between A and T as well as G and C through the complementary hydrogen bonds, respectively. The selectivity of the base-pairing process, in combination with the facile programmability due to the countless permutations of base pair sequences, have made DNA an attractive platform for numerous applications in biotechnology [1,2] and nanotechnology [3,4,5], as well as drug discovery studies [6,7]. DNA-based technologies have immense potential, and efforts to harness them in a wide range of applications have resulted in the development of unnatural base pairs (UBPs) being extensively studied for the past several decades [8,9,10,11].

The UBPs are sets of synthetic nucleosides that exhibit specificity to each other for base-pairing and DNA polymerase-mediated strand replication. The design concept of UBP was initially proposed by Rich in 1962 [12] when he discussed the possibility of synthetic isoC-isoG as a third base pair. Since then, several research groups have reported the creation of UBPs. The conventional UBPs are generally classified into three types: hydrogen-bonding base pairs [13,14,15,16,17,18], hydrophobic base pairs [19,20,21], and metal-mediated base pairs [22,23,24]. UBPs are useful for acquiring highly functional DNA aptamers by infusing chemical and structural diversity in the DNA strands [25,26]. They have also been applied for site-specific labeling of nucleic acids [27], detection of target nucleic acid sequences [28], as well as the incorporation of non-natural amino acids into proteins in vitro [29] and in semisynthetic organisms [30].

**Figure 1:**
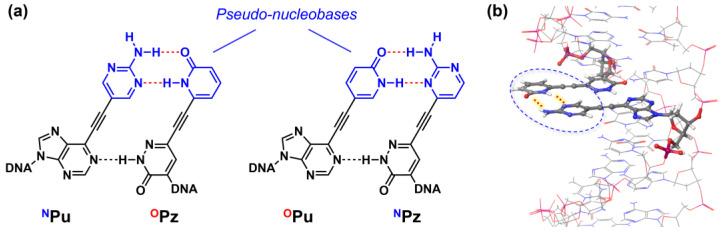
(**a**) Structures of the previously reported alkynylated purine–pyridazine base pairs. (**b**) A model structure of the duplex DNA incorporating the ^N^Pu–^O^Pz pair. The hydrogen bonding between the pseudo-nucleobases is highlighted.

In our effort to expand the design repertoire of UBP, we have recently reported a new type of UBP, which include the base pairs ^N^Pu–^O^Pz and ^O^Pu–^N^Pz, consisting of alkynylated purine and pyridazine as base surrogates [31]. Our unnatural nucleobases are composed of three functional units: (1) purine and pyridazine core structures for maintaining the stacking interaction within the double-helix, (2) nucleobase-like heteroaromatics (“pseudo-nucleobases”) as recognition units, and (3) alkyne spacers that dislocate the pseudo-nucleobases from the Watson–Crick interface (Figure 1a). In a case where 2-aminopyirimidine and 2-pyridone were adopted as the pseudo-nucleobases, these unnatural bases exhibit stable and selective base-pairing in duplex DNAs only when the combinations of the pseudo-nucleobases allow appropriate inter-base hydrogen bonding (Figure 1b).

While assessing the base-pairing properties of our alkynylated purine–pyridazine base pairs, we noticed that ^N^Pu–^O^Pz and ^O^Pu–^N^Pz exhibited somewhat different base-pairing stability. This suggested that the thermal stability of the alkynylated purine–pyridazine base pairs might depend on the structure of pseudo-nucleobases and that mechanistic studies may facilitate efforts to improve the base-pairing properties; however, one-by-one preparation of the corresponding phosphoramidites for solid-phase DNA synthesis imposes significant inefficiency in terms of investigating various alkynylated nucleosides. We therefore sought to explore the post-synthetic construction of the alkynylated nucleoside derivatives on DNA to facilitate the systematic and efficient preparation of the oligodeoxynucleotides (ODNs) containing the UBPs being studied. The post-synthesis modification of nucleic acids is a chemical approach in which the nucleoside analogs bearing reactive handles are first incorporated into ODNs and subsequently converted into the desired nucleoside analogs via conjugation chemistry. Various conversion chemistries, such as substitution reactions [32,33,34,35,36,37,38], click reactions [39,40], amide bond formation [41], oxime or hydrazone formation [42], as well as metal-catalyzed cross-coupling reactions [43,44,45,46,47,48,49,50,51,52,53,54,55], have been employed for the post-synthesis modification of canonical nucleosides. In addition, several unnatural nucleoside analogs have also been synthesized via the post-synthesis approach [56,57,58,59]. Hence, we circumvented the complicated and time-consuming synthesis of the modified phosphoramidites by using a post-synthesis approach to investigate the relation between the structure and the base-pairing properties of alkynylated nucleobases.

**Figure 2:**
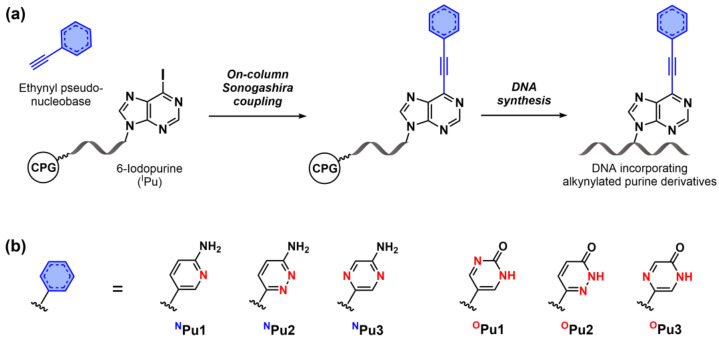
(**a**) On-column synthesis of ODNs incorporating alkynylated purine derivatives via Sonogashira coupling reactions; CPG: controlled pore glass. (**b**) Structures of the purine derivatives synthesized in this study.

Herein, we describe the post-synthesis approach that allows the preparation of ODNs containing various alkynylated purine derivatives (Figure 2a). Specifically, we demonstrated the synthesis of ODNs containing three ^N^Pu derivatives (^N^Pu1, ^N^Pu2, ^N^Pu3) as well as three ^O^Pu derivatives by subjecting the controlled pore glass (CPG)-bound ODNs containing 6-iodopurine (^I^Pu) to Sonogashira coupling (^O^Pu1, ^O^Pu2, ^O^Pu3) (Figure 2b). The base-pairing properties of each alkynylated purine derivative were investigated by UV melting temperature measurements, revealing that the structures of the pseudo-nucleobases influence the base pair stability and selectivity. Notably, we found that ^O^Pu1 has superior base-pairing properties as compared to the original ^O^Pu, which demonstrates the potential of the on-column strategy for the facile screening of alkynylated purine derivatives with optimal pairing ability.

## 2. Results

### 2.1. On-Column Synthesis of Oligodeoxynucleotides Containing the Alkynylated Purine Derivatives

**Scheme 1:**
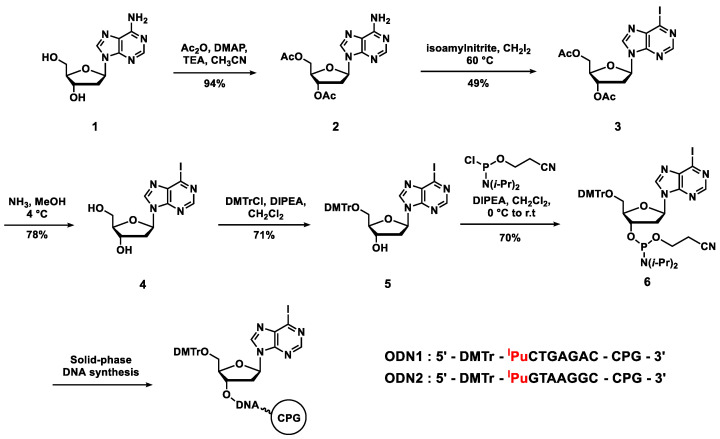
Synthesis of the CPG-bound ODNs containing ^I^Pu at their 5′ terminal.

To demonstrate the feasibility of the on-column synthesis strategy, we planned to synthesize 15-mer ODNs containing ^O^Pu and ^N^Pu derivatives in the middle of the sequences by on-column Sonogashira coupling followed by a solid-phase elongation of the remaining part of the ODNs. In a previous study, we showed that the phosphoramidite building blocks of ^O^Pu and ^N^Pu can be synthesized by coupling ^I^Pu deoxyriboside with ethynyl pseudo-nucleobases by Sonogashira coupling [31]. Thus, considering that CPG-bound ODNs are compatible with the reaction conditions of Sonogashira coupling, we reasoned that the post-synthesis approaches using ^I^Pu-containing ODN can be employed for the on-DNA synthesis of the alkynylated purine derivatives. To synthesize the ODNs containing ^I^Pu at its 5′ terminal, the phosphoramidite of ^I^Pu was synthesized as shown in Scheme 1. The 2′-deoxyadenosine **1** was acetylated and subsequently iodinated to afford protected 6-iodopurine 2′-deoxyriboside **3**. The acetyl-protected ^I^Pu **3** was then treated with methanolic ammonia to provide fully deprotected ^I^Pu deoxyriboside **4**. Finally, protection of the 5′-OH group with the 4,4′-dimethoxytrityl (DMTr) moiety, followed by phosphitylation at the 3′-OH group, afforded the phosphoramidite of ^I^Pu **6**. The phosphoramidite **6** was then incorporated into ODN1 and ODN2 using an automated DNA synthesizer in a DMT-ON mode.

**Scheme 2:**
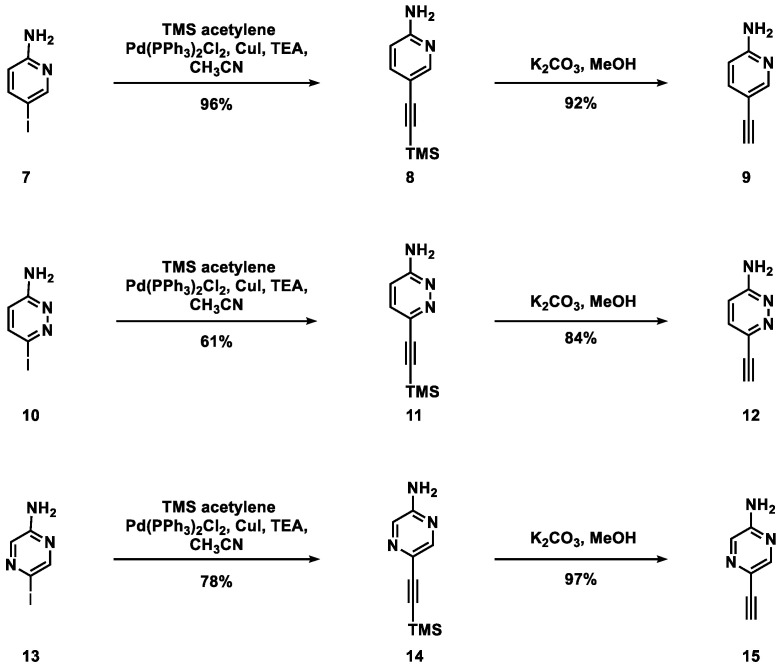
Synthesis of the ethynyl pseudo-nucleobases for ^N^Pu derivatives.

**Scheme 3:**
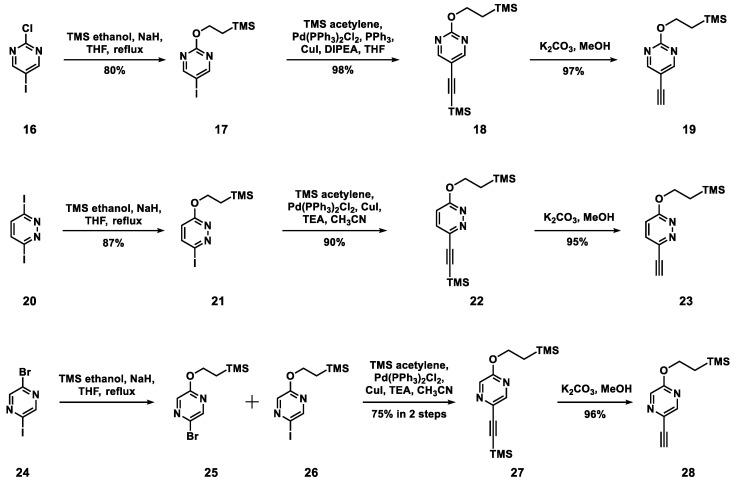
Synthesis of the ethynyl pseudo-nucleobases for ^O^Pu derivatives.

At the same time, the corresponding ethynyl pseudo-nucleobases were prepared according to Scheme 2 and Scheme 3. The pseudo-nucleobase units for ^N^Pu derivatives were synthesized by coupling halogenated aminoheteroaromatics with trimethylsilylacetylene (TMS-acetylene), followed by desilylation. Ethynyl pseudo-nucleobases for the ^O^Pu derivatives were prepared similarly to TMS-ethyl-protected precursors.

**Figure 3:**
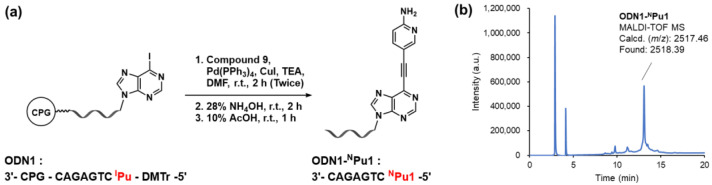
(**a**) On-column Sonogashira coupling reaction using ODN1 containing ^I^Pu and alkyne **9**. (**b**) RP-HPLC trace of crude ODN1-^N^Pu1. Conditions: 0.1 M TEAA buffer (pH 7.0) and CH_3_CN, with a gradient of 5% to 20% of CH_3_CN over 20 min. Flowrate: 1 mL/min, column oven: 50 °C, detection wavelength: 254 nm.

Having synthesized the CPG-bound ODNs containing ^I^Pu at their 5′ terminal as well as ethynyl pseudo-nucleobases, we attempted the on-column synthesis of ODNs containing alkynylated purine derivatives. Initially, we tested whether on-column Sonogashira coupling could proceed with ^I^Pu-containing ODN through the on-column construction of ^N^Pu1 using CPG-bound ODN1 and the ethynyl compound **9** (Figure 3a). The coupling was performed by treating CPG-bound ODN1 with a DMF solution containing excess equivalents of alkyne **9**, Pd(PPh_3_)_4_, copper (I) iodide, and triethylamine inside a column at ambient temperature. This reaction was conducted twice to confirm the coupling reaction. To check if the CPG-bound ODN had undergone coupling, an aliquot of the CPGs was subjected to deprotection by 28% NH_4_OH treatment at room temperature for 2 h, followed by treatment with 10% AcOH for 1 h, after which, the crude ODN was analyzed by RP-HPLC (Figure 3b). The HPLC chart showed a major peak at 13 min, which was analyzed by MALDI-TOF MS (see Supplementary Materials) to confirm the formation of the desired ODN1 with ^N^Pu1.

**Figure 4:**
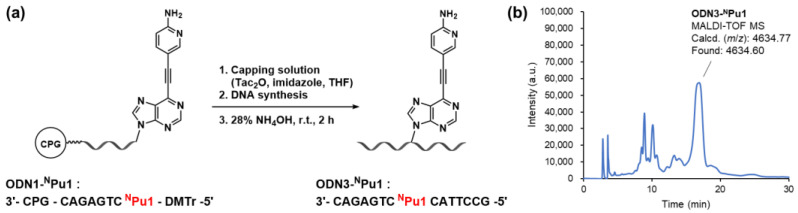
(**a**) Solid-phase synthesis and deprotection of ODN3-^N^Pu1. (**b**) The RP-HPLC trace of crude ODN1-^N^Pu3. Conditions: 0.1 M TEAA buffer (pH 7.0) and CH_3_CN, with a gradient of 9% to 10% of CH_3_CN over 30 min; flowrate: 1 mL/min, column oven: 50 °C, detection wavelength: 254 nm.

After confirming the progress of the Sonogashira coupling reaction, the column was reattached to the DNA synthesizer for elongating the remaining part of the ODN1 in the DMT-Off mode (Figure 4a). Before commencing the DNA elongation, the CPGs were treated with an excess amount of capping solution (*tert*-butylphenoxyacetyl acetic anhydride (Tac_2_O)/imidazole solution). We reasoned that this process would protect the NH_2_ group on the pyridine moiety of ^N^Pu1, which may otherwise cause branching of the ODN during DNA synthesis. Finally, the fully elongated ODN3 containing ^N^Pu1 was deprotected with 28% NH_4_OH and analyzed by RP-HPLC (Figure 4b). The major peak was isolated and analyzed by MALDI-TOF MS (see Supplementary Materials) to confirm the formation of the desired ODN3 containing ^N^Pu1. It should be noted that we also attempted the on-column Sonogashira coupling reaction on the fully elongated 15-mer ODN3 containing ^I^Pu in the middle. Although we could observe the formation of the ODNs containing the alkynylated purine derivatives, there was a non-negligible amount of the ^I^Pu-containing strand remaining. Full conversion may be achieved by the optimization of the reaction conditions; however, we did not make any further attempt in this study.

Using the same protocol, we further prepared the ODN3 and ODN4 containing the other ^N^Pu derivatives. The ^O^Pu derivatives were prepared in a similar manner, apart from the deprotection step, in which the CPGs were treated with a ZnBr_2_ solution to remove the TMS-ethyl moiety on the pseudo-nucleobase prior to the 28% NH_4_OH treatment. The purity and structural integrity of the synthesized ODNs were confirmed by RP-HPLC (Figure S1) and MALDI-TOF MS (Table 1) analyses. Taken together, these results demonstrated the utility of the post-synthesis method as a convenient approach for the preparation of ODNs containing alkynylated purine derivatives.

### 2.2. Base-Pairing Properties of the Alkynylated Purine Derivatives

Using the 15-mer ODNs incorporating the alkynylated purine derivatives, we investigated their base-pairing properties. To this end, the ODN3s and the complementary ODN4s incorporating different nucleosides at positions X and Y were annealed, and their thermal stabilities were determined by the UV melting temperature (*T*_m_) measurement (Table 2, Figure S2). In the presence of 10 mM of sodium phosphate (pH 7.0) and 50 mM of NaCl, UV melting of DNA duplexes containing canonical G–C and A–T base pairs at the X–Y position exhibited *T*_m_ values of 51.0 °C and 54.3 °C, respectively. On the other hand, the *T*_m_ values of the DNA duplexes containing previously reported ^N^Pu–^O^Pz and ^O^Pu–^N^Pz were 52.1 °C and 51.3 °C. These agree with our previous results, which show that ^N^Pu–^O^Pz and ^O^Pu–^N^Pz pairs have comparable thermal stability to the canonical base pairs and that the ^N^Pu–^O^Pz pair exhibits slightly higher thermal stability than the ^O^Pu–^N^Pz pair. We also confirmed that ^N^Pu and ^O^Pu exhibit selectivity in pairing with non-complementary pyridazine derivatives, as indicated by the decreased *T*_m_ values for ^N^Pu–^N^Pz (*T*_m_ = 48.4 °C) and ^O^Pu–^O^Pz (*T*_m_ = 48.8 °C) pairs.

Subsequently, we investigated the base-pairing properties of the newly synthesized derivatives in comparison to the previously designed ^N^Pu and ^O^Pu (Table 2). As for the ^N^Pu derivatives, ^N^Pu1–^O^Pz (*T*_m_ = 52.2 °C) and ^N^Pu3–^O^Pz (*T*_m_ = 52.7 °C) exhibited thermal stabilities similar to that of the parental ^N^Pu–^O^Pz, whereas ^N^Pu2–^O^Pz had decreased stability (*T*_m_ = 49.3 °C). ^N^Pu1 and ^N^Pu3 also exhibited similarity with ^N^Pu in terms of selectivity toward non-complementary ^N^Pz, as indicated by the lowered thermal stability of ^N^Pu1–^N^Pz (*T*_m_ = 50.2 °C) and ^N^Pu3–^N^Pz (*T*_m_ = 49.6 °C), while ^N^Pu2 could not effectively discriminate between ^N^Pz and ^O^Pz. These results indicated that the structure of the pseudo-nucleobases has a significant influence on pairing with the complementary bases of the ^N^Pu derivatives. Similar trends were reproduced with the DNA duplexes containing inverted X–Y bases (Table S1). Furthermore, all four ^N^Pu derivatives exhibited significantly low stability toward A, G, C, and T (Tables S2 and S3; *T*_m_ = 43.5–48.2 °C), suggesting that the structural differences among the pseudo-nucleobases have little impact on the selectivity toward pairing with canonical nucleobases.

Similarly, we also explored the base-pairing properties of the ^O^Pu derivatives (Table 2). Of note, we found that ^O^Pu1, bearing 2-pyrimidinone as a pseudo-nucleobase, exhibits higher affinity toward the complementary ^N^Pz (*T*_m_ = 53.2 °C). Its stability was higher than that of ^N^Pu–^O^Pz (*T*_m_ = 52.1 °C). ^O^Pu3–^N^Pz (*T*_m_ = 51.9 °C) showed thermal stability comparable to that of the ^O^Pu–^N^Pz pair (*T*_m_ = 51.3 °C), whereas ^O^Pu2–^N^Pz exhibited lower stability (*T*_m_ = 49.2 °C). ^O^Pu1 and ^O^Pu3 also showed selectivity toward non-complementary ^O^Pz (*T*_m_ = 49.5 °C and 48.8 °C, respectively), while ^O^Pu2 showed little discrimination between pairing with ^O^Pz and ^N^Pz. Similar results were obtained with the DNA duplexes containing the inverted X–Y bases (Table S1), and all three derivatives exhibited selectivity toward pairing with canonical nucleobases (Tables S2 and S3; *T*_m_ = 43.4–47.9 °C). These results again confirmed that the pseudo-nucleobases play critical roles in the formation of the alkynylated purine–pyridazine pairs and that their structures have an influence on the selectivity and stability of the alkynylated purine–pyridazine base-pairing.

### 2.3. Structural Impact of the Alkynylated Purine Derivatives on DNA Duplex

**Figure 5:**
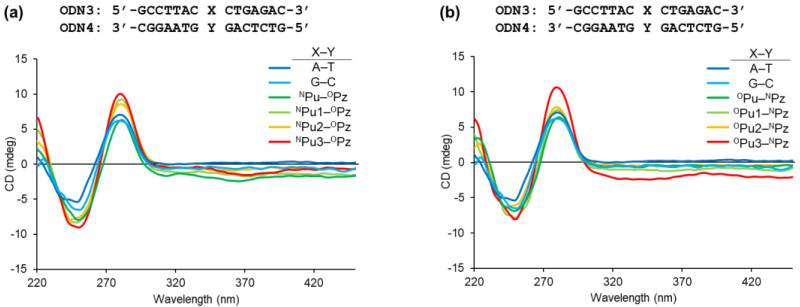
Circular dichroism (CD) spectra of the duplex DNAs containing (**a**) ^N^Pu derivatives and (**b**) ^O^Pu derivatives.

Finally, to assess the structural impact of the newly designed alkynylated purine derivatives’ pairing with the pyridazines, we conducted circular dichroism (CD) spectroscopy measurements of the duplex DNAs. The CD spectra of the fully canonical duplex DNAs indicated the formation of a typical B-type structure, characterized by a positive band at about 270–290 nm and a negative band around 240 nm (Figure 5). The duplex DNAs containing the ^N^Pu derivatives, pairing with ^O^Pz, as well as ^O^Pu derivatives pairing with ^N^Pz, exhibited similar CD spectra. Hence, these results confirmed that the duplex DNAs incorporating the alkynylated purine–pyridazine pairs do not cause significant structural disturbance of the double-helix structure, thereby demonstrating the robust base-pairing properties of the alkynylated purine and pyridazine derivatives.

## 3. Discussion

In this study, we aimed to develop a method to systematically synthesize ODNs incorporating different alkynylated purine derivatives. To this end, the on-column Sonogashira coupling reaction was investigated for the synthesis of the alkynylated purine derivatives from ^I^Pu in ODNs and the corresponding ethynyl compounds. After confirmation of the reaction progress, subsequent elongation of the remaining part of the ODNs by solid-phase DNA synthesis successfully afforded the ODNs incorporating the ^N^Pu and ^O^Pu derivatives, bearing different pseudo-nucleobase moieties. To the best of our knowledge, this study demonstrated, for the first time, the utility of ^I^Pu for on-column Sonogashira coupling with ODNs. Considering that the 6-iodopurine can be utilized as a substrate not only for Sonogashira coupling but also for other cross-coupling reactions, we believe that the present results would provide practical insights into the synthesis of various purine derivatives. We also confirmed that potential branching elongation at the amino group of the ^N^Pu derivatives can be prevented by the on-column acylation using a capping reagent prior to the DNA synthesis. This procedure may find utility when it is necessary to continue DNA solid-phase synthesis after the on-column introduction of the chemical entity endowed with a nucleophilic character.

**Figure 6:**
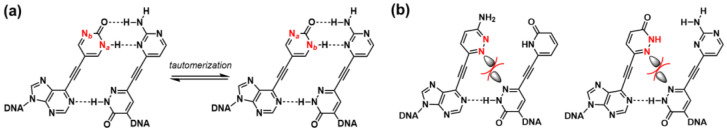
(**a**) ^O^Pu1 exhibits higher affinity due to the bifacial nature of the 2-pyrimidinone moiety. (**b**) The pyridazine pseudo-nucleobases of ^N^Pu2 and ^O^Pu2 cause static repulsion with the complementary ^N^Pz and ^O^Pz.

With the successful acquisition of ODNs incorporating ^N^Pu1, ^N^Pu2, and ^N^Pu3, as well as ^O^Pu1, ^O^Pu2, and ^O^Pu3, we further investigated their base-pairing properties. Interestingly, although their pseudo-nucleobases have similar hydrogen-bonding patterns, these derivatives exhibited different degrees of selectivity and stability as compared to the original ^N^Pu and ^O^Pu (Figure S3). In particular, ^O^Pu1 bearing 2-pyrimidinone as a pseudo-nucleobase exhibited increased stability toward ^N^Pz, with the *T*_m_ value being comparable to that of ^N^Pu–^O^Pz. This may be attributed to the bifacial hydrogen-bonding ability of 2-pyrimidinone, which increased the frequency of hydrogen bonding between the tautomerizing pseudo-nucleobases (Figure 6a). In contrast, the introduction of pyridazine-derived pseudo-nucleobases led to the loss of base-pairing selectivity and stability, as indicated by the decreased *T*_m_ values obtained with ^O^Pu2–^N^Pz and ^N^Pu–^O^Pz pairs. This could be attributed to the presence of a nitrogen atom at the 2-position of the pyridazine ring whose lone pair may cause static repulsion with the pi-orbitals of the pyridazine core structure in the opposing ^N^Pz or ^O^Pz (Figure 6b). Such repulsion would prevent the formation of stable hydrogen bonds between the pseudo-nucleobases, thereby destabilizing the base-pairing of ^O^Pu2 and ^N^Pu2 with the complementary ^N^Pz and ^O^Pz, respectively. Overall, the present study demonstrated that the structure of the pseudo-nucleobase moiety is critical for the selectivity and stability of the alkynylated purine–pyridazine base-pairing and that the on-column synthesis approach would aid in the efficient selection of the appropriate pseudo-nucleobases for the alkynylated purine nucleoside. Further structural optimization of the alkynylated purine–pyridazine base pairs is currently in progress by our research group and will be reported in due course.

## 4. Materials and Methods

### 4.1. General Information

Chemicals were purchased from Sigma-Aldrich (St. Louis, MO, USA), the Tokyo Chemical industry (Tokyo, Japan), FUJIFILM Wako Pure Chemical (Osaka, Japan), Kanto Chemical (Tokyo, Japan), and BLDpharm (Shanghai, China), and used without further purification. Reactions were conducted under argon atmosphere in oven-dried glassware unless otherwise specified. The products were isolated by column chromatography (Kanto Chemical; Silica gel 60N, spherical neutral, particle size 100–210 μm). NMR spectra were recorded on a Bruker (Billerica, MA, USA) AVANCE III 400/500/600 spectrometer. ^1^H NMR shifts were calibrated to the residual solvent: CDCl_3_ (7.26 ppm), MeOH-*d_4_* (4.87 ppm), and DMSO-*d_6_* (2.50 ppm). ^13^C NMR shifts were calibrated to the residual solvent: CDCl_3_ (77.2 ppm), MeOH-*d_4_* (49.0 ppm), and DMSO-*d_6_* (39.5 ppm). All NMR spectra were analyzed using the program Bruker TopSpin 3.6.2. The high-resolution electrospray ionization mass spectrometry measurement was performed on a Bruker MicrOTOF-QII.

### 4.2. Synthesis of the Compounds Used in This Study

*2′-Deoxy-3′,5′-di-O-acetyladenosine* (**2**): To a suspension of 2′-deoxyadenosine (4 g, 15.9 mmol) in CH_3_CN (100 mL), 4-dimethylaminopyridine (DMAP) (156 mg, 1.28 mmol), TEA (6 mL, 10.5 mmol), and acetic anhydride (3.6 mL, 38.4 mmol) were added, and the reaction mixture was stirred at room temperature for 1 h. The reaction was quenched by addition of MeOH, and volatiles were evaporated. The crude mixture was diluted with CH_2_Cl_2_. The organic layer was washed with H_2_O, brine, dried over Na_2_SO_4_, and evaporated. The residue was dissolved in a small portion of CH_2_Cl_2_ and added to a mixture of hexane-Et_2_O (1:1). The precipitate was filtered to yield compound **2** (94%, 4.93 g, 3.57 mmol) as a white powder.

^1^H NMR (400 MHz, CDCl_3_) δ 8.36 (1H, s), 7.98 (1H, s), 6.43 (1H, dd, *J* = 6.0, 8.0 Hz), 5.68 (2H, brs), 5.43 (dt, 1H, *J =* 2.4, 6.4 Hz), 4.44–4.33 (3H, m), 2.95 (1H, ddd, *J =* 6.4, 8.0, 14.0 Hz), 2.62 (1H, ddd, *J* = 2.4, 6.0, 14.0 Hz), 2.13 (3H, s), 2.09 (3H, s); ^13^C NMR (151 MHz, CDCl_3_) δ 170.6, 170.5, 155.6, 153.3, 149.8, 138.7, 120.3, 84.7, 82.7, 74.7, 63.9, 37.7, 21.1, 20.9; HRMS (ESI-TOF) Calcd. for C_14_H_18_N_5_O_5_^+^ [M + H]^+^ 336.1302, found: 336.1308.

*2′-Deoxy-3′,5′-di-O-acetyl-6-iodo-β-D-purineriboside* (**3**): To a suspension of compound **2** (4 g, 11.9 mmol) in isoamylnitrite (31 mL, 226 mmol), CH_2_I_2_ (31 mL, 357 mmol) was added, and the reaction mixture was stirred at 60 °C for 2.5 h. The reaction mixture was concentrated under reduced pressure. The residue was diluted with CH_2_Cl_2_, washed with a saturated aqueous solution of Na_2_S_2_O_3_, brine, dried over Na_2_SO_4_, and evaporated. The crude mixture was purified by silica gel column chromatography (CH_2_Cl_2_:hexane = 4:1, followed by CH_2_Cl_2_:MeOH = 100% to 200:1 to 150:1) to yield compound **3** (49%, 2.6 g, 5.83 mmol) as a pale yellow powder.

^1^H NMR (400 MHz, CDCl_3_) δ 8.64 (1H, s), 8.32 (1H, s), 6.47 (1H, dd, *J* = 6.0, 7.6 Hz), 5.45-5.44 (1H, m), 4.39–4.35 (3H, m), 2.99 (1H, ddd, *J* = 6.4, 7.6, 14.0 Hz), 2.68 (1H, ddd, *J* = 2.8, 6.0, 14.0 Hz), 2.15 (3H, s), 2.09 (3H, s); ^13^C NMR (151 MHz, CDCl_3_) δ 170.4, 170.4, 152.2, 147.5, 142.8, 139.4, 122.6, 85.3, 83.0, 63.7, 37.8, 21.0, 20.9; HRMS (ESI-TOF) Calcd. for C_14_H_16_IN_4_O_5_^+^ [M + H]^+^ 447.0160, found: 447.0155.

*2′-Deoxy-6-iodo-β-D-purineriboside* (**4**): Compound **3** (3.6 g, 8.07 mmol) was dissolved in methanolic ammonia (7 N, 300 mL) at 0 °C, and the solution was kept overnight at 4 °C. The solvent was evaporated, and the residue was recrystallized with ethanol to yield compound **4** (78%, 2.29 g, 6.33 mmol) as a white solid.

^1^H NMR (400 MHz, DMSO-*d_6_*) δ 8.86 (1H, s), 8.64 (1H, s), 6.43 (1H, t, *J* = 6.4 Hz), 5.36 (1H, d, *J* = 4.4 Hz), 4.96 (1H, t, *J* = 5.6 Hz), 4.44 (1H, dt, *J* = 3.6, 9.6 Hz), 3.88 (1H, dd, *J* = 4.4, 8.0 Hz), 3.61 (1H, dt, *J* = 5.2, 12.0 Hz), 3.53 (1H, dt, *J* = 5.6, 12.0 Hz), 2.76 (1H, ddd, *J* = 6.4, 6.6, 14.0 Hz), 2.34 (1H, ddd, *J* = 3.6, 6.4, 14.0 Hz); ^13^C NMR (151 MHz, DMSO-*d_6_*) δ 151.8, 147.4, 144.8, 138.5, 122.8, 88.1, 84.2, 70.4, 61.4, 54.0; HRMS (ESI-TOF) Calcd. for C_10_H_12_IN_4_O_3_^+^ [M + H]^+^ 362.9949, found: 362.9947.

*2′-Deoxy-5′-O-(4,4′-dimethoxytrityl)-6-iodo-β-D-purineriboside* (**5**): To a solution of compound **4** (1.15 g, 3.18 mmol) in CH_2_Cl_2_ (130 mL), DIPEA (1.5 mL, 7.94 mmol) and 4,4′-dimethoxytrityl chloride (2.15 g, 6.35 mmol) were added at 0 °C, and the reaction mixture was stirred at room temperature for 15 min. The reaction was quenched by addition of MeOH, and the mixture was concentrated under reduced pressure. The residue was diluted with CH_2_Cl_2_, washed with a saturated aqueous solution of NaHCO_3_, brine, dried over Na_2_SO_4_, and evaporated. The residue was purified by silica gel column chromatography (CH_2_Cl_2_:MeOH = 100:1 to 30:1 with 0.1% TEA) to yield compound **5** (71%, 1.49 g, 2.26 mmol) as a pale-yellow foam.

^1^H NMR (400 MHz, CDCl_3_) δ 8.54 (1H, s), 8.28 (1H, s), 7.60 (2H, dd, *J* = 1.6, 8.2 Hz), 7.30–7.21 (7H, m), 6.80 (4H, d, *J* = 8.8 Hz), 6.46 (1H, t, *J* = 6.4 Hz), 4.70 (1H, dd, *J* = 3.6, 9.6 Hz), 4.18–4.15 (1H, m), 3.79 (6H, s), 3.40 (2H, ddd, *J* = 4.4, 10.4, 18.8 Hz), 2.88 (1H, ddd, *J* = 6.4, 6.4, 13.6 Hz), 2.58 (1H, ddd, *J* = 3.6, 6.4, 13.6 Hz); ^13^C NMR (151 MHz, CDCl_3_) δ 158.8, 152.0, 147.5, 144.5, 143.2, 139.4, 135.7, 135.6, 130.1, 128.2, 128.1, 127.2, 122.4, 113.4, 86.7, 86.5, 85.1, 72.8, 63.7, 55.4, 40.4, 0.1; HRMS (ESI-TOF) Calcd. for C_31_H_29_IN_4_NaO_5_^+^ [M + Na]^+^ 687.1075, found: 687.1077.

*2′-Deoxy-3′-O-[2-cyanoethoxy(diisopropylamino)phosphino]-5′-O-(4,4′-dimethoxytrityl)-6-iodo-β-D-purineriboside* (**6**): To a solution of compound **5** in CH_2_Cl_2_ (3 mL), *N*,*N*-diisopropylethylamine (DIPEA) (157 µL, 0.902 mmol) and 2-cyanoethyl-*N,N*-diisopropylchlorophosphoramidite (100 µL, 0.451 mmol) were added at 0 °C, and the reaction mixture was stirred at room temperature for 1.5 h. The reaction mixture was diluted with AcOEt. The organic layer was washed with a saturated aqueous solution of NaHCO_3_ and brine, dried over Na_2_SO_4_, and concentrated. The residue was purified by flash chromatography (hexane:AcOEt = 2:1 to 1:1) to yield compound **6** (70%, 145 mg, 0.105 mmol) as a white foam.

^31^P NMR (162 MHz, CDCl_3_) δ 149.0, 148.8; HRMS (ESI-TOF) Calcd. for C_40_H_47_IN_6_O_6_P^+^ [M + H]^+^ 865.2334, found: 865.2312.

*2-Amino-5-(trimethylsilyl)ethynylpyridine* (**8**): To a solution of 2-amino-5-iodopyridine **7** (2.2 g, 10.0 mmol), Pd(PPh_3_)_2_Cl_2_ (351 mg, 0.5 mmol), and CuI (95 mg, 0.5 mmol) in CH_3_CN (83 mL), TEA (8.3 mL, 60.0 mmol) and TMS-acetylene (3.1 mL, 20.0 mmol) were added, and the mixture was stirred at room temperature for 30 min. The reaction mixture was filtered through a pad of celite, and the filtrate was evaporated under reduced pressure. The crude material was purified by silica gel column chromatography (CH_2_Cl_2_:AcOEt = 100% to 5:1) to yield compound **8** (96%, 1.83 g, 9.62 mmol) as a brown solid.

^1^H NMR (400 MHz, CDCl_3_) δ 8.21 (1H, dd, *J* = 0.8, 2.4 Hz), 7.50 (1H, dd, *J* = 0.8, 8.4 Hz), 6.42 (1H, dd, *J* = 0.8 Hz, 8.4 Hz), 4.59 (2H, brs), 0.24 (9H, s); ^13^C NMR (151 MHz, CDCl_3_) δ 157.7, 152.1, 140.9, 109.9, 107.9, 102.9, 94.7, 0.2; HRMS (ESI-TOF) Calcd. C_10_H_15_N_2_Si^+^ [M + H]^+^ 191.0999, found: 191.1008.

*2-Amino-5-ethynylpyridine* (**9**): To a solution of compound **8** (500 mg, 2.63 mmol) in MeOH (29 mL), K_2_CO_3_ (689 mg, 5.25 mmol) was added, and the reaction mixture was stirred at room temperature. After 45 min, the reaction mixture was diluted with AcOEt and filtered through a pad of celite. The filtrate was washed with H_2_O and brine, dried over Na_2_SO_4_, and evaporated under reduced pressure to yield compound **9** (92%, 284 mg, 2.40 mmol) as an orange powder. 

^1^H NMR (400 MHz, CDCl_3_) δ 8.23 (1H, d, *J* = 1,6 Hz), 7.51 (1H, dd, *J* = 2.0, 8.4 Hz), 6.43 (1H, dd, *J* = 0.8 Hz, 8.4 Hz), 4.59 (2H, brs), 3.06(1H, s); ^13^C NMR (151 MHz, CDCl_3_) δ 157.9, 152.3, 141.0, 108.9, 107.9, 81.5, 77.8; HRMS (ESI-TOF) Calcd. C_7_H_7_N_2_^+^ [M + H]^+^ 119.0604, found: 119.0610.

*3-Amino-6-(trimethylsilyl)ethynylpyridazine* (**11**): To a solution of 6-iodo-3-amino pyridazine **10** (1.0 g, 4.52 mmol), Pd(PPh_3_)_2_Cl_2_ (159 mg, 0.226 mmol), and CuI (43 mg, 0.226 mmol) in CH_3_CN (38 mL), TEA (3.8 mL, 27.1 mmol) and TMS-acetylene (1.4 mL, 9.01 mmol) were added, and the mixture was stirred at room temperature for 18 h. The reaction mixture was filtered through a pad of celite, and the filtrate was evaporated under reduced pressure. The crude material was purified by silica gel column chromatography (CH_2_Cl_2_:AcOEt = 100% to 1:1) to yield compound **11** (61%, 528 mg, 2.76 mmol) as a yellow solid.

^1^H NMR (400 MHz, CDCl_3_) δ 7.31 (1H, d, *J* = 9.2 Hz), 6.67 (1H, d, *J* = 9.2 Hz), 4.85 (2H, brs), 0.27 (9H, s); ^13^C NMR (151 MHz, CDCl_3_) δ 158.5, 139.8, 131.6, 113.6, 101.4, 96.9, −0.1; HRMS (ESI-TOF) Calcd. C_9_H_14_N_3_Si^+^ [M + H]^+^ 192.0952, found: 192.0953.

*3-Amino-6-ethynylpyridazine* (**12**): Compound **11** (240 mg, 1.25 mmol) was dissolved in methanolic ammonia (2.0 M, 24 mL). After 30 min, the reaction mixture was evaporated under reduced pressure. The residue was triturated with hexane to yield compound **12** (84%, 125 mg, 1.05 mmol) as a brown powder.

^1^H NMR (400 MHz, DMSO-*d_6_*) δ 7.10 (1H, d, *J* = 9.2 Hz), 6.53 (2H, brs) 6.47 (1H, d, *J* = 9.2 Hz), 4.06 (1H, s); ^13^C NMR (151 MHz, DMSO-*d_6_*) δ 159.4, 136.9, 130.8, 112.7, 81.5, 81.0; HRMS (ESI-TOF) Calcd. C_6_H_6_N_3_^+^ [M + H]^+^ 120.0556, found: 120.0561.

*2-Amino-5-(trimethylsilyl)ethynylpyrazine* (**14**): To a solution of 2-amino-5-bromopyrazine **13** (200 mg, 1.15 mmol), Pd(PPh_3_)_2_Cl_2_ (40 mg, 5.75 μmol), and CuI (11 mg, 5.75 μmol) in CH_3_CN (9.6 mL), TEA (1 mL, 6.90 mmol) and TMS-acetylene (353 μL, 2.30 mmol) were added, and the mixture was refluxed for 3 h. The reaction mixture was filtered through a pad of celite, and the filtrate was evaporated under reduced pressure. The crude material was purified by silica gel column chromatography (CH_2_Cl_2_:AcOEt = 100% to 8:1) to yield compound **14** (93%, 204 mg, 1.07 mmol) as an orange solid.

^1^H NMR (400 MHz, CDCl_3_) δ 8.15 (1H, d, *J* = 1.6 Hz), 7.93 (1H, d, *J* = 1.6 Hz), 4.72 (2H, brs), 0.25 (9H, s); ^13^C NMR (151 MHz, CDCl_3_) δ 153.0, 145.9, 132.1, 128.8, 101.7, 95.7, 0.0; HRMS (ESI-TOF) Calcd. C_9_H_14_N_3_Si^+^ [M + H]^+^ 192.0952, found: 192.0961.

*2-Amino-5-ethynylpyrazine* (**15**): To a solution of compound **14** (690 mg, 3.61 mmol) in MeOH (40 mL), K_2_CO_3_ (996 mg, 7.22 mmol) was added, and the reaction mixture was stirred at room temperature. After 5 min, the reaction mixture was diluted with AcOEt and filtered through a pad of celite. The filtrate was washed with H_2_O and brine, dried over Na_2_SO_4_, and evaporated under reduced pressure to yield compound **15** (97%, 416 mg, 3.49 mmol) as a brown powder. 

^1^H NMR (400 MHz, CDCl_3_) δ 8.17 (1H, d, *J* = 1.6 Hz), 7.95 (1H, d, *J* = 1.6 Hz), 4.82 (2H, brs), 3.17 (1H, s); ^13^C NMR (151 MHz, CDCl_3_) δ 153.3, 146.2, 132.3, 128.0, 80.8, 78.1; HRMS (ESI-TOF) Calcd. C_6_H_6_N_3_^+^ [M + H]^+^ 120.0556, found: 120.0593.

*5-Iodo-2-[2-(trimethylsilyl)ethoxy]pyrimidine* (**17**): To a suspension of NaH (166 mg, 4.16 mmol) in THF (2.8 mL), TMS ethanol (593 μL, 4.16 mmol) was added at 0 °C, and the mixture was stirred under argon at room temperature until the H_2_ gas evolution stopped. To the stirred suspension, a solution of 2-chloro-5-iodopyrimidine **16** (500 mg, 2.08 mmol) in THF (2.8 mL) was added at room temperature. After 30 min, the reaction was quenched with a saturated aqueous solution of NH_4_Cl. The solution was extracted with AcOEt, and the organic layer was washed with brine, dried over Na_2_SO_4_, and concentrated under reduced pressure. The residue was diluted with AcOEt, washed with brine, dried over Na_2_SO_4_, and concentrated under reduced pressure. The residue was purified by silica gel column chromatography (hexane:AcOEt = 30:1 to 15:1) to yield compound **17** (80%, 536 mg, 1.66 mmol) as a pale-yellow gel.

^1^H NMR (400 MHz, CDCl_3_) δ 8.52 (2H, s), 4.44–4.39 (2H, m), 1.20–1.15 (2H, m), 0.08 (9H, s); ^13^C NMR (151 MHz, CDCl_3_) δ 164.4, 164.3, 82.1, 66.5, 17.5, −1.3; HRMS (ESI-TOF) Calcd. C_9_H_16_IN_2_OSi^+^ [M + H]^+^ 323.0071, found: 323.0059.

*5-(Trimethylsilyl)ethynyl-2-[2-(trimethylsilyl)ethoxy]pyrimidine* (**18**): To a solution of compound **17** (337 mg, 1.05 mmol), triphenylphosphine (6 mg, 20.9 μmol), Pd(PPh_3_)_2_Cl_2_ (14 mg, 20.9 μmol), and CuI (6 mg, 20.9 μmol) in DIPEA (7.5 mL), TMS-acetylene (149 μL, 1.05 mmol) was added, and the mixture was stirred at room temperature for 2 h. The reaction mixture was filtered through a pad of celite, and the filtrate was evaporated under reduced pressure. The crude material was purified by silica gel column chromatography (hexane:AcOEt = 50:1 to 30:1) to yield compound **18** (98%, 302 mg, 1.03 mmol) as a pale-yellow gel. 

^1^H NMR (400 MHz, CDCl_3_) δ 8.55 (2H, s), 4.47–4.43 (2H, m), 1.20–1.16 (2H, m), 0.25 (9H, s), 0.08 (9H, s); ^13^C NMR (151 MHz, CDCl_3_) δ 163.8, 161.9, 112.6, 99.8, 99.3, 66.4, 17.6, −0.03, −1.3; HRMS (ESI-TOF) Calcd. C_14_H_25_N_2_OSi_2_^+^ [M + H]^+^ 293.1500, found: 293.1500.

*5-Ethynyl-2-[2-(trimethylsilyl)ethoxy]pyrimidine* (**19**): To a solution of compound **18** (150 mg, 0.513 mmol) in MeOH (5.1 mL), K_2_CO_3_ (142 mg, 1.03 mmol) was added at room temperature. After 10 min, the reaction mixture was diluted with CH_2_Cl_2_. The organic layer was washed with H_2_O and brine, dried over Na_2_SO_4_, and evaporated under reduced pressure to yield compound **19** (97%, 110 mg, 0.499 mmol) as a pale-yellow gel.

^1^H NMR (400 MHz, CDCl_3_) δ 8.59 (2H, s), 4.49–4.45 (2H, m), 1.21–1.17 (2H, m), 0.08 (9H, s); ^13^C NMR (151 MHz, CDCl_3_) δ 164.1, 162.1, 111.6, 82,2, 66.5, 17.6, −1; HRMS (ESI-TOF) Calcd. C_11_H_17_N_2_OSi^+^ [M + H]^+^ 221.1105, found: 221.1100.

*3-Iodo-6-[2-(trimethylsilyl)ethoxy]pyridazine* (**21**): To a suspension of NaH (144 mg, 3.62 mmol) in THF (3.5 mL), TMS ethanol (516 μL, 3.62 mmol) was added at 0 °C, and the mixture was stirred under argon at room temperature until the H_2_ gas evolution stopped. To the stirred suspension, a solution of 3,6-diiodopyridazine **20** (800 mg, 2.41 mmol) in THF (3.5 mL) was added at room temperature. After 30 min, the reaction was quenched with a saturated aqueous solution of NH_4_Cl. The solution was extracted with AcOEt, and the organic layer was washed with brine, dried over Na_2_SO_4_, and concentrated under reduced pressure. The residue was purified by silica gel column chromatography (hexane:AcOEt = 30:1 to 5:1) to yield compound **21** (87%, 675 mg, 2.09 mmol) as a pale-yellow gel.

^1^H NMR (400 MHz, CDCl_3_) δ 7.62 (1H, d, *J* = 9.1 Hz), 6.63 (1H, d, *J* = 9.1 Hz), 4.59–4.54 (2H, m), 1.19–1.15 (2H, m), 0.07 (9H, s); ^13^C NMR (151 MHz, CDCl_3_) δ 164.9, 139.3, 119.5, 116.6, 66.2, 17.6, −1.2; HRMS (ESI-TOF) Calcd. C_9_H_16_IN_2_OSi^+^ [M + H]^+^ 323.0071, found: 323.0043.

*6-(Trimethylsilyl)ethynyl-3-[2-(trimethylsilyl)ethoxy]pyridazine* (**22**): To a solution of compound **21** (600 mg, 1.86 mmol), Pd(PPh_3_)_2_Cl_2_ (65 mg, 0.093 mmol), and CuI (35 mg, 0.186 mmol) in THF (5.3 mL), TEA (2.1 mL, 14.9 mmol) and TMS-acetylene (0.4 mL, 2.79 mmol) were added, and the mixture was stirred at room temperature for 3 h. The reaction mixture was filtered through a pad of celite, and the filtrate was evaporated under reduced pressure. The crude material was purified by silica gel column chromatography (hexane:AcOEt = 100:1 to 30:1) to yield compound **22** (86%, 468 mg, 1.60 mmol) as a pale-yellow gel.

^1^H NMR (400 MHz, CDCl_3_) δ 7.42 (1H, d, *J* = 9.2 Hz), 6.84 (1H, d, *J* = 9.2 Hz), 4.65–4.61 (2H, m), 1.20–1.16 (2H, m), 0.28 (9H, s), 0.07 (9H, s); ^13^C NMR (151 MHz, CDCl_3_) δ 163.8, 161.9, 112.6, 99.8, 98.3, 66.4, 17.6, −0.03, −1.3; HRMS (ESI-TOF) Calcd. C_14_H_25_N_2_OSi_2_^+^ [M + H]^+^ 293.1500, found: 293.1506.

*6-Ethynyl-3-[2-(trimethylsilyl)ethoxy]pyridazine* (**23**): To a solution of compound **22** (35 mg, 0.119 mmol) in MeOH (1.2 mL), K_2_CO_3_ (33 mg, 0.238 mmol) was added, and the reaction mixture was stirred at room temperature. After 5 min, the reaction mixture was diluted with CH_2_Cl_2_, filtered through a pad of celite, washed with H_2_O and brine, dried over Na_2_SO_4_, and evaporated under reduced pressure to yield compound **23** (25 mg, 95%) as a brown gel.

^1^H NMR (400 MHz, CDCl_3_) δ 7.45 (1H, d, *J* = 9.1 Hz), 6.84 (1H, d, *J* = 9.1 Hz), 4.67–4.62 (2H, m), 3.29 (1H, s) 1.25–1.17 (2H, m), 0.08 (9H, s); ^13^C NMR (151 MHz, CDCl_3_) δ 163.9, 142.4, 132.6, 116.8, 80.2, 80.1, 66.3, 17.7, −1.2; HRMS (ESI-TOF) Calcd. C_11_H_17_N_2_OSi^+^ [M + H]^+^ 221.1105, found: 221.1104.

*5-(Trimethylsilyl)ethynyl-2-[2-(trimethylsilyl)ethoxy]pyrazine* (**27**): To a suspension of NaH (84 mg, 2.11 mmol) in THF (2 mL), TMS ethanol (300 μL, 2.11 mmol) was added at 0 °C, and the mixture was stirred under argon at room temperature until the H_2_ gas evolution stopped. To the stirred suspension, a solution of 2-bromo-5-iodopyrazine **24** in THF (2 mL) was added at room temperature. After 15 h, the reaction was quenched with a saturated aqueous solution of NH_4_Cl. The solution was extracted with AcOEt, and the organic layer was washed with brine, dried over Na_2_SO_4_, and concentrated under reduced pressure. The residue was purified by silica gel column chromatography (hexane:AcOEt = 100% to 10:1) to yield a mixture of 2-bromo-5-(2-(trimethylsilyl)ethoxy)pyrazine **25** and 2-iodo-5-(2-(trimethylsilyl)ethoxy)pyrazine **26** (387 mg, ratio of 1:4, as determined by NMR). Subsequently, to a solution of the mixture (150 mg, 0.465 mmol), Pd(PPh_3_)_2_Cl_2_ (18 mg, 24 μmol), and CuI (9 mg, 48 μmol) in THF (1.2 mL), TMS-acetylene (1.32 mL 9.30 mmol) and TEA (546 μL, 3.72 mmol) were added, and the reaction mixture was stirred for 15 h. The reaction mixture was filtered through a pad of celite, and the filtrate was evaporated under reduced pressure. The residue was dissolved in AcOEt, and the organic layer was washed with H_2_O and brine, dried over Na_2_SO_4_, and evaporated. The crude material was purified by silica gel column chromatography (hexane:Et_2_O = 100% to 100:1) to yield compound **27** (100 mg, 75% over 2 steps) as a pale-yellow gel.

^1^H NMR (400 MHz, MeOH-*d_4_*) δ 8.26 (1H, d, *J* = 1.6 Hz), 8,08 (1H, d, *J* = 1.6 Hz), 4.50–4.46 (2H, m), 1.18–1.14 (2H, m), 0.26 (9H, s), 0.08 (9H, s); ^13^C NMR (151 MHz, MeOD-*d_4_*) δ 160.8, 145.9, 136.6, 132.0, 102.0, 97.2, 66.3, 18.3, −0.2, −1.3; HRMS (ESI-TOF) Calcd. C_14_H_25_N_2_OSi_2_^+^ [M + H]^+^ 293.1500, found: 293.1506.

*5-Ethynyl-2-[2-(trimethylsilyl)ethoxy]pyrazine* (**28**): To a solution of compound **27** (50 mg, 0.171 mmol) in MeOH (2 mL), K_2_CO_3_ (45 mg, 0.342 mmol) was added, and the reaction mixture was stirred at room temperature. After 5 min, the reaction mixture was diluted with CH_2_Cl_2_, filtered through a pad of celite, washed with H_2_O and brine, dried over Na_2_SO_4_, and evaporated under reduced pressure to yield compound **28** (96%, 36 mg, 0.163 mmol) as an orange solid. 

^1^H NMR (400 MHz, MeOH-*d_4_*) δ 8.27 (1H, d, *J* = 1.4 Hz), 8.09 (1H, d, *J* = 1.4 Hz), 4.49–4.45 (2H, m), 3.74 (1H, s), 1.17–1.13 (2H, m), 0.08 (9H, s); ^13^C NMR (151 MHz, MeOD-*d_4_*) δ 160.9, 146.0, 136.7, 131.5, 80.9, 80.8, 66.3, 18.2, −1.3; HRMS (ESI-TOF) Calcd. C_11_H_17_N_2_OSi^+^ [M + H]^+^ 221.1105, found: 221.1095.

### 4.3. Solid-Phase DNA Synthesis

The oligonucleotides were synthesized on a 1 μmol scale using a DNA automated synthesizer (Applied Biosystems, Waltham, MA, USA, 392 DNA/RNA Synthesizer) with conventional phosphoramidite chemistry. The reagents were purchased from Glen Research (Sterling, VA, USA) and Sigma-Aldrich, and the synthesis was conducted using ultra-mild phosphoramidites (phenoxyacetyl-dA (Pac-dA), Tac-dG, Ac-dC, T), with 0.25 M 5-benzylthio-1*H*-tetrazole (BTT) in CH_3_CN as an activator, 3% dichloroacetic acid (DCA) in CH_2_Cl_2_ as a deblocking solution, and 5% Tac_2_O and 16% *N*-methylimidazole in THF as a capping reagent. The ODNs incorporating the original ^N^Pu and ^O^Pu were synthesized as previously reported [31]. Fully natural ODNs were purchased from Japan Bio Service Co., Ltd. (Osaka Japan).

### 4.4. On-Column Synthesis of ODNs Incorporating the Alkynylated Purine Derivatives

Under argon atmosphere, Solution A containing CuI (7.5 mM) in a mixture of TEA-DMF (3:7), and Solution B containing each alkynylated pseudo-nucleobase (22.5 mM) and Pd(PPh_3_)_4_ (7.5 mM) in a mixture of TEA-DMF (3:7), were prepared and degassed through argon bubbling for 30 min. Solution A (100 µL) and Solution B (400 µL) were transferred to individual disposable syringes and attached onto the column containing CPG-bound ODN1 or ODN2 (DMT-ON, 0.2 µmol). The reaction was initiated by mixing the solution inside the column. After 2 h at room temperature, the solution was discarded, and the CPGs were washed sequentially with DMF, CH_3_CN, H_2_O, EDTA solution (1.0 M in H_2_O, pH 8.0), H_2_O, and CH_3_CN. The same procedure was performed twice. After drying the CPGs under a vacuum overnight, the remaining part of the ODNs was elongated using the DNA synthesizer, as described in the previous section. In case of synthesizing ODNs containing the ^N^Pu derivatives, the CPGs were treated with capping solution on the DNA synthesizer five times (60 s each) for protecting the NH_2_ group prior to the elongation.

### 4.5. Deprotection and Purification of the Synthesized ODNs

The CPG-bound ODNs incorporating ^N^Pu derivatives were treated with 28% NH_4_OH (1 mL) at room temperature for 2 h. The CPGs were filtered off, and the filtrate was evaporated using a centrifugal evaporator. The ODNs containing ^O^Pu derivatives were deprotected in a similar manner, except that the CPGs were treated with zinc bromide solution (500 μL, prepared by dissolving 2.5 g of ZnBr_2_ in a mixture of 1.5 mL of *i*-PrOH and 1.5 mL of CH_3_NO_2_) at room temperature for 6 h prior to NH_4_OH treatment. The crude oligonucleotides were purified by reverse-phase HPLC using a JASCO HPLC system (PU-2089 plus, UV-2075 plus, CO-2067 plus) equipped with a Nacalai Tesque COSMOSIL 5C_18_-MS-II column (4.6 × 250 mm). The column oven was set to 50 °C and a peak was detected at 254 nm. The following buffer system was used: buffer A: 0.1 M triethylammonium acetate (TEAA), pH 7.0 in H_2_O, buffer B: acetonitrile. A flowrate of 1 mL/min with a gradient of 9% to 10% of buffer B in 30 min was applied for the purification. The structural integrity of the synthesized oligonucleotides was analyzed by MALDI-TOF mass measurement using a Bruker Daltonics Autoflex Speed instrument, with a mixture of 3-hydroxypicolinic acid and diammonium hydrogen citrate as a matrix.

### 4.6. UV Melting Temperature Measurement of the Duplex DNAs

A solution (100 µL) of equimolar amounts of ODN3 and complementary ODN4 (2 µM each) in the buffer solution containing 10 mM of sodium phosphate buffer (pH 7.0) and 50 mM of NaCl was heated at 85 °C and gradually cooled down to room temperature for annealing. UV melting curves were recorded with a quartz cell with a 1 cm path length at temperatures between 20 and 85 °C using the JASCO V-730 UV-visible spectrophotometer (Jasco, Oklahoma City, OK, USA), with a temperature controller at a ramping and scanning rate of 1.0 °C/min at 260 nm. Each *T*_m_ value is presented as an average of three measurements.

### 4.7. CD Spectroscopy Measurement of the Duplex DNAs

The DNA solutions for CD measurement were prepared as described above. CD spectra were recorded on a JASCO J-720WI circular dichroism spectrometer equipped with a Peltier temperature controller using a micro quartz cell with a 1 cm path length. The ellipticity was recorded at 25 °C with wavelengths from 450 to 220 nm.

## Figures and Tables

**Table 1:** Sequences of the synthesized ODNs incorporating ^N^Pu and ^O^Pu derivatives.

Sequence Name	Sequence	Calcd. for [M − H]^−^	Found (*m*/*z*)
ODN3−^**N**^**Pu1**	5′-GCCTTAC^**N**^**Pu1** CTGAGAC	4634.77	4634.59
ODN3−^**N**^**Pu2**	5′-GCCTTAC^**N**^**Pu2** CTGAGAC	4635.76	4636.06
ODN3−^**N**^**Pu3**	5′-GCCTTAC^**N**^**Pu3** CTGAGAC	4635.76	4635.93
ODN3−^**O**^**Pu1**	5′-GCCTTAC^**O**^**Pu1** CTGAGAC	4636.75	4636.97
ODN3−^**O**^**Pu2**	5′-GCCTTAC ^**O**^**Pu2** CTGAGAC	4636.75	4636.35
ODN3−^**O**^**Pu3**	5′-GCCTTAC ^**O**^**Pu3** CTGAGAC	4636.75	4636.22
ODN4−^**N**^**Pu1**	5′-GTCTCAG ^**N**^**Pu1** GTAAGGC	4714.79	4714.96
ODN4−^**N**^**Pu2**	5′-GTCTCAG^**N**^**Pu2** GTAAGGC	4715.78	4715.39
ODN4−^**N**^**Pu3**	5′-GTCTCAG ^**N**^**Pu3** GTAAGGC	4715.78	4716.29
ODN4−^**O**^**Pu1**	5′-GTCTCAG ^**O**^**Pu1** GTAAGGC	4718.97	4719.41
ODN4−^**O**^**Pu2**	5′-GTCTCAG ^**O**^**Pu2** GTAAGGC	4716.77	4716.46
ODN4−^**O**^**Pu3**	5′-GTCTCAG ^**O**^**Pu3** GTAAGGC	4716.77	4716.07

**Table 2:** *T*_m_ values of the duplex DNAs incorporating canonical as well as ^N^Pu and ^O^Pu derivatives *^a^*.

ODN3: 5’-GCCTTAC X CTGAGAC-3’ ODN4: 3’-CGGAATG Y GACTCTG-5’	Base Pair (X–Y)	*T*_m_ (°C)
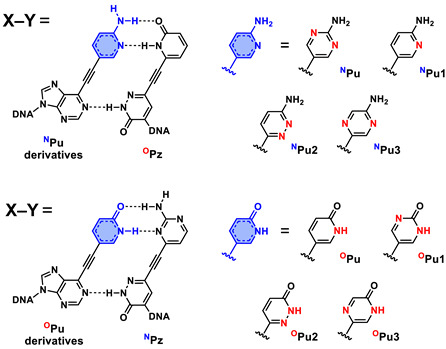	**A**–**T**	51.0 ± 0.1
	**G**–**C**	54.3 ± 0.1
	**^N^Pu**–**^O^Pz**	52.1 ± 0.1
	**^N^Pu1**–**^O^Pz**	52.2 ± 0.2
	**^N^Pu2**–**^O^Pz**	49.3 ± 0.1
	**^N^Pu3**–**^O^Pz**	52.7 ± 0.2
	**^N^Pu**–**^N^Pz**	48.4 ± 0.1
	**^N^Pu1**–**^N^Pz**	50.2 ± 0.2
	**^N^Pu2**–**^N^Pz**	49.0 ± 0.1
	**^N^Pu3**–**^N^Pz**	49.6 ± 0.1
	**^O^Pu**–**^N^Pz**	51.3 ± 0.1
	**^O^Pu1**–**^N^Pz**	53.2 ± 0.1
	**^O^Pu2**–**^N^Pz**	49.2 ± 0.1
	**^O^Pu3**–**^N^Pz**	51.9 ± 0.2
	**^O^Pu**–**^O^Pz**	48.8 ± 0.1
	**^O^Pu1**–**^O^Pz**	49.5 ± 0.7
	**^O^Pu2**–**^O^Pz**	48.3 ± 0.1
	**^O^Pu3**–**^O^Pz**	48.8 ± 0.2

*^a^* Conditions: 2 µM duplex DNA, 10 mM sodium phosphate, 50 mM NaCl, pH 7.0.

## Data Availability

Not applicable.

## Supplementary Materials

The following supporting information can be downloaded at: https://www.mdpi.com/article/10.3390/molecules28041766/s1. Figure S1: HPLC charts; Figure S2: *T*_m_ curves; Figure S3: Speculated recognition modes of the base pairs; NMR charts and MALDI-TOF MS spectra; Tables S1–S3: Summary of *T*_m_ values.
