# Supplementary material for: Synthesis of 6-Alkynylated Purine-Containing DNA via On-Column Sonogashira Coupling and Investigation of Their Base-Pairing Properties
Source: Molecules. 2023 Feb 13;28(4):1766. doi: 10.3390/molecules28041766 (PMC9965804; doi:10.3390/molecules28041766)
Supplement: Supplementary file 1 [file molecules-28-01766-s001.zip › molecules-2206301-supplementary.pdf]

Supporting information

Synthesis of 6-alkynylated purine-containing DNA via on-column Sonogashira coupling and investigation of their base pairing properties

Hidenori Okamura[1,2,*], Giang Hoang Trinh[1,2], Zhuoxin Dong[1,2], Wenjue Fan[1,2], and Fumi Nagatsugi[1,2,*]

[a]Institute of Multidisciplinary Research for Advanced Materials, Tohoku University, 2-1-1 Katahira, Aoba-ku, Sendai, Miyagi 980-8577, Japan

[b]Department of Chemistry, Graduate School of Science, Tohoku University, Aoba-ku, Sendai, Miyagi 980-8578, Japan

E-mail: hidenori.okamura.b8@tohoku.ac.jp, fumi.nagatsugi.b8@tohoku.ac.jp

1. HPLC analyses of the synthesized ODNs 2

2. UV melting curves and Tm values of the duplex DNAs 3

3. Speculated recognition modes of the alkynylated purine derivatives 8

4. ^{1}H, ^{13}C and ^{31}P NMR spectra of the synthesized compounds 9

5. MALDI-TOF MS charts of the synthesized ODNs 28

1. HPLC analyses of the synthesized ODNs

The purified ODN3 and ODN4 incorporating ^{N}Pu and ^{O}Pu derivatives were analyzed by reverse-phase HPLC using a JASCO HPLC system (PU-2089 plus, UV-2075 plus, CO-2067 plus) equipped with a Nacalai Tesque COSMOSIL 5C$_{18}$-MS-II column (4.6 × 250 mm). The column oven was set to 50 °C and peak was detected at 254 nm. The following buffer system was used: buffer A: 0.1 M TEAA, pH 7.0 in H_2O, buffer B: acetonitrile. A flow rate of 1 ml/min with a gradient of 5 to 20% of buffer B in 25 min was applied.

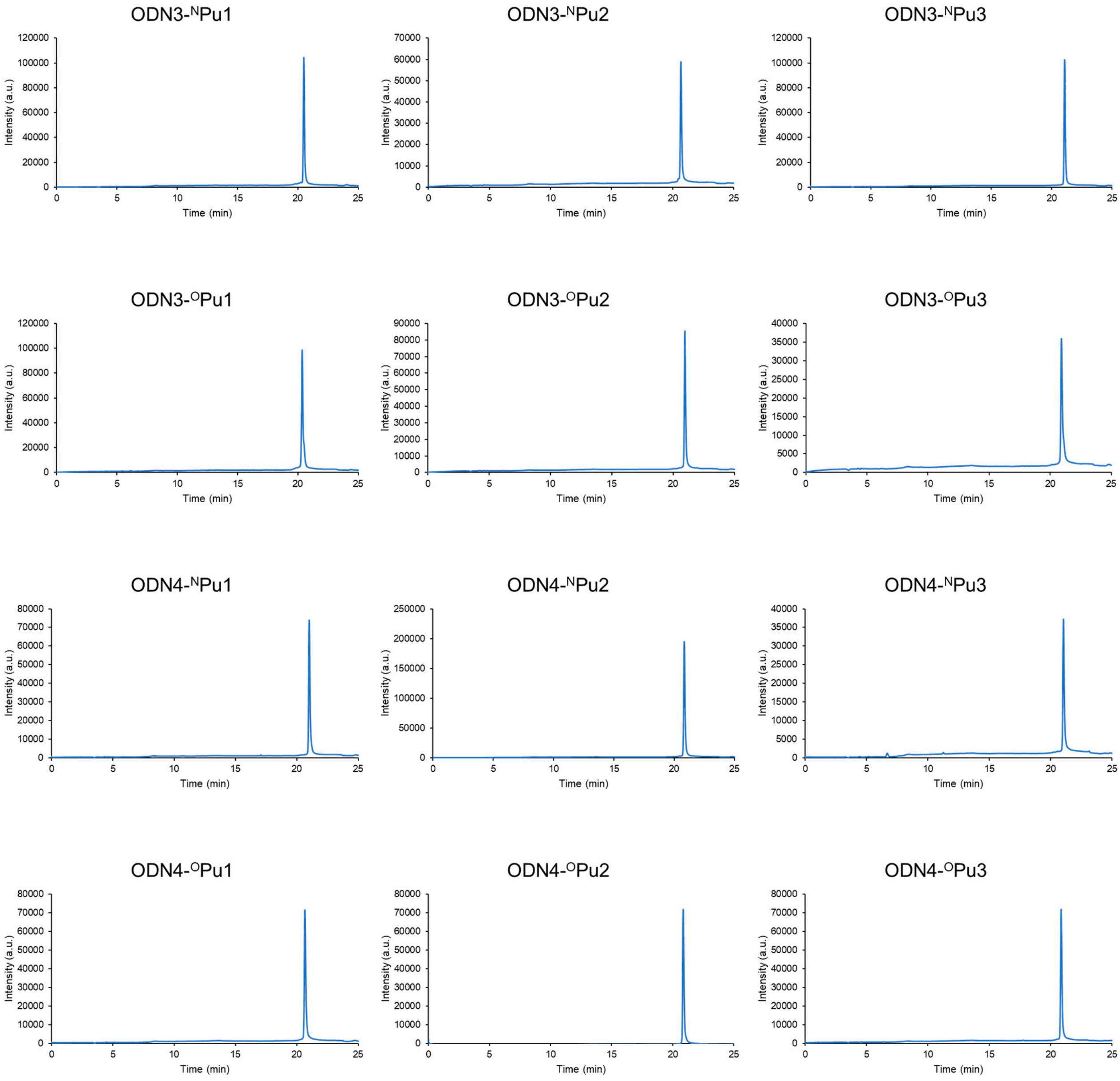

Figure S1. HPLC charts of the purified ODN3 and ODN4 incorporating the alkynylated purine derivatives.

2. UV melting curves and T_m values of the duplex DNAs

OND3: 5′-GCCTTAC**X**CTGAGAC-3′
ODN4: 3′-CGGAATG**Y**GACTCTG-5′

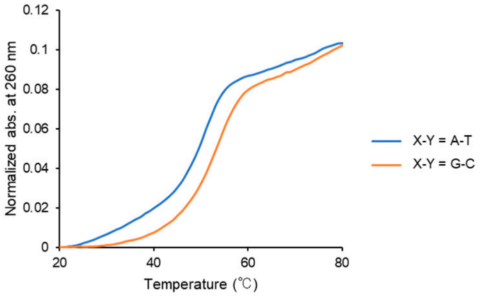

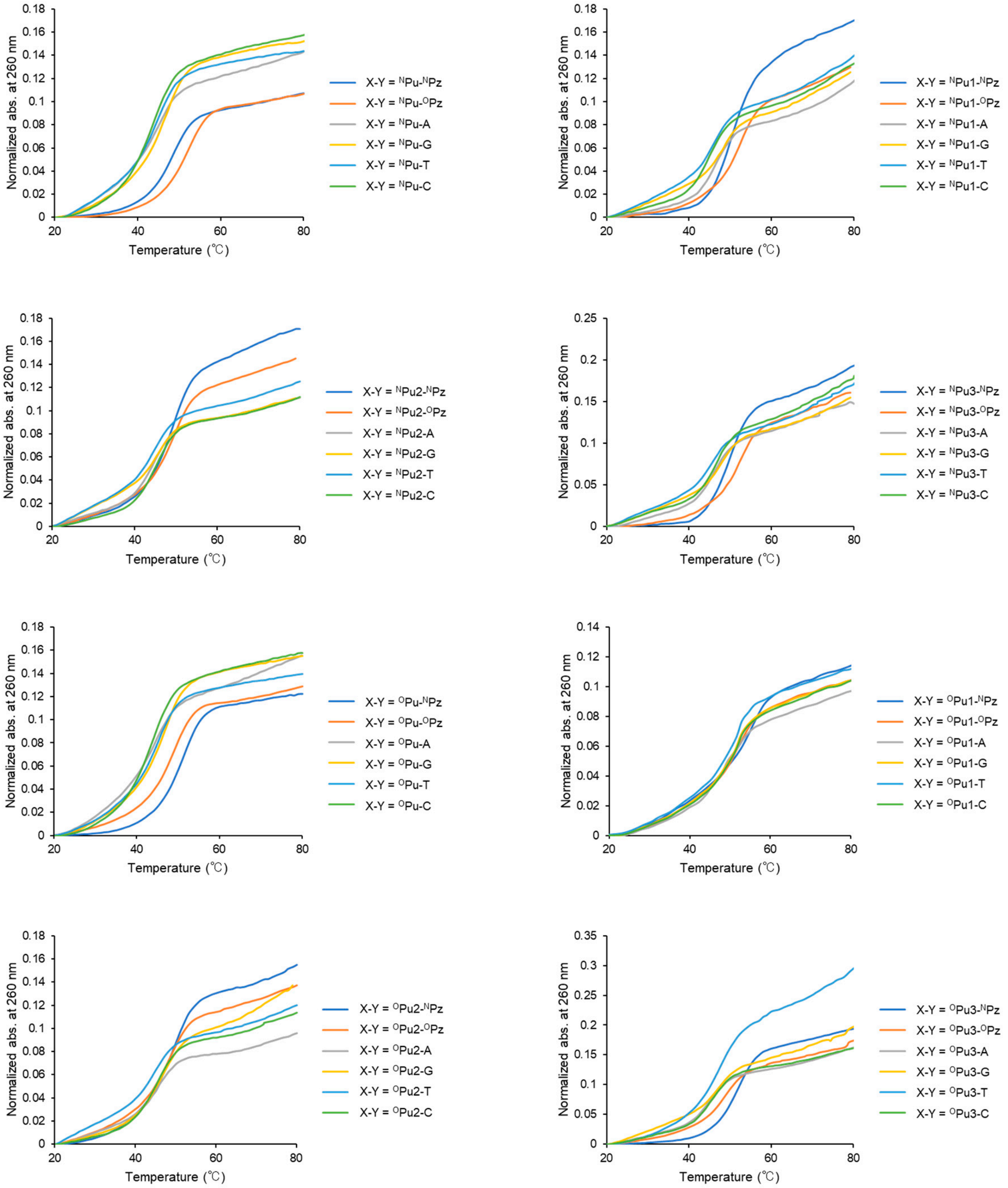

Figure S2. UV melting curves of the duplex DNAs incorporating different combinations of X-Y base pairs.

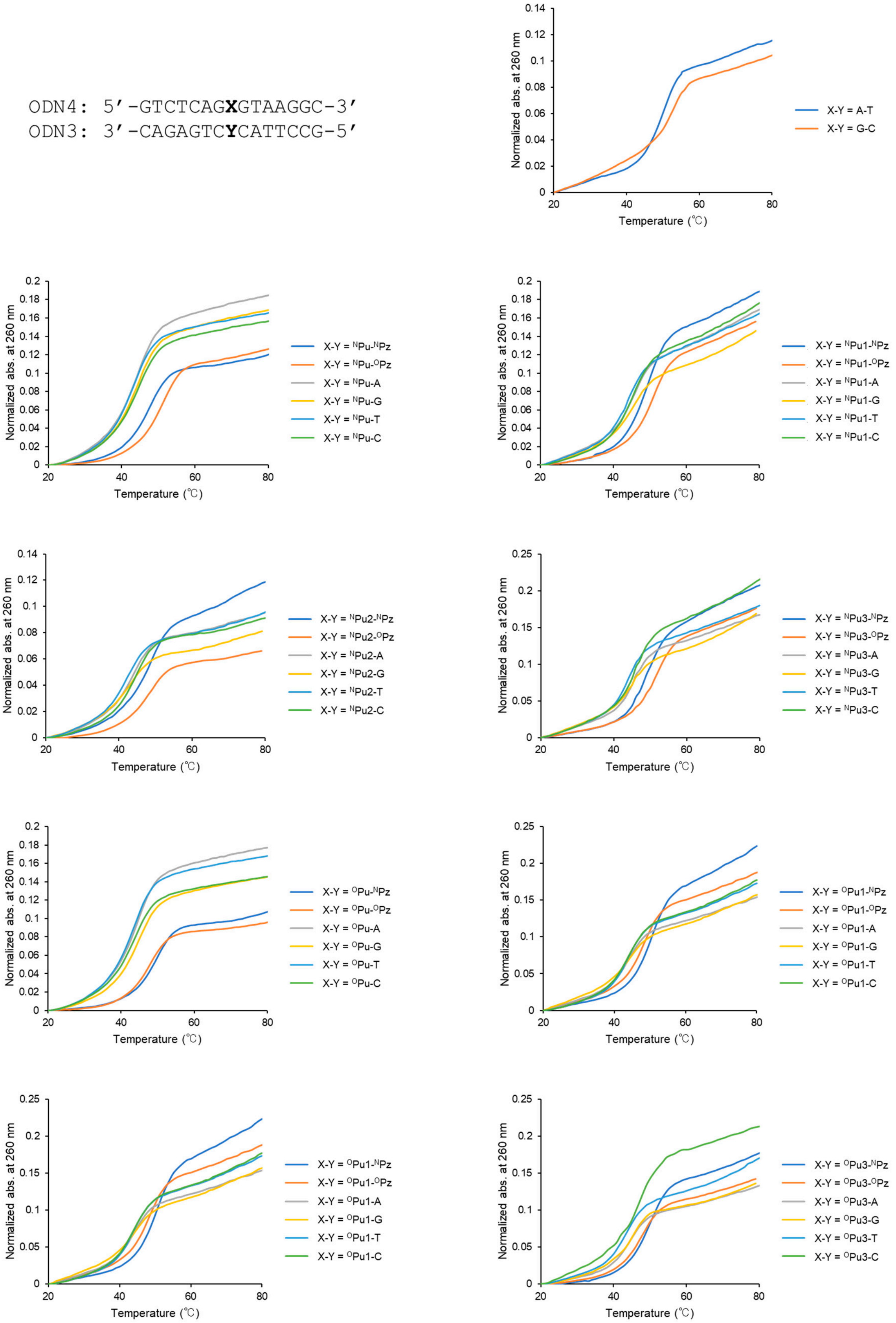

Figure S2 (continued). UV melting curves of the duplex DNAs incorporating different combinations of X-Y base pairs.

Table S1. T_m values of the duplex DNAs incorporating canonical as well as ^{N}Pu and ^{O}Pu derivatives at X-Y position in an inverted manner.

```
ODN4:  5'-GTCTCAG X GTAAGGC-3'
ODN3:  3'-CAGAGTC Y CATTCCG-5'
```

Base pair	T_m (˚C)	Base pair	T_m (˚C)
**A - T**	49.9 ± 0.1	**G - C**	52.4 ± 0.1
**^{N}Pu - ^{O}Pz**	51.2 ± 0.1	**^{N}Pu - ^{N}Pz**	47.5 ± 0.1
**^{N}Pu1 - ^{O}Pz**	51.0 ± 0.2	**^{N}Pu1 - ^{N}Pz**	49.2 ± 0.2
**^{N}Pu2 - ^{O}Pz**	48.4 ± 0.1	**^{N}Pu2 - ^{N}Pz**	48.6 ± 0.1
**^{N}Pu3 - ^{O}Pz**	52.3 ± 0.2	**^{N}Pu3 - ^{N}Pz**	49.7 ± 0.3
**^{O}Pu - ^{N}Pz**	49.6 ± 0.1	**^{O}Pu - ^{O}Pz**	47.7 ± 0.1
**^{O}Pu1 - ^{N}Pz**	50.7 ± 0.3	**^{O}Pu1 - ^{O}Pz**	47.9 ± 0.7
**^{O}Pu2 - ^{N}Pz**	47.7 ± 0.3	**^{O}Pu2 - ^{O}Pz**	47.9 ± 0.7
**^{O}Pu3 - ^{N}Pz**	49.9 ± 0.1	**^{O}Pu3 - ^{O}Pz**	47.9 ± 0.1

[a]Conditions: 2 μM duplex DNA, 10 mM sodium phosphate, 50 mM NaCl, pH 7.0.

Table S2. T_m values of the duplex DNAs incorporating the canonical nucleobases against ^{N}Pu and ^{O}Pu derivatives.

```
ODN3: 5'- GCCTTAC X CTGAGAC -3'
ODN4: 3'- CGGAATG Y GACTCTG -5'
```

Base pair (X-Y)	T_m (˚C)	Base pair (X-Y)	T_m (˚C)
**^{N}Pu - A**	44.6 ± 0.1	**^{O}Pu - A**	44.4 ± 0.1
**^{N}Pu - G**	46.6 ± 0.1	**^{O}Pu - G**	46.9 ± 0.1
**^{N}Pu - T**	44.6 ± 0.1	**^{O}Pu - T**	45.0 ± 0.1
**^{N}Pu - C**	43.5 ± 0.1	**^{O}Pu - C**	43.6 ± 0.1
**^{N}Pu1 - A**	46.5 ± 0.1	**^{O}Pu1 - A**	46.9 ± 0.2
**^{N}Pu1 - G**	48.2 ± 0.1	**^{O}Pu1 - G**	47.3 ± 0.3
**^{N}Pu1 - T**	45.8 ± 0.1	**^{O}Pu1 - T**	47.9 ± 0.5
**^{N}Pu1 - C**	45.0 ± 0.1	**^{O}Pu1 - C**	47.8 ± 0.2
**^{N}Pu2 - A**	44.5 ± 0.1	**^{O}Pu2 - A**	45.6 ± 0.1
**^{N}Pu2 - G**	45.5 ± 0.1	**^{O}Pu2 - G**	46.8 ± 0.2
**^{N}Pu2 - T**	44.7 ± 0.2	**^{O}Pu2 - T**	44.7 ± 0.1
**^{N}Pu2 - C**	45.1 ± 0.1	**^{O}Pu2 - C**	45.3 ± 0.1
**^{N}Pu3 - A**	46.5 ± 0.1	**^{O}Pu3 - A**	45.1 ± 0.1
**^{N}Pu3 - G**	47.7 ± 0.1	**^{O}Pu3 - G**	47.0 ± 0.1
**^{N}Pu3 - T**	46.0 ± 0.1	**^{O}Pu3 - T**	45.6 ± 0.1
**^{N}Pu3 - C**	47.0 ± 0.4	**^{O}Pu3 - C**	45.8 ± 0.2

[a]Conditions: 2 μM duplex DNA, 10 mM sodium phosphate, 50 mM NaCl, pH 7.0.

Table S3. T_m values of the duplex DNAs incorporating the canonical nucleobases against ^{N}Pu and ^{O}Pu derivatives at X-Y position in an inverted manner.

```
ODN4: 5'-GTCTCAG X GTAAGGC-3'
ODN3: 3'-CAGAGTC Y CATTCCG-5'
```

Base pair (X-Y)	T_m (˚C)	Base pair (X-Y)	T_m (˚C)
**^{N}Pu - A**	43.9 ± 0.1	**^{O}Pu - A**	44.4 ± 0.1
**^{N}Pu - G**	44.2 ± 0.1	**^{O}Pu - G**	44.8 ± 0.1
**^{N}Pu - T**	43.2 ± 0.2	**^{O}Pu - T**	43.5 ± 0.1
**^{N}Pu - C**	44.5 ± 0.1	**^{O}Pu - C**	43.5 ± 0.1
**^{N}Pu1 - A**	45.6 ± 0.1	**^{O}Pu1 - A**	44.3 ± 0.3
**^{N}Pu1 - G**	45.4 ± 0.1	**^{O}Pu1 - G**	44.4 ± 0.3
**^{N}Pu1 - T**	44.0 ± 0.1	**^{O}Pu1 - T**	44.1 ± 0.3
**^{N}Pu1 - C**	45.3 ± 0.1	**^{O}Pu1 - C**	44.1 ± 0.3
**^{N}Pu2 - A**	43.5 ± 0.4	**^{O}Pu2 - A**	47.0 ± 0.2
**^{N}Pu2 - G**	42.7 ± 0.1	**^{O}Pu2 - G**	44.4 ± 0.3
**^{N}Pu2 - T**	42.1 ± 0.1	**^{O}Pu2 - T**	44.0 ± 0.2
**^{N}Pu2 - C**	44.3 ± 0.1	**^{O}Pu2 - C**	45.1 ± 0.2
**^{N}Pu3 - A**	46.0 ± 0.5	**^{O}Pu3 - A**	44.5 ± 0.2
**^{N}Pu3 - G**	45.8 ± 0.2	**^{O}Pu3 - G**	44.9 ± 0.5
**^{N}Pu3 - T**	44.6 ± 0.2	**^{O}Pu3 - T**	43.4 ± 0.3
**^{N}Pu3 - C**	47.2 ± 0.1	**^{O}Pu3 - C**	45.2 ± 0.2

[a]Conditions: 2 µM duplex DNA, 10 mM sodium phosphate, 50 mM NaCl, pH 7.0.

3. Speculated recognition modes of the alkynylated purine derivatives

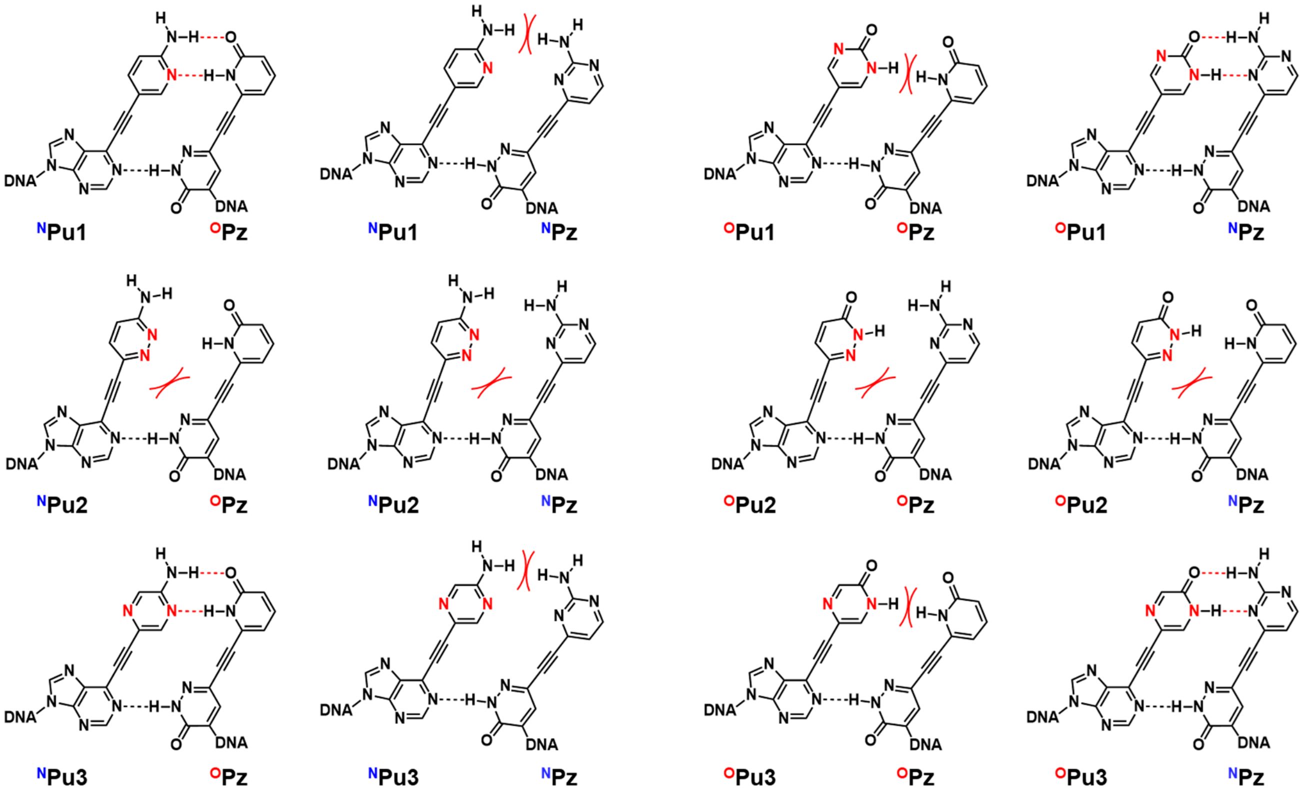

Figure S3. Speculated recognition modes of the alkynylated purine derivatives pairing with the alkynylated pyridazines.

4. ^{1}H, ^{13}C and ^{31}P NMR spectra of the synthesized compounds

Compound 2

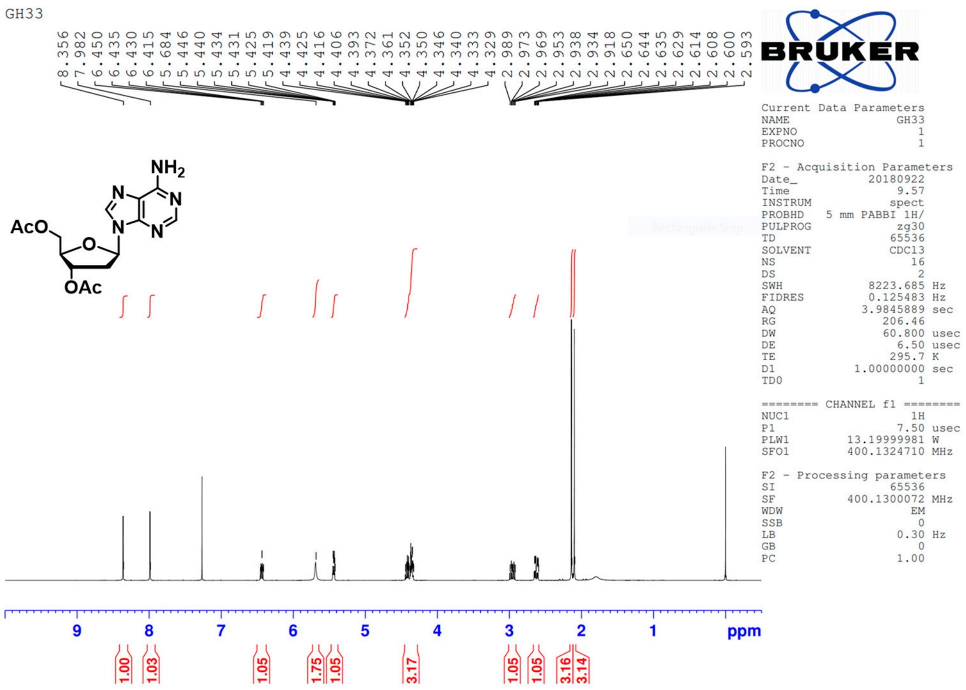

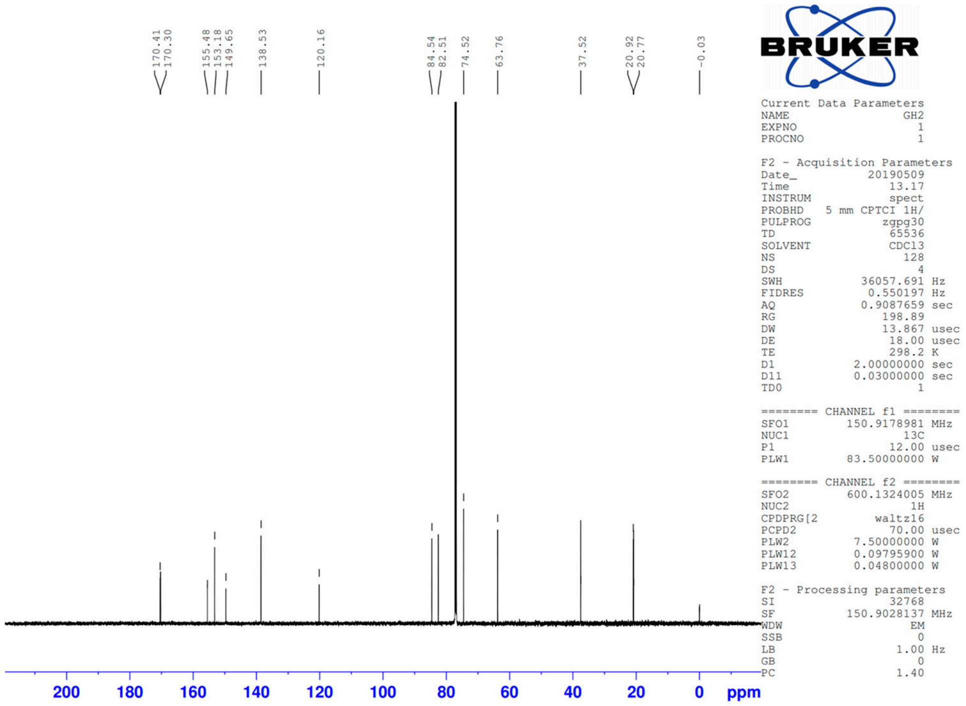

Compound 3

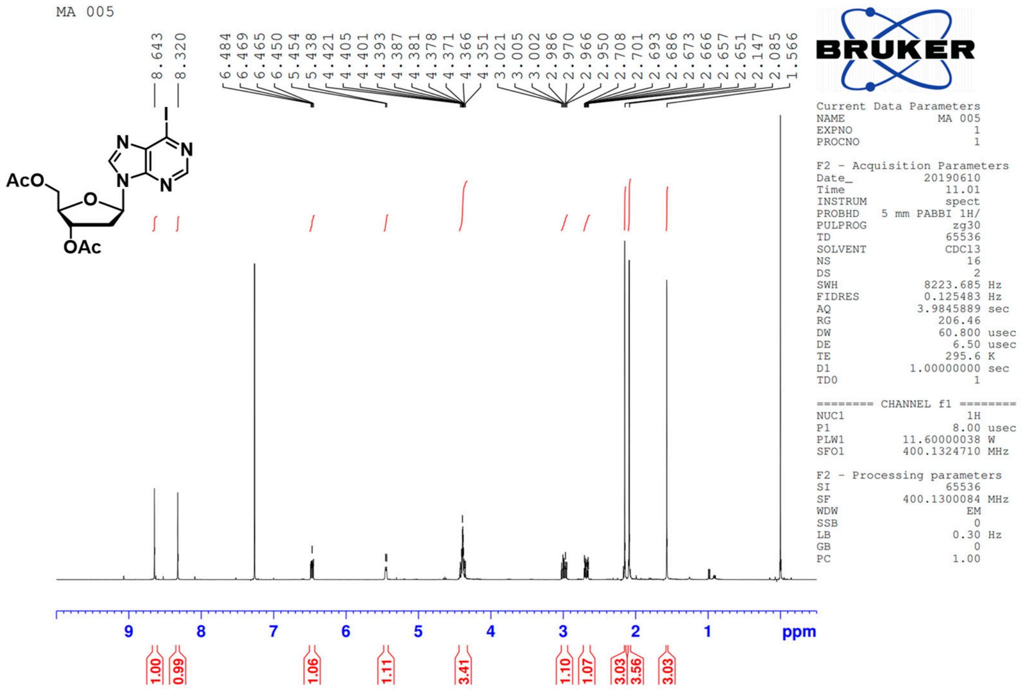
MA 005
8.643
8.320
6.484
6.469
6.465
6.450
5.454
5.438
4.421
4.405
4.401
4.393
4.387
4.381
4.378
4.371
4.366
4.351
3.021
3.005
3.002
2.986
2.970
2.966
2.950
2.708
2.701
2.693
2.686
2.673
2.666
2.657
2.651
2.147
2.085
1.566
AcO
OAc
N
N
N
N
O
I
Current Data Parameters
NAME MA 005
EXPNO 1
PROCNO 1
F2 - Acquisition Parameters
Date_ 20190610
Time 11.01
INSTRUM spect
PROBHD 5 mm PABBI 1H/
PULPROG zg30
TD 65536
SOLVENT CDCl3
NS 16
DS 2
SWH 8223.685 Hz
FIDRES 0.125483 Hz
AQ 3.9845889 sec
RG 206.46
DW 60.800 usec
DE 6.50 usec
TE 295.6 K
D1 1.00000000 sec
TD0 1
======== CHANNEL f1 ========
NUC1 1H
P1 8.00 usec
PLW1 11.60000038 W
SFO1 400.1324710 MHz
F2 - Processing parameters
SI 65536
SF 400.1300084 MHz
WDW EM
SSB 0
LB 0.30 Hz
GB 0
PC 1.00
9
8
7
6
5
4
3
2
1
ppm
1.00
0.99
1.06
1.11
3.41
1.10
1.07
3.03
3.56
3.03

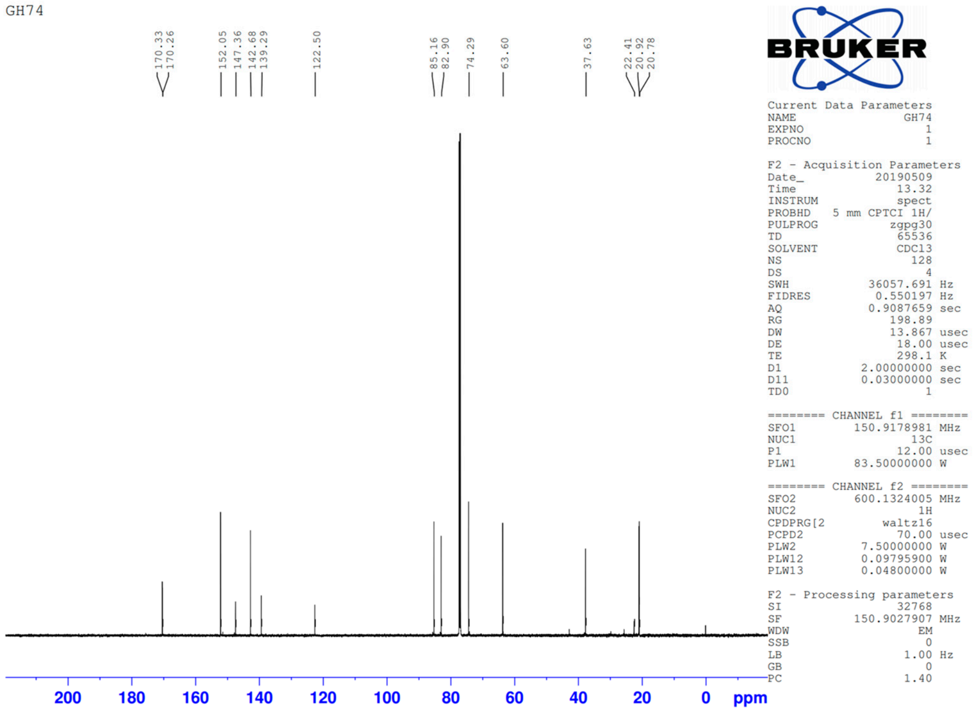
GH74
170.33
170.26
152.05
147.36
142.68
139.29
122.50
85.16
82.90
74.29
63.60
37.63
22.41
20.92
20.78
Current Data Parameters
NAME GH74
EXPNO 1
PROCNO 1
F2 - Acquisition Parameters
Date_ 20190509
Time 13.32
INSTRUM spect
PROBHD 5 mm CPTCI 1H/
PULPROG zgpg30
TD 65536
SOLVENT CDCl3
NS 128
DS 4
SWH 36057.691 Hz
FIDRES 0.550197 Hz
AQ 0.9087659 sec
RG 198.89
DW 13.867 usec
DE 18.00 usec
TE 298.1 K
D1 2.00000000 sec
D11 0.03000000 sec
TD0 1
======== CHANNEL f1 ========
SFO1 150.9178981 MHz
NUC1 13C
P1 12.00 usec
PLW1 83.50000000 W
======== CHANNEL f2 ========
SFO2 600.1324005 MHz
NUC2 1H
CPDPRG[2 waltz16
PCPD2 70.00 usec
PLW2 7.50000000 W
PLW12 0.09795900 W
PLW13 0.04800000 W
F2 - Processing parameters
SI 32768
SF 150.9027907 MHz
WDW EM
SSB 0
LB 1.00 Hz
GB 0
PC 1.40
200
180
160
140
120
100
80
60
40
20
0
ppm

Compound 4

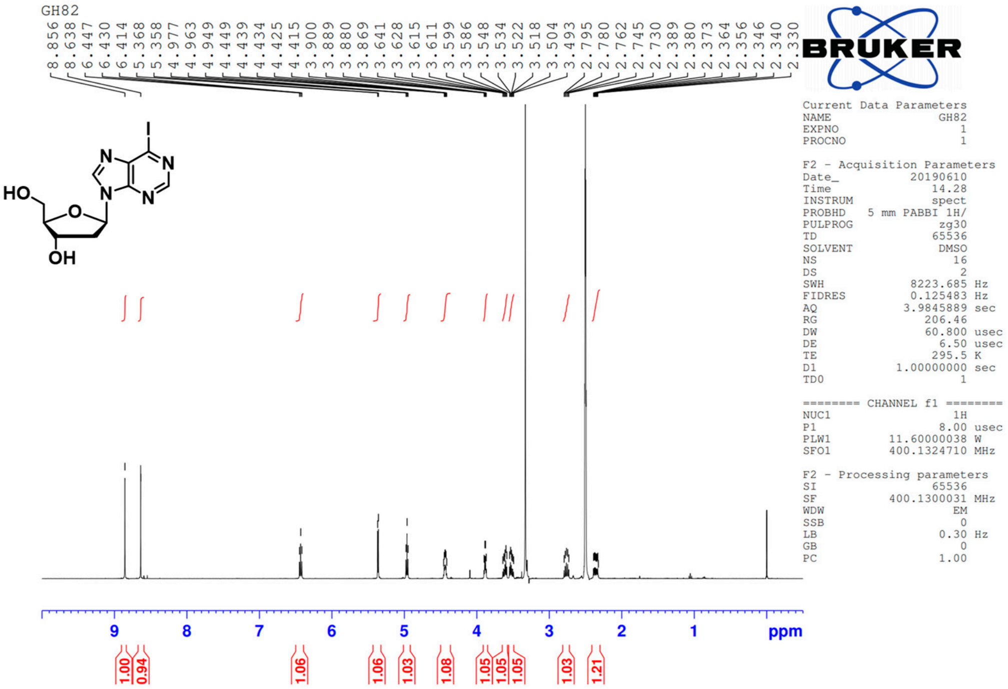
GH82
8.856 8.638 6.447 6.430 6.414 5.368 5.358 4.977 4.963 4.949 4.449 4.439 4.434 4.425 4.415 3.900 3.889 3.880 3.869 3.641 3.628 3.615 3.611 3.599 3.586 3.548 3.534 3.522 3.518 3.504 3.493 2.795 2.780 2.762 2.745 2.730 2.389 2.380 2.373 2.364 2.356 2.346 2.340 2.330
HO
OH
N
I
BRUKER
Current Data Parameters
NAME GH82
EXPNO 1
PROCNO 1
F2 - Acquisition Parameters
Date_ 20190610
Time 14.28
INSTRUM spect
PROBHD 5 mm PABBI 1H/
PULPROG zg30
TD 65536
SOLVENT DMSO
NS 16
DS 2
SWH 8223.685 Hz
FIDRES 0.125483 Hz
AQ 3.9845889 sec
RG 206.46
DW 60.800 usec
DE 6.50 usec
TE 295.5 K
D1 1.00000000 sec
TD0 1
======== CHANNEL f1 ========
NUC1 1H
P1 8.00 usec
PLW1 11.60000038 W
SFO1 400.1324710 MHz
F2 - Processing parameters
SI 65536
SF 400.1300031 MHz
WDW EM
SSB 0
LB 0.30 Hz
GB 0
PC 1.00
9 8 7 6 5 4 3 2 1 ppm
1.00 0.94 1.06 1.06 1.03 1.08 1.05 1.05 1.05 1.03 1.21

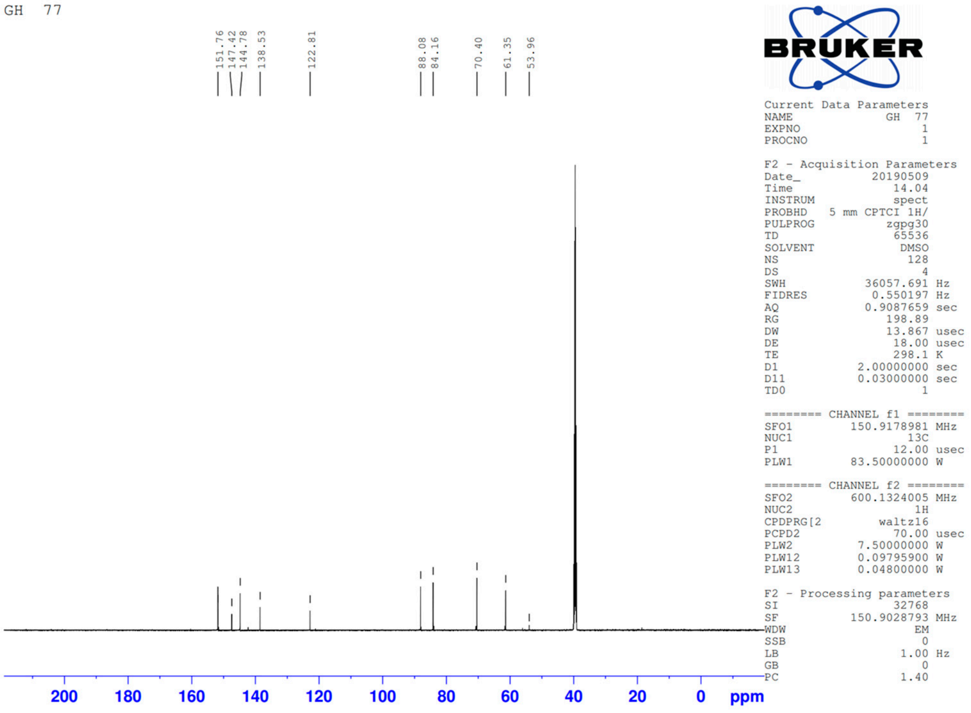
GH 77
151.76 147.42 144.78 138.53 122.81 88.08 84.16 70.40 61.35 53.96
BRUKER
Current Data Parameters
NAME GH 77
EXPNO 1
PROCNO 1
F2 - Acquisition Parameters
Date_ 20190509
Time 14.04
INSTRUM spect
PROBHD 5 mm CPTCI 1H/
PULPROG zgpg30
TD 65536
SOLVENT DMSO
NS 128
DS 4
SWH 36057.691 Hz
FIDRES 0.550197 Hz
AQ 0.9087659 sec
RG 198.89
DW 13.867 usec
DE 18.00 usec
TE 298.1 K
D1 2.00000000 sec
D11 0.03000000 sec
TD0 1
======== CHANNEL f1 ========
SFO1 150.9178981 MHz
NUC1 13C
P1 12.00 usec
PLW1 83.50000000 W
======== CHANNEL f2 ========
SFO2 600.1324005 MHz
NUC2 1H
CPDPRG[2 waltz16
PCPD2 70.00 usec
PLW2 7.50000000 W
PLW12 0.09795900 W
PLW13 0.04800000 W
F2 - Processing parameters
SI 32768
SF 150.9028793 MHz
WDW EM
SSB 0
LB 1.00 Hz
GB 0
PC 1.40
200 180 160 140 120 100 80 60 40 20 0 ppm

Compound 5

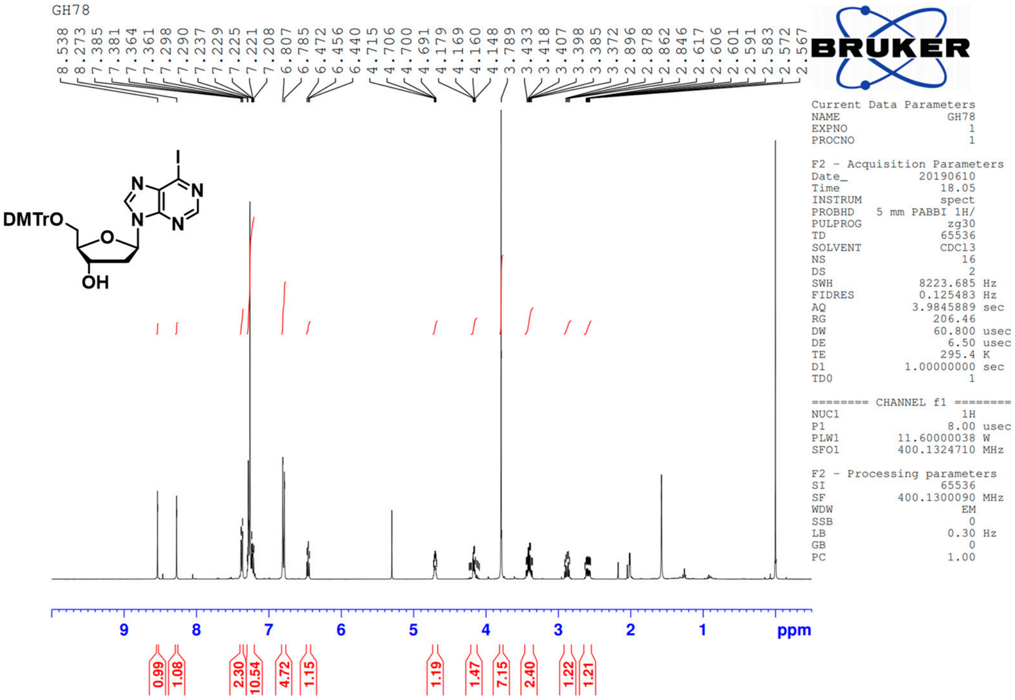
GH78
8.538 8.273 7.385 7.381 7.364 7.361 7.298 7.290 7.237 7.229 7.225 7.221 7.208 6.807 6.785 6.472 6.456 6.440 4.715 4.706 4.700 4.691 4.179 4.169 4.160 4.148 3.789 3.433 3.418 3.407 3.398 3.385 3.372 2.896 2.878 2.862 2.846 2.617 2.606 2.601 2.591 2.583 2.572 2.567
I
N
DMTrO
O
OH
Current Data Parameters
NAME GH78
EXPNO 1
PROCNO 1
F2 - Acquisition Parameters
Date_ 20190610
Time 18.05
INSTRUM spect
PROBHD 5 mm PABBI 1H/
PULPROG zg30
TD 65536
SOLVENT CDCl3
NS 16
DS 2
SWH 8223.685 Hz
FIDRES 0.125483 Hz
AQ 3.9845889 sec
RG 206.46
DW 60.800 usec
DE 6.50 usec
TE 295.4 K
D1 1.00000000 sec
TD0 1
======== CHANNEL f1 ========
NUC1 1H
P1 8.00 usec
PLW1 11.60000038 W
SFO1 400.1324710 MHz
F2 - Processing parameters
SI 65536
SF 400.1300090 MHz
WDW EM
SSB 0
LB 0.30 Hz
GB 0
PC 1.00
9 8 7 6 5 4 3 2 1 ppm
0.99 1.08 2.30 10.54 4.72 1.15 1.19 1.47 7.15 2.40 1.22 1.21

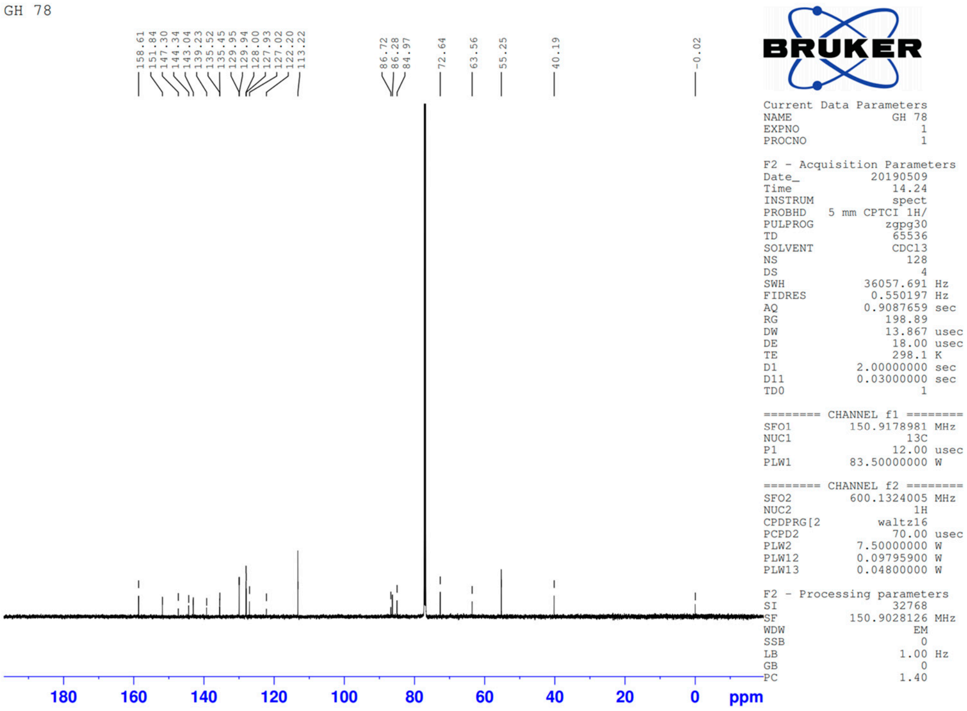
GH 78
158.61 151.84 147.30 144.34 143.04 139.23 135.52 135.45 129.95 129.94 128.00 127.93 127.02 122.20 113.22 86.72 86.28 84.97 72.64 63.56 55.25 40.19 -0.02
Current Data Parameters
NAME GH 78
EXPNO 1
PROCNO 1
F2 - Acquisition Parameters
Date_ 20190509
Time 14.24
INSTRUM spect
PROBHD 5 mm CPTCI 1H/
PULPROG zgpg30
TD 65536
SOLVENT CDCl3
NS 128
DS 4
SWH 36057.691 Hz
FIDRES 0.550197 Hz
AQ 0.9087659 sec
RG 198.89
DW 13.867 usec
DE 18.00 usec
TE 298.1 K
D1 2.00000000 sec
D11 0.03000000 sec
TD0 1
======== CHANNEL f1 ========
SFO1 150.9178981 MHz
NUC1 13C
P1 12.00 usec
PLW1 83.50000000 W
======== CHANNEL f2 ========
SFO2 600.1324005 MHz
NUC2 1H
CPDPRG[2 waltz16
PCPD2 70.00 usec
PLW2 7.50000000 W
PLW12 0.09795900 W
PLW13 0.04800000 W
F2 - Processing parameters
SI 32768
SF 150.9028126 MHz
WDW EM
SSB 0
LB 1.00 Hz
GB 0
PC 1.40
180 160 140 120 100 80 60 40 20 0 ppm

Compound 6

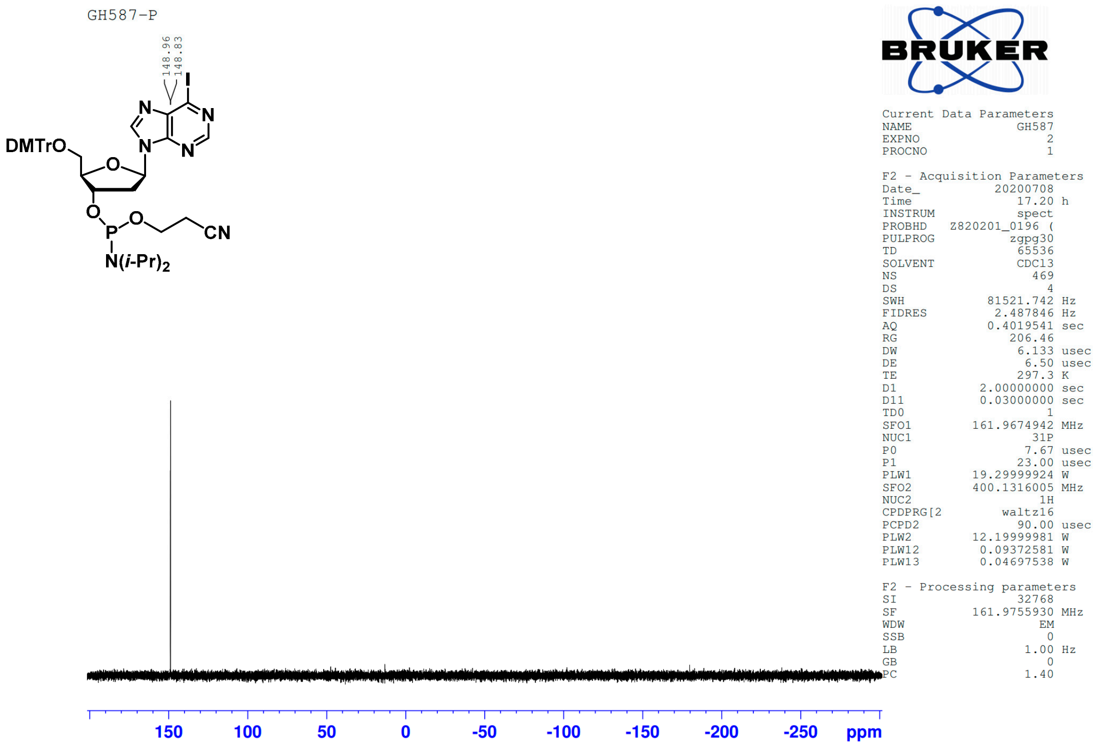
GH587-P
148.96
148.83
DMTrO
N(i-Pr)2
CN
Current Data Parameters
NAME GH587
EXPNO 2
PROCNO 1
F2 - Acquisition Parameters
Date_ 20200708
Time 17.20 h
INSTRUM spect
PROBHD Z820201_0196 (
PULPROG zgpg30
TD 65536
SOLVENT CDCl3
NS 469
DS 4
SWH 81521.742 Hz
FIDRES 2.487846 Hz
AQ 0.4019541 sec
RG 206.46
DW 6.133 usec
DE 6.50 usec
TE 297.3 K
D1 2.00000000 sec
D11 0.03000000 sec
TD0 1
SFO1 161.9674942 MHz
NUC1 31P
P0 7.67 usec
P1 23.00 usec
PLW1 19.29999924 W
SFO2 400.1316005 MHz
NUC2 1H
CPDPRG[2 waltz16
PCPD2 90.00 usec
PLW2 12.19999981 W
PLW12 0.09372581 W
PLW13 0.04697538 W
F2 - Processing parameters
SI 32768
SF 161.9755930 MHz
WDW EM
SSB 0
LB 1.00 Hz
GB 0
PC 1.40
150
100
50
0
-50
-100
-150
-200
-250
ppm

Compound 8

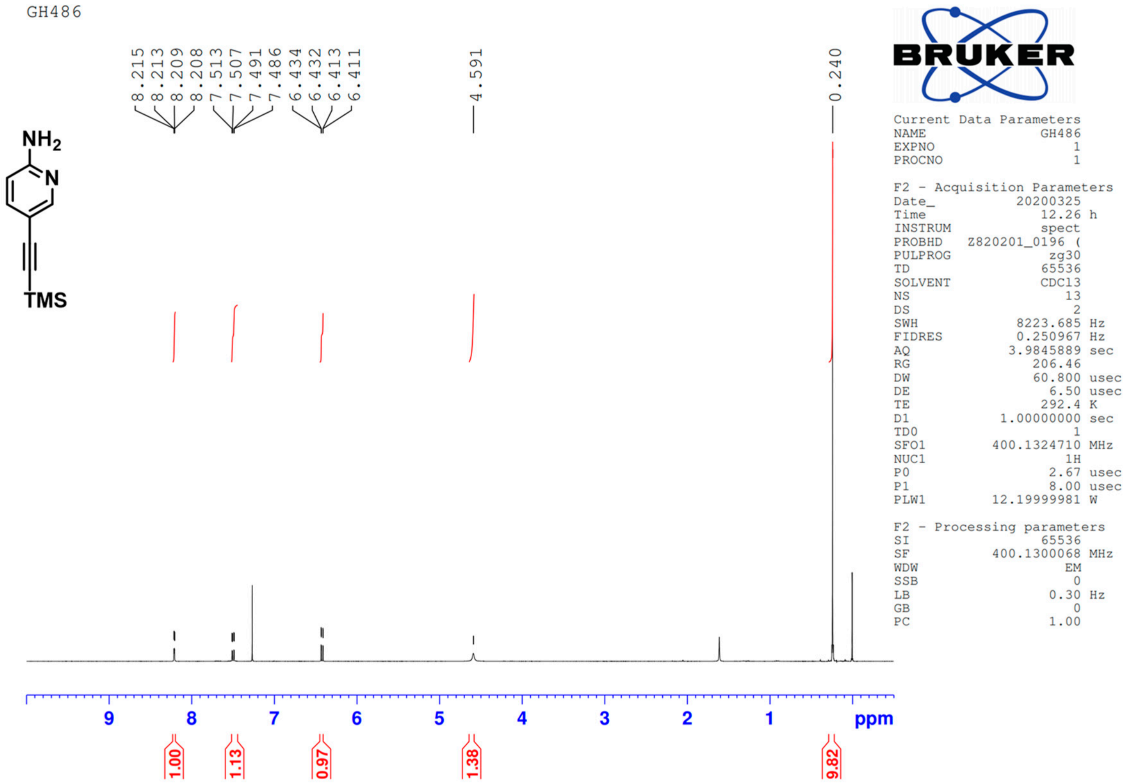
GH486
8.215
8.213
8.209
8.208
7.513
7.507
7.491
7.486
6.434
6.432
6.413
6.411
4.591
0.240
NH2
N
TMS
BRUKER
Current Data Parameters
NAME GH486
EXPNO 1
PROCNO 1
F2 - Acquisition Parameters
Date_ 20200325
Time 12.26 h
INSTRUM spect
PROBHD Z820201_0196 (
PULPROG zg30
TD 65536
SOLVENT CDCl3
NS 13
DS 2
SWH 8223.685 Hz
FIDRES 0.250967 Hz
AQ 3.9845889 sec
RG 206.46
DW 60.800 usec
DE 6.50 usec
TE 292.4 K
D1 1.00000000 sec
TD0 1
SFO1 400.1324710 MHz
NUC1 1H
P0 2.67 usec
P1 8.00 usec
PLW1 12.19999981 W
F2 - Processing parameters
SI 65536
SF 400.1300068 MHz
WDW EM
SSB 0
LB 0.30 Hz
GB 0
PC 1.00
9
8
7
6
5
4
3
2
1
ppm
1.00
1.13
0.97
1.38
9.82

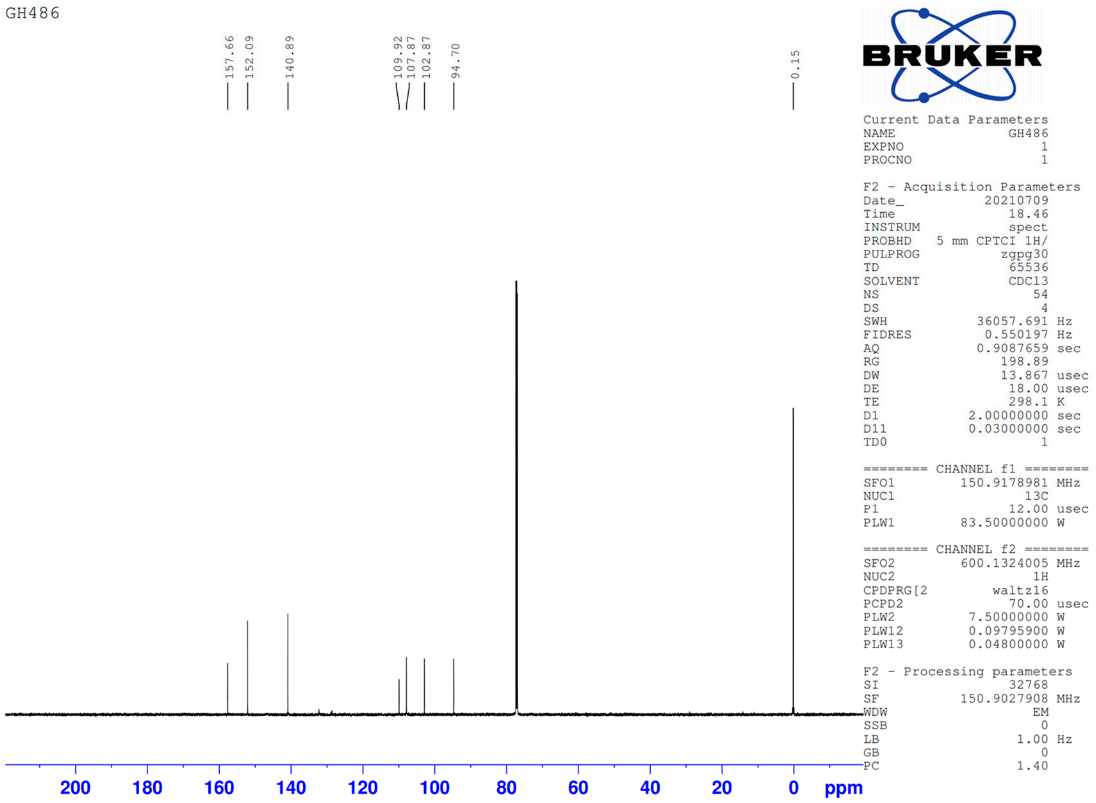
GH486
157.66
152.09
140.89
109.92
107.87
102.87
94.70
0.15
BRUKER
Current Data Parameters
NAME GH486
EXPNO 1
PROCNO 1
F2 - Acquisition Parameters
Date_ 20210709
Time 18.46
INSTRUM spect
PROBHD 5 mm CPTCI 1H/
PULPROG zgpg30
TD 65536
SOLVENT CDCl3
NS 54
DS 4
SWH 36057.691 Hz
FIDRES 0.550197 Hz
AQ 0.9087659 sec
RG 198.89
DW 13.867 usec
DE 18.00 usec
TE 298.1 K
D1 2.00000000 sec
D11 0.03000000 sec
TD0 1
======== CHANNEL f1 ========
SFO1 150.9178981 MHz
NUC1 13C
P1 12.00 usec
PLW1 83.50000000 W
======== CHANNEL f2 ========
SFO2 600.1324005 MHz
NUC2 1H
CPDPRG[2 waltz16
PCPD2 70.00 usec
PLW2 7.50000000 W
PLW12 0.09795900 W
PLW13 0.04800000 W
F2 - Processing parameters
SI 32768
SF 150.9027908 MHz
WDW EM
SSB 0
LB 1.00 Hz
GB 0
PC 1.40
200
180
160
140
120
100
80
60
40
20
0
ppm

Compound 9

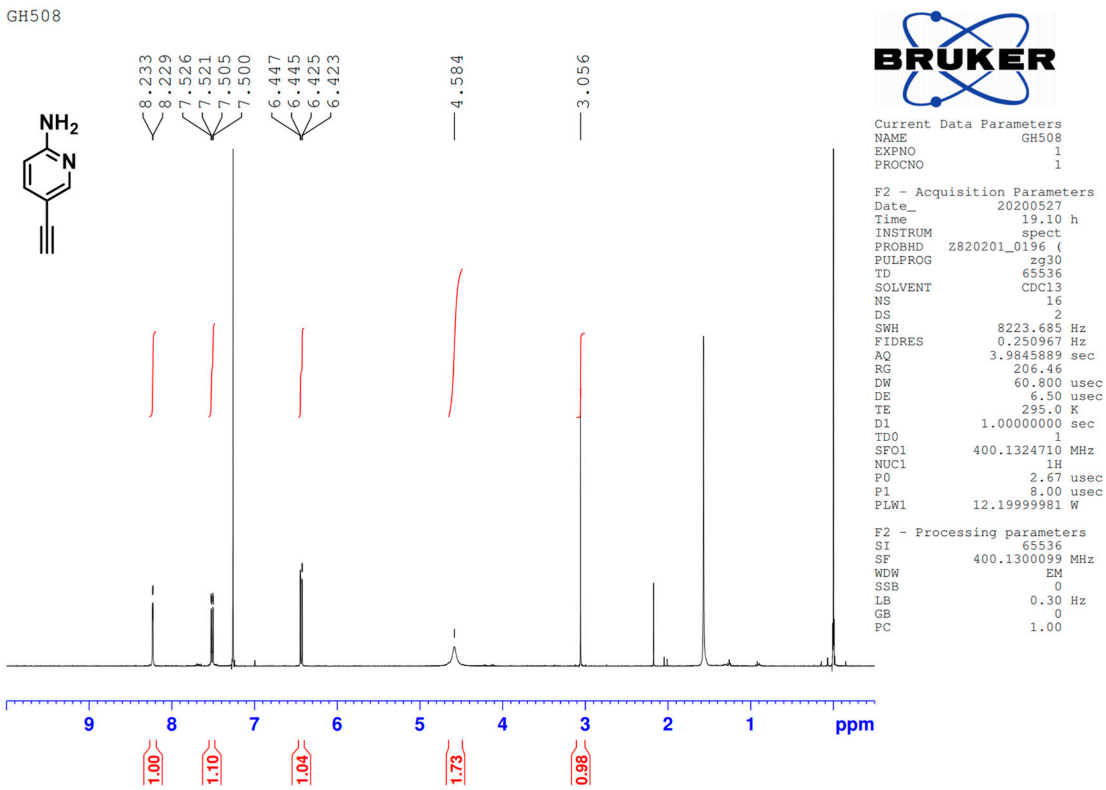

GH508
NH2
N
8.233
8.229
7.526
7.521
7.505
7.500
6.447
6.445
6.425
6.423
4.584
3.056
BRUKER
Current Data Parameters
NAME GH508
EXPNO 1
PROCNO 1
F2 - Acquisition Parameters
Date_ 20200527
Time 19.10 h
INSTRUM spect
PROBHD Z820201_0196 (
PULPROG zg30
TD 65536
SOLVENT CDCl3
NS 16
DS 2
SWH 8223.685 Hz
FIDRES 0.250967 Hz
AQ 3.9845889 sec
RG 206.46
DW 60.800 usec
DE 6.50 usec
TE 295.0 K
D1 1.00000000 sec
TD0 1
SFO1 400.1324710 MHz
NUC1 1H
P0 2.67 usec
P1 8.00 usec
PLW1 12.19999981 W
F2 - Processing parameters
SI 65536
SF 400.1300099 MHz
WDW EM
SSB 0
LB 0.30 Hz
GB 0
PC 1.00
9
8
7
6
5
4
3
2
1
ppm
1.00
1.10
1.04
1.73
0.98

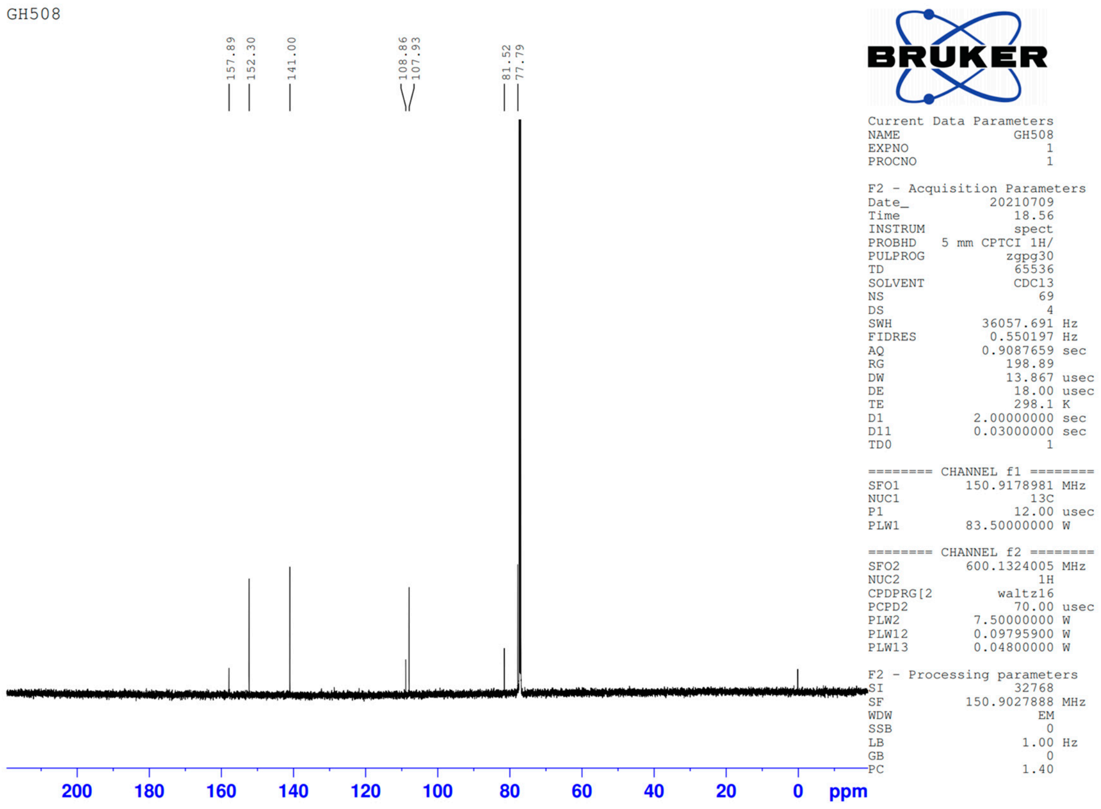

GH508
157.89
152.30
141.00
108.86
107.93
81.52
77.79
BRUKER
Current Data Parameters
NAME GH508
EXPNO 1
PROCNO 1
F2 - Acquisition Parameters
Date_ 20210709
Time 18.56
INSTRUM spect
PROBHD 5 mm CPTCI 1H/
PULPROG zgpg30
TD 65536
SOLVENT CDCl3
NS 69
DS 4
SWH 36057.691 Hz
FIDRES 0.550197 Hz
AQ 0.9087659 sec
RG 198.89
DW 13.867 usec
DE 18.00 usec
TE 298.1 K
D1 2.00000000 sec
D11 0.03000000 sec
TD0 1
======== CHANNEL f1 ========
SFO1 150.9178981 MHz
NUC1 13C
P1 12.00 usec
PLW1 83.50000000 W
======== CHANNEL f2 ========
SFO2 600.1324005 MHz
NUC2 1H
CPDPRG[2 waltz16
PCPD2 70.00 usec
PLW2 7.50000000 W
PLW12 0.09795900 W
PLW13 0.04800000 W
F2 - Processing parameters
SI 32768
SF 150.9027888 MHz
WDW EM
SSB 0
LB 1.00 Hz
GB 0
PC 1.40
200
180
160
140
120
100
80
60
40
20
0
ppm

Compound 11

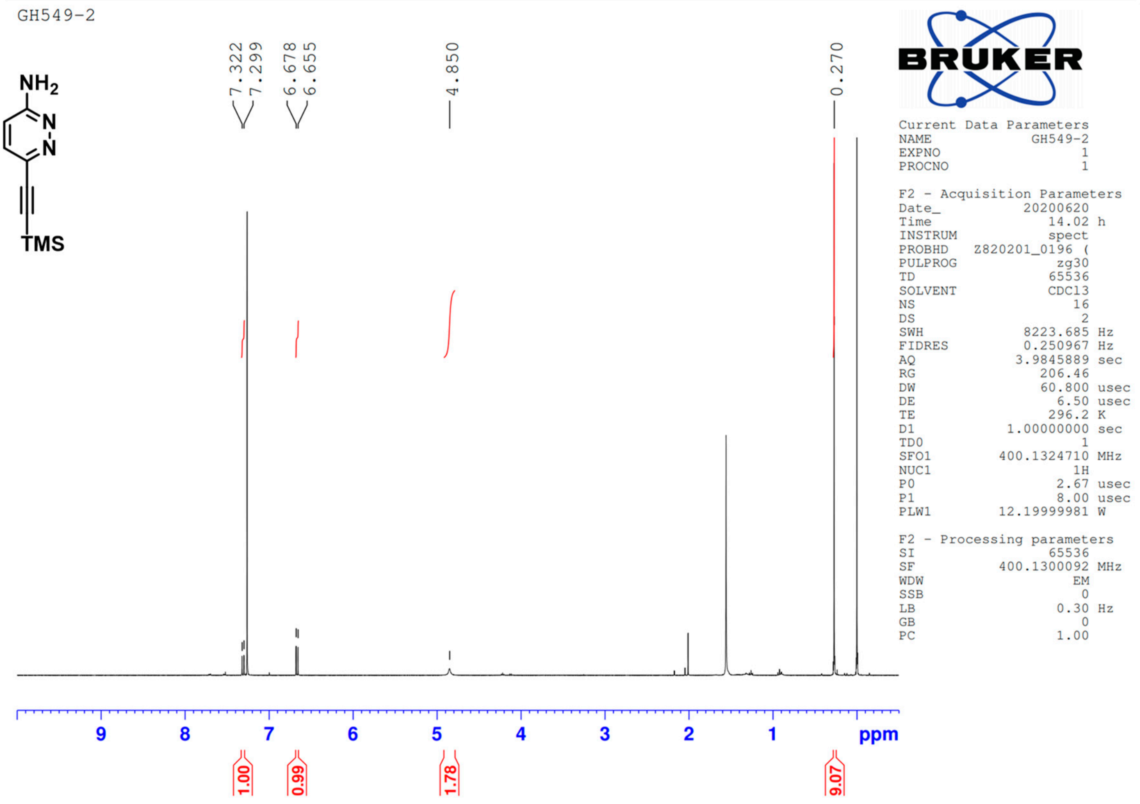
GH549-2
NH2
N
N
TMS
7.322
7.299
6.678
6.655
4.850
0.270
BRUKER
Current Data Parameters
NAME GH549-2
EXPNO 1
PROCNO 1
F2 - Acquisition Parameters
Date_ 20200620
Time 14.02 h
INSTRUM spect
PROBHD Z820201_0196 (
PULPROG zg30
TD 65536
SOLVENT CDCl3
NS 16
DS 2
SWH 8223.685 Hz
FIDRES 0.250967 Hz
AQ 3.9845889 sec
RG 206.46
DW 60.800 usec
DE 6.50 usec
TE 296.2 K
D1 1.00000000 sec
TD0 1
SFO1 400.1324710 MHz
NUC1 1H
P0 2.67 usec
P1 8.00 usec
PLW1 12.19999981 W
F2 - Processing parameters
SI 65536
SF 400.1300092 MHz
WDW EM
SSB 0
LB 0.30 Hz
GB 0
PC 1.00
9
8
7
6
5
4
3
2
1
ppm
1.00
0.99
1.78
9.07

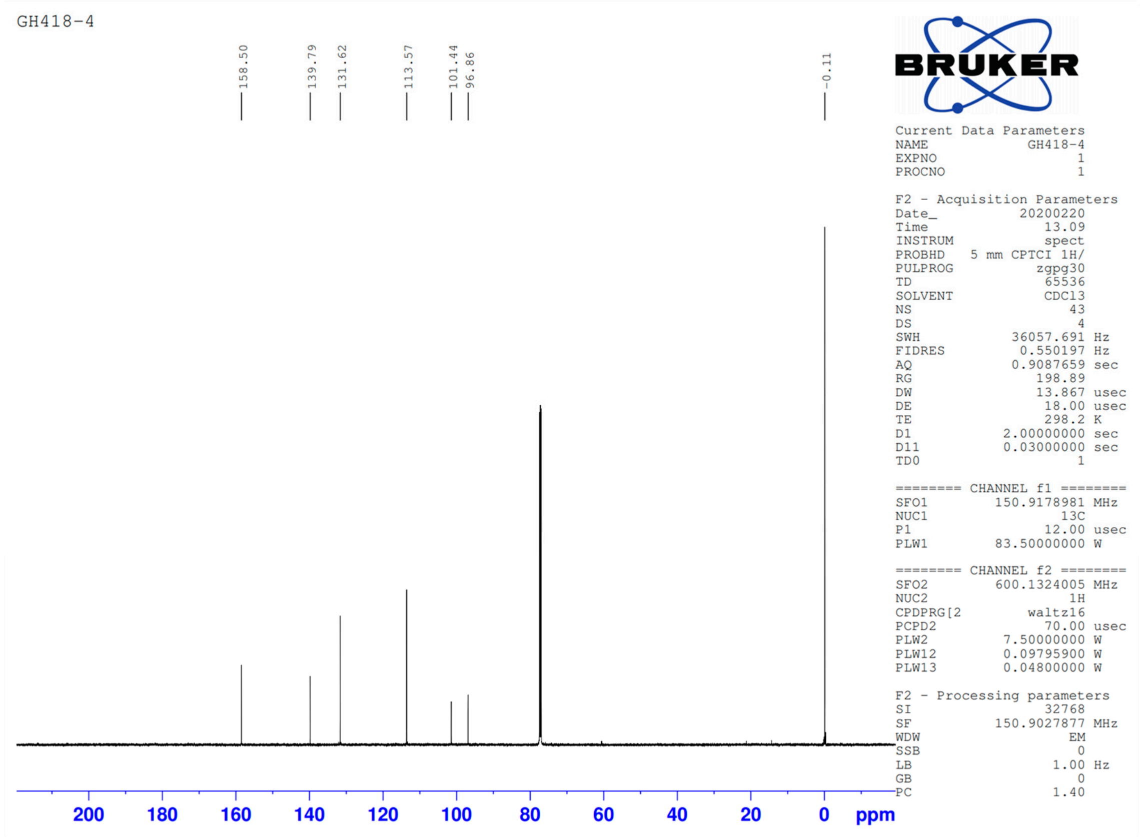
GH418-4
158.50
139.79
131.62
113.57
101.44
96.86
-0.11
BRUKER
Current Data Parameters
NAME GH418-4
EXPNO 1
PROCNO 1
F2 - Acquisition Parameters
Date_ 20200220
Time 13.09
INSTRUM spect
PROBHD 5 mm CPTCI 1H/
PULPROG zgpg30
TD 65536
SOLVENT CDCl3
NS 43
DS 4
SWH 36057.691 Hz
FIDRES 0.550197 Hz
AQ 0.9087659 sec
RG 198.89
DW 13.867 usec
DE 18.00 usec
TE 298.2 K
D1 2.00000000 sec
D11 0.03000000 sec
TD0 1
======== CHANNEL f1 ========
SFO1 150.9178981 MHz
NUC1 13C
P1 12.00 usec
PLW1 83.50000000 W
======== CHANNEL f2 ========
SFO2 600.1324005 MHz
NUC2 1H
CPDPRG[2 waltz16
PCPD2 70.00 usec
PLW2 7.50000000 W
PLW12 0.09795900 W
PLW13 0.04800000 W
F2 - Processing parameters
SI 32768
SF 150.9027877 MHz
WDW EM
SSB 0
LB 1.00 Hz
GB 0
PC 1.40
200
180
160
140
120
100
80
60
40
20
0
ppm

Compound 12

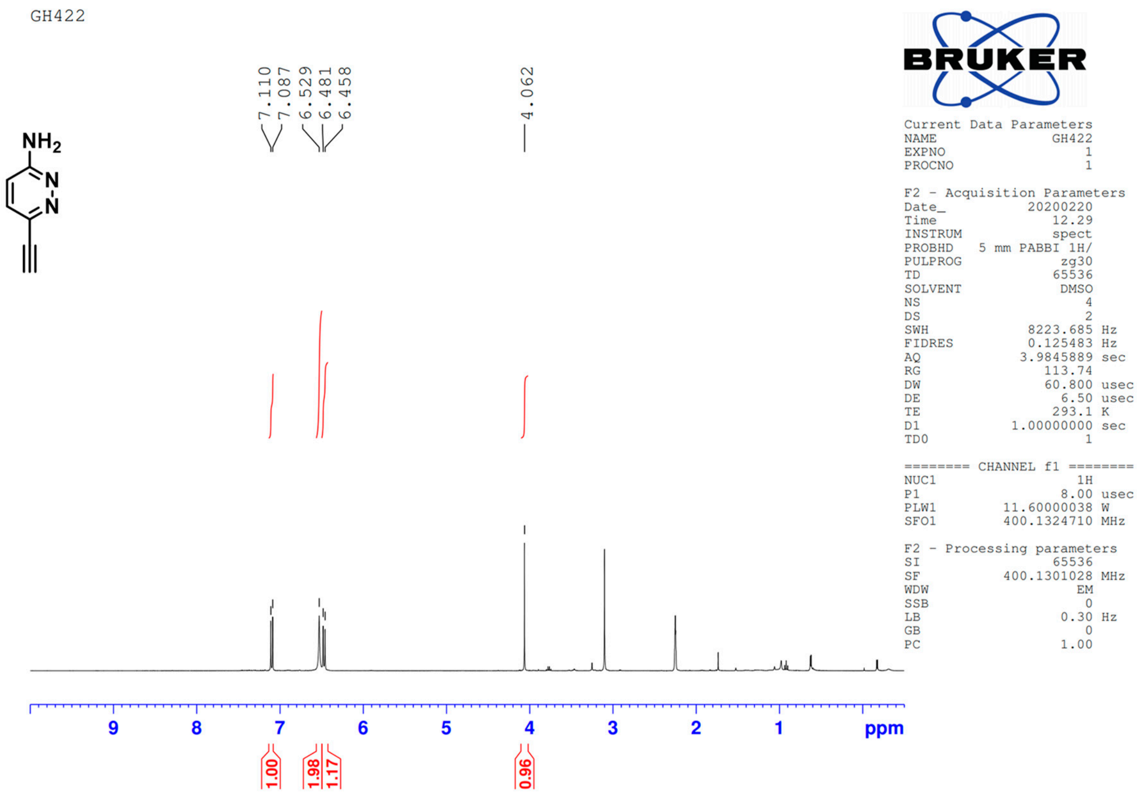
GH422
NH2
N
N
7.110
7.087
6.529
6.481
6.458
4.062
9 8 7 6 5 4 3 2 1 ppm
1.00
1.98
1.17
0.96
BRUKER
Current Data Parameters
NAME GH422
EXPNO 1
PROCNO 1
F2 - Acquisition Parameters
Date_ 20200220
Time 12.29
INSTRUM spect
PROBHD 5 mm PABBI 1H/
PULPROG zg30
TD 65536
SOLVENT DMSO
NS 4
DS 2
SWH 8223.685 Hz
FIDRES 0.125483 Hz
AQ 3.9845889 sec
RG 113.74
DW 60.800 usec
DE 6.50 usec
TE 293.1 K
D1 1.00000000 sec
TD0 1
======== CHANNEL f1 ========
NUC1 1H
P1 8.00 usec
PLW1 11.60000038 W
SFO1 400.1324710 MHz
F2 - Processing parameters
SI 65536
SF 400.1301028 MHz
WDW EM
SSB 0
LB 0.30 Hz
GB 0
PC 1.00

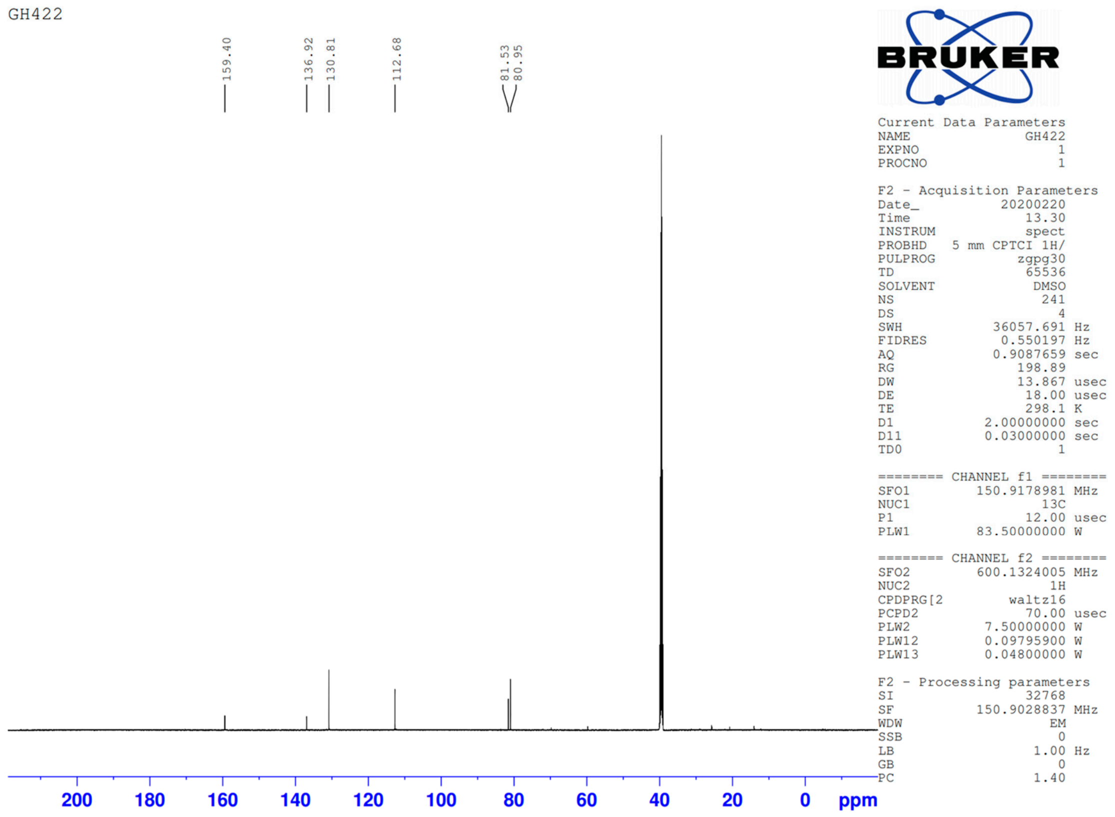
GH422
159.40
136.92
130.81
112.68
81.53
80.95
200 180 160 140 120 100 80 60 40 20 0 ppm
BRUKER
Current Data Parameters
NAME GH422
EXPNO 1
PROCNO 1
F2 - Acquisition Parameters
Date_ 20200220
Time 13.30
INSTRUM spect
PROBHD 5 mm CPTCI 1H/
PULPROG zgpg30
TD 65536
SOLVENT DMSO
NS 241
DS 4
SWH 36057.691 Hz
FIDRES 0.550197 Hz
AQ 0.9087659 sec
RG 198.89
DW 13.867 usec
DE 18.00 usec
TE 298.1 K
D1 2.00000000 sec
D11 0.03000000 sec
TD0 1
======== CHANNEL f1 ========
SFO1 150.9178981 MHz
NUC1 13C
P1 12.00 usec
PLW1 83.50000000 W
======== CHANNEL f2 ========
SFO2 600.1324005 MHz
NUC2 1H
CPDPRG[2 waltz16
PCPD2 70.00 usec
PLW2 7.50000000 W
PLW12 0.09795900 W
PLW13 0.04800000 W
F2 - Processing parameters
SI 32768
SF 150.9028837 MHz
WDW EM
SSB 0
LB 1.00 Hz
GB 0
PC 1.40

Compound 14

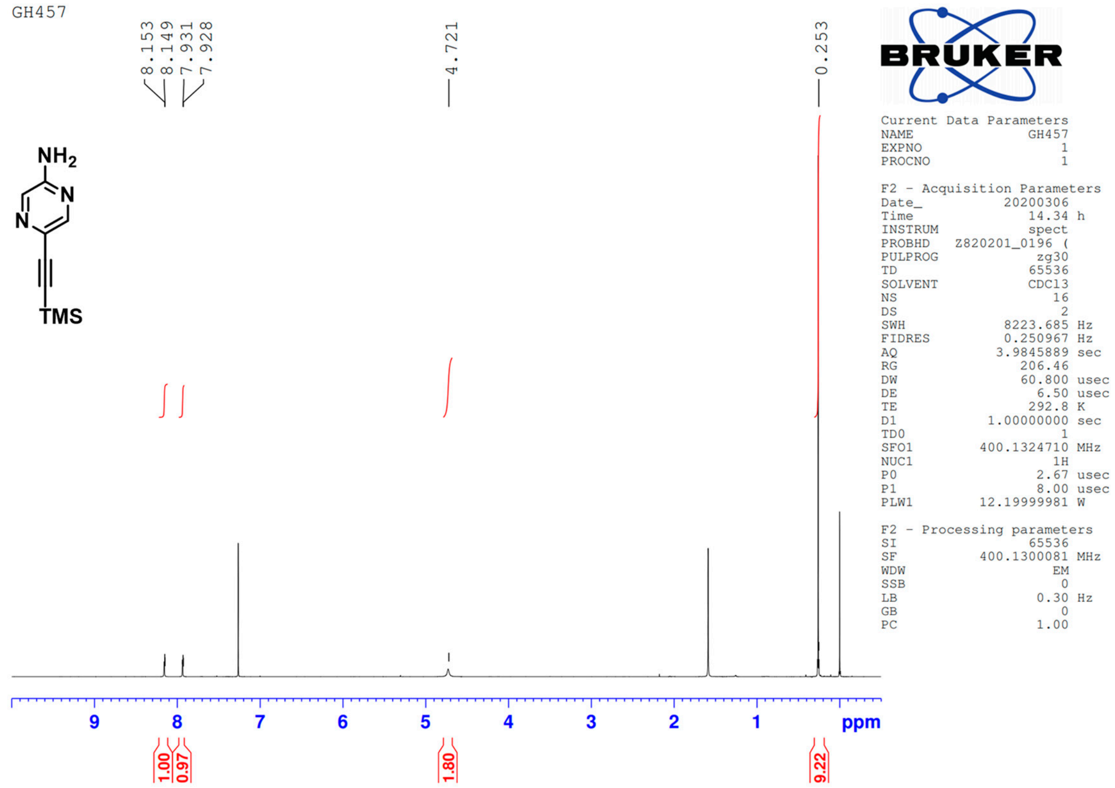
GH457
8.153
8.149
7.931
7.928
4.721
0.253
NH2
N
N
TMS
BRUKER
Current Data Parameters
NAME GH457
EXPNO 1
PROCNO 1
F2 - Acquisition Parameters
Date_ 20200306
Time 14.34 h
INSTRUM spect
PROBHD Z820201_0196 (
PULPROG zg30
TD 65536
SOLVENT CDCl3
NS 16
DS 2
SWH 8223.685 Hz
FIDRES 0.250967 Hz
AQ 3.9845889 sec
RG 206.46
DW 60.800 usec
DE 6.50 usec
TE 292.8 K
D1 1.00000000 sec
TD0 1
SFO1 400.1324710 MHz
NUC1 1H
P0 2.67 usec
P1 8.00 usec
PLW1 12.19999981 W
F2 - Processing parameters
SI 65536
SF 400.1300081 MHz
WDW EM
SSB 0
LB 0.30 Hz
GB 0
PC 1.00
9
8
7
6
5
4
3
2
1
ppm
1.00
0.97
1.80
9.22

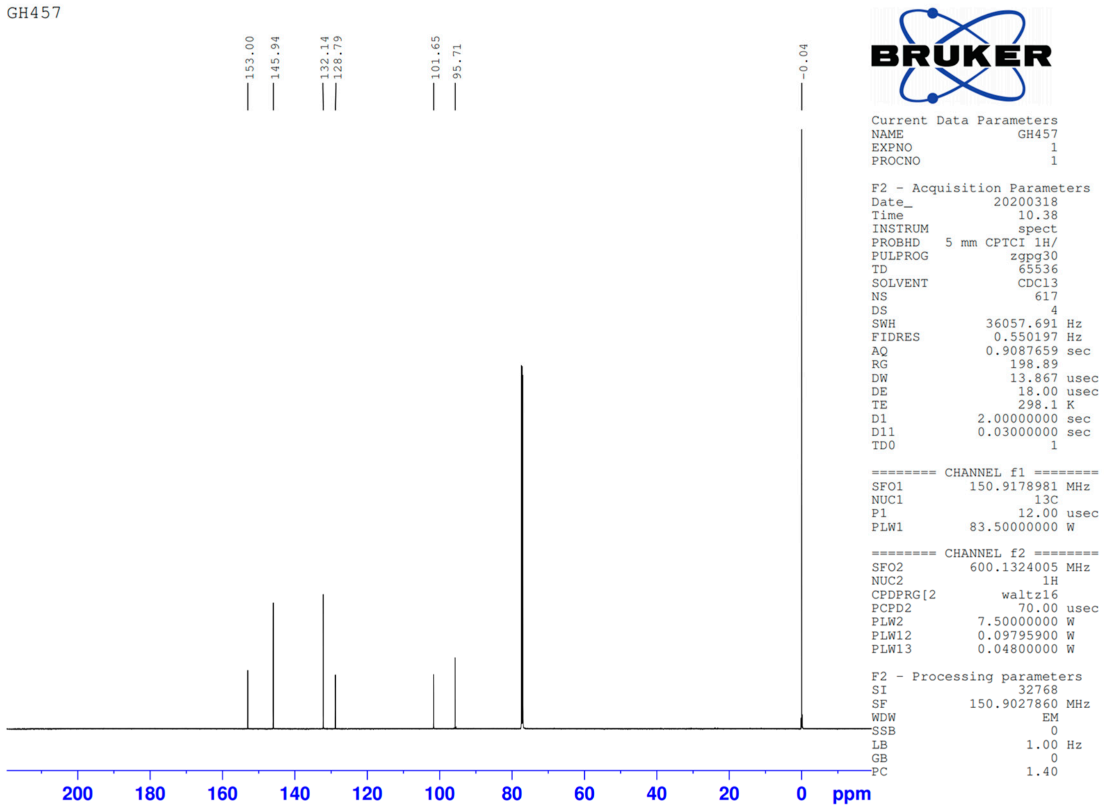
GH457
153.00
145.94
132.14
128.79
101.65
95.71
-0.04
BRUKER
Current Data Parameters
NAME GH457
EXPNO 1
PROCNO 1
F2 - Acquisition Parameters
Date_ 20200318
Time 10.38
INSTRUM spect
PROBHD 5 mm CPTCI 1H/
PULPROG zgpg30
TD 65536
SOLVENT CDCl3
NS 617
DS 4
SWH 36057.691 Hz
FIDRES 0.550197 Hz
AQ 0.9087659 sec
RG 198.89
DW 13.867 usec
DE 18.00 usec
TE 298.1 K
D1 2.00000000 sec
D11 0.03000000 sec
TD0 1
======== CHANNEL f1 ========
SFO1 150.9178981 MHz
NUC1 13C
P1 12.00 usec
PLW1 83.50000000 W
======== CHANNEL f2 ========
SFO2 600.1324005 MHz
NUC2 1H
CPDPRG[2 waltz16
PCPD2 70.00 usec
PLW2 7.50000000 W
PLW12 0.09795900 W
PLW13 0.04800000 W
F2 - Processing parameters
SI 32768
SF 150.9027860 MHz
WDW EM
SSB 0
LB 1.00 Hz
GB 0
PC 1.40
200
180
160
140
120
100
80
60
40
20
0
ppm

Compound 15

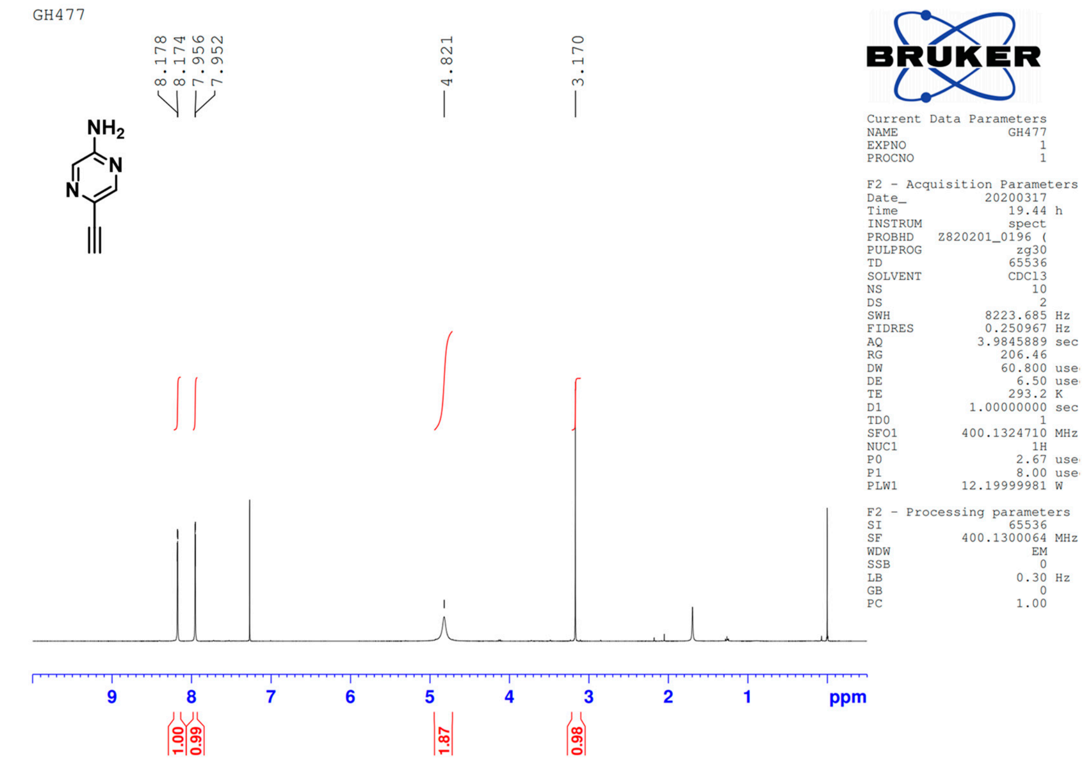
GH477
8.178
8.174
7.956
7.952
4.821
3.170
NH2
N
N
1.00
0.99
1.87
0.98
9 8 7 6 5 4 3 2 1 ppm
BRUKER
Current Data Parameters
NAME GH477
EXPNO 1
PROCNO 1
F2 - Acquisition Parameters
Date_ 20200317
Time 19.44 h
INSTRUM spect
PROBHD Z820201_0196 (
PULPROG zg30
TD 65536
SOLVENT CDCl3
NS 10
DS 2
SWH 8223.685 Hz
FIDRES 0.250967 Hz
AQ 3.9845889 sec
RG 206.46
DW 60.800 use
DE 6.50 use
TE 293.2 K
D1 1.00000000 sec
TD0 1
SFO1 400.1324710 MHz
NUC1 1H
P0 2.67 use
P1 8.00 use
PLW1 12.19999981 W
F2 - Processing parameters
SI 65536
SF 400.1300064 MHz
WDW EM
SSB 0
LB 0.30 Hz
GB 0
PC 1.00

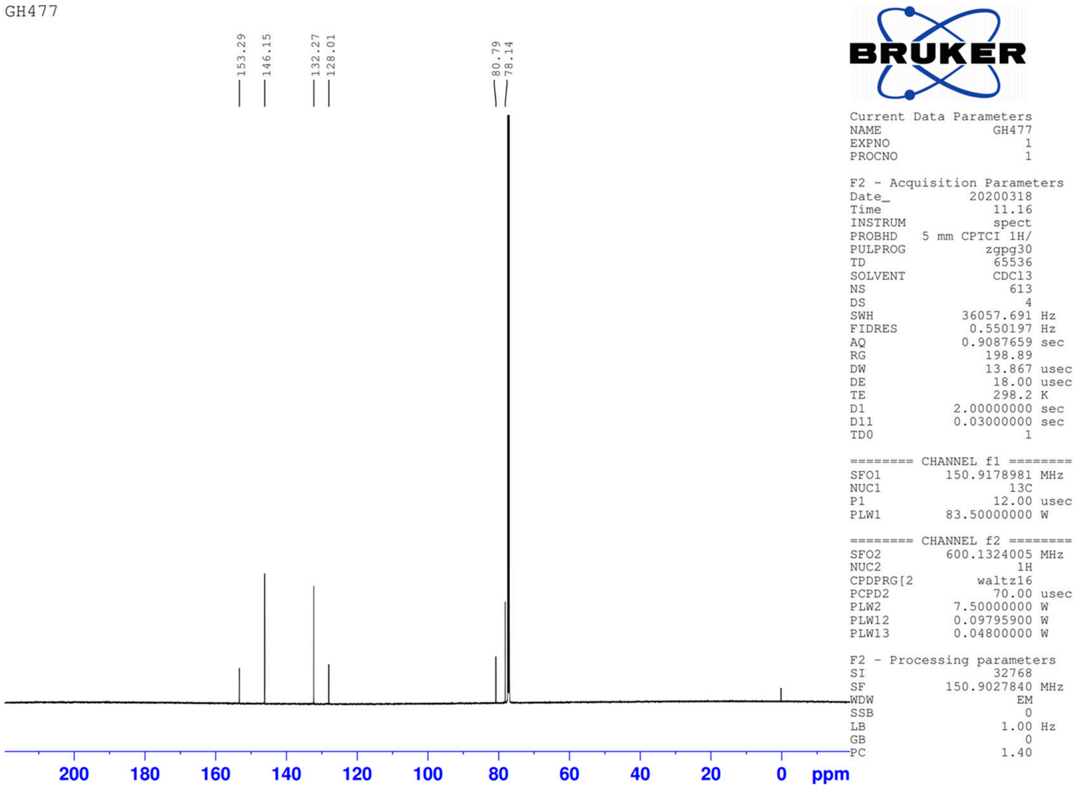
GH477
153.29
146.15
132.27
128.01
80.79
78.14
200 180 160 140 120 100 80 60 40 20 0 ppm
BRUKER
Current Data Parameters
NAME GH477
EXPNO 1
PROCNO 1
F2 - Acquisition Parameters
Date_ 20200318
Time 11.16
INSTRUM spect
PROBHD 5 mm CPTCI 1H/
PULPROG zgpg30
TD 65536
SOLVENT CDCl3
NS 613
DS 4
SWH 36057.691 Hz
FIDRES 0.550197 Hz
AQ 0.9087659 sec
RG 198.89
DW 13.867 usec
DE 18.00 usec
TE 298.2 K
D1 2.00000000 sec
D11 0.03000000 sec
TD0 1
======== CHANNEL f1 ========
SFO1 150.9178981 MHz
NUC1 13C
P1 12.00 usec
PLW1 83.50000000 W
======== CHANNEL f2 ========
SFO2 600.1324005 MHz
NUC2 1H
CPDPRG[2 waltz16
PCPD2 70.00 usec
PLW2 7.50000000 W
PLW12 0.09795900 W
PLW13 0.04800000 W
F2 - Processing parameters
SI 32768
SF 150.9027840 MHz
WDW EM
SSB 0
LB 1.00 Hz
GB 0
PC 1.40

Compound 17

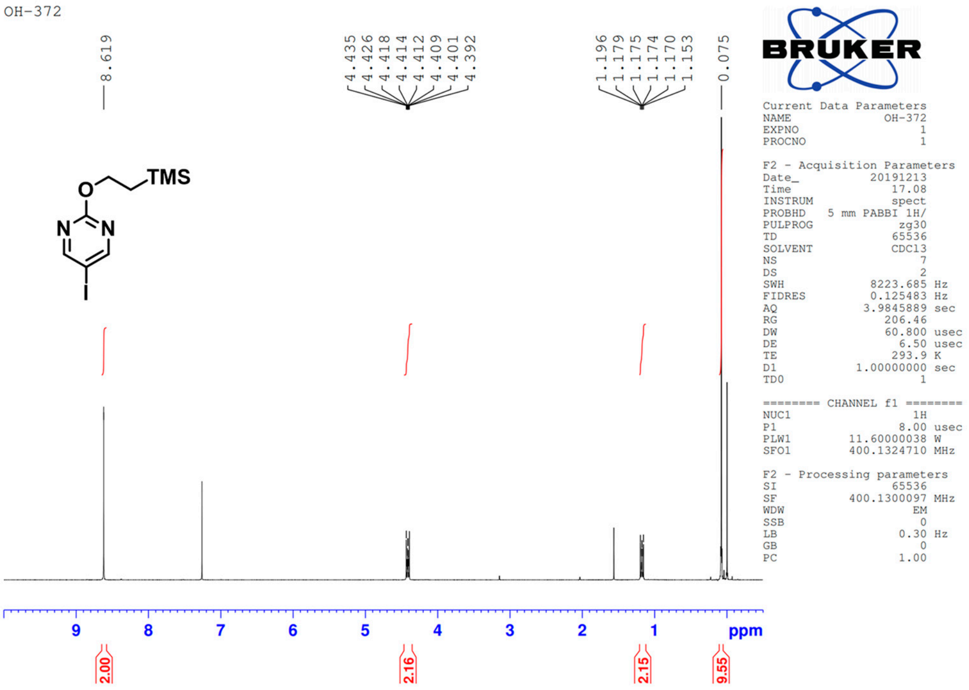

OH-372
8.619
4.435
4.426
4.418
4.414
4.412
4.409
4.401
4.392
1.196
1.179
1.175
1.174
1.170
1.153
0.075
O
TMS
N
N
I
BRUKER
Current Data Parameters
NAME OH-372
EXPNO 1
PROCNO 1
F2 - Acquisition Parameters
Date_ 20191213
Time 17.08
INSTRUM spect
PROBHD 5 mm PABBI 1H/
PULPROG zg30
TD 65536
SOLVENT CDCl3
NS 7
DS 2
SWH 8223.685 Hz
FIDRES 0.125483 Hz
AQ 3.9845889 sec
RG 206.46
DW 60.800 usec
DE 6.50 usec
TE 293.9 K
D1 1.00000000 sec
TD0 1
======== CHANNEL f1 ========
NUC1 1H
P1 8.00 usec
PLW1 11.60000038 W
SFO1 400.1324710 MHz
F2 - Processing parameters
SI 65536
SF 400.1300097 MHz
WDW EM
SSB 0
LB 0.30 Hz
GB 0
PC 1.00
9 8 7 6 5 4 3 2 1 ppm
2.00
2.16
2.15
9.55

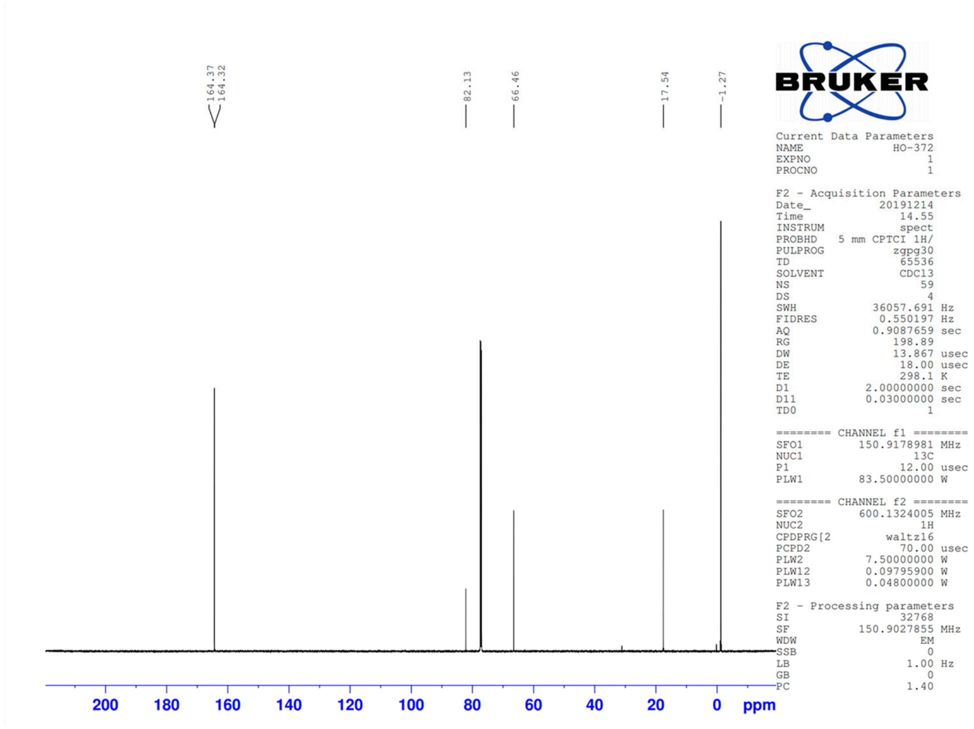

164.37
164.32
82.13
66.46
17.54
-1.27
BRUKER
Current Data Parameters
NAME HO-372
EXPNO 1
PROCNO 1
F2 - Acquisition Parameters
Date_ 20191214
Time 14.55
INSTRUM spect
PROBHD 5 mm CPTCI 1H/
PULPROG zgpg30
TD 65536
SOLVENT CDCl3
NS 59
DS 4
SWH 36057.691 Hz
FIDRES 0.550197 Hz
AQ 0.9087659 sec
RG 198.89
DW 13.867 usec
DE 18.00 usec
TE 298.1 K
D1 2.00000000 sec
D11 0.03000000 sec
TD0 1
======== CHANNEL f1 ========
SFO1 150.9178981 MHz
NUC1 13C
P1 12.00 usec
PLW1 83.50000000 W
======== CHANNEL f2 ========
SFO2 600.1324005 MHz
NUC2 1H
CPDPRG[2 waltz16
PCPD2 70.00 usec
PLW2 7.50000000 W
PLW12 0.09795900 W
PLW13 0.04800000 W
F2 - Processing parameters
SI 32768
SF 150.9027855 MHz
WDW EM
SSB 0
LB 1.00 Hz
GB 0
PC 1.40
200 180 160 140 120 100 80 60 40 20 0 ppm

Compound 18

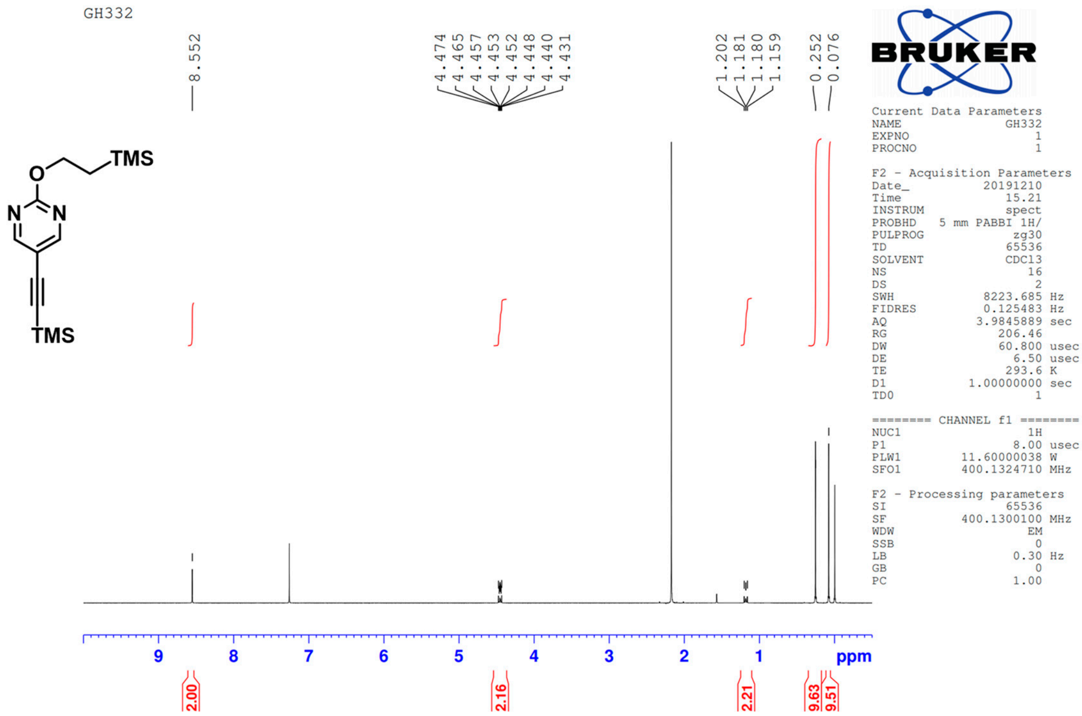
GH332
8.552
4.474
4.465
4.457
4.453
4.452
4.448
4.440
4.431
1.202
1.181
1.180
1.159
0.252
0.076
TMS
O
N
N
TMS
BRUKER
Current Data Parameters
NAME GH332
EXPNO 1
PROCNO 1
F2 - Acquisition Parameters
Date_ 20191210
Time 15.21
INSTRUM spect
PROBHD 5 mm PABBI 1H/
PULPROG zg30
TD 65536
SOLVENT CDCl3
NS 16
DS 2
SWH 8223.685 Hz
FIDRES 0.125483 Hz
AQ 3.9845889 sec
RG 206.46
DW 60.800 usec
DE 6.50 usec
TE 293.6 K
D1 1.00000000 sec
TD0 1
======== CHANNEL f1 ========
NUC1 1H
P1 8.00 usec
PLW1 11.60000038 W
SFO1 400.1324710 MHz
F2 - Processing parameters
SI 65536
SF 400.1300100 MHz
WDW EM
SSB 0
LB 0.30 Hz
GB 0
PC 1.00
9
8
7
6
5
4
3
2
1
ppm
2.00
2.16
2.21
9.63
9.51

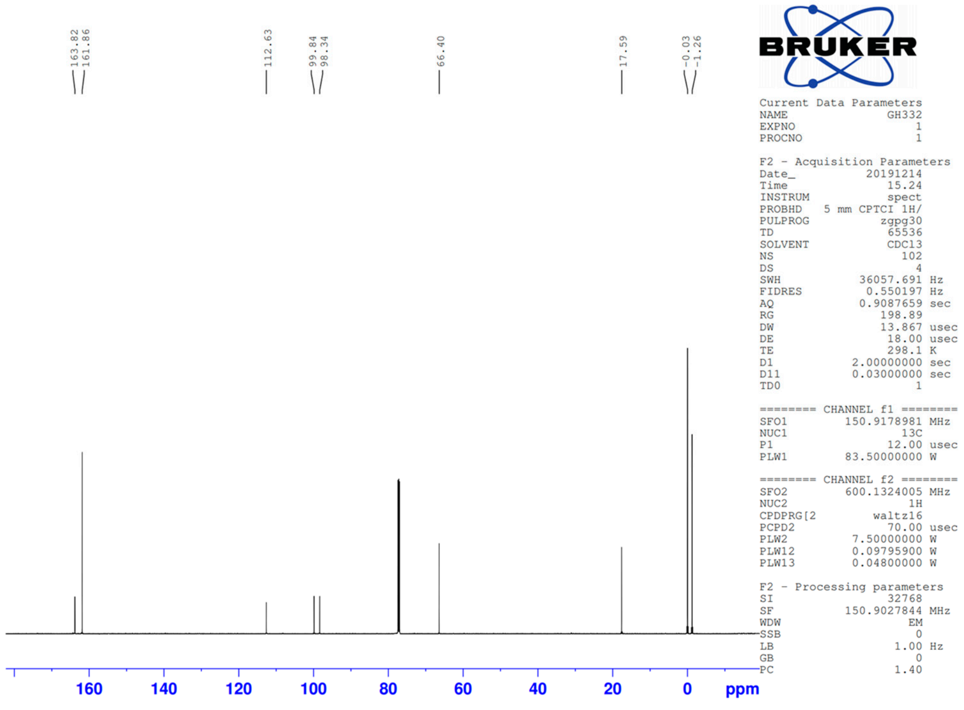
163.82
161.86
112.63
99.84
98.34
66.40
17.59
-0.03
-1.26
BRUKER
Current Data Parameters
NAME GH332
EXPNO 1
PROCNO 1
F2 - Acquisition Parameters
Date_ 20191214
Time 15.24
INSTRUM spect
PROBHD 5 mm CPTCI 1H/
PULPROG zgpg30
TD 65536
SOLVENT CDCl3
NS 102
DS 4
SWH 36057.691 Hz
FIDRES 0.550197 Hz
AQ 0.9087659 sec
RG 198.89
DW 13.867 usec
DE 18.00 usec
TE 298.1 K
D1 2.00000000 sec
D11 0.03000000 sec
TD0 1
======== CHANNEL f1 ========
SFO1 150.9178981 MHz
NUC1 13C
P1 12.00 usec
PLW1 83.50000000 W
======== CHANNEL f2 ========
SFO2 600.1324005 MHz
NUC2 1H
CPDPRG[2 waltz16
PCPD2 70.00 usec
PLW2 7.50000000 W
PLW12 0.09795900 W
PLW13 0.04800000 W
F2 - Processing parameters
SI 32768
SF 150.9027844 MHz
WDW EM
SSB 0
LB 1.00 Hz
GB 0
PC 1.40
160
140
120
100
80
60
40
20
0
ppm

Compound 19

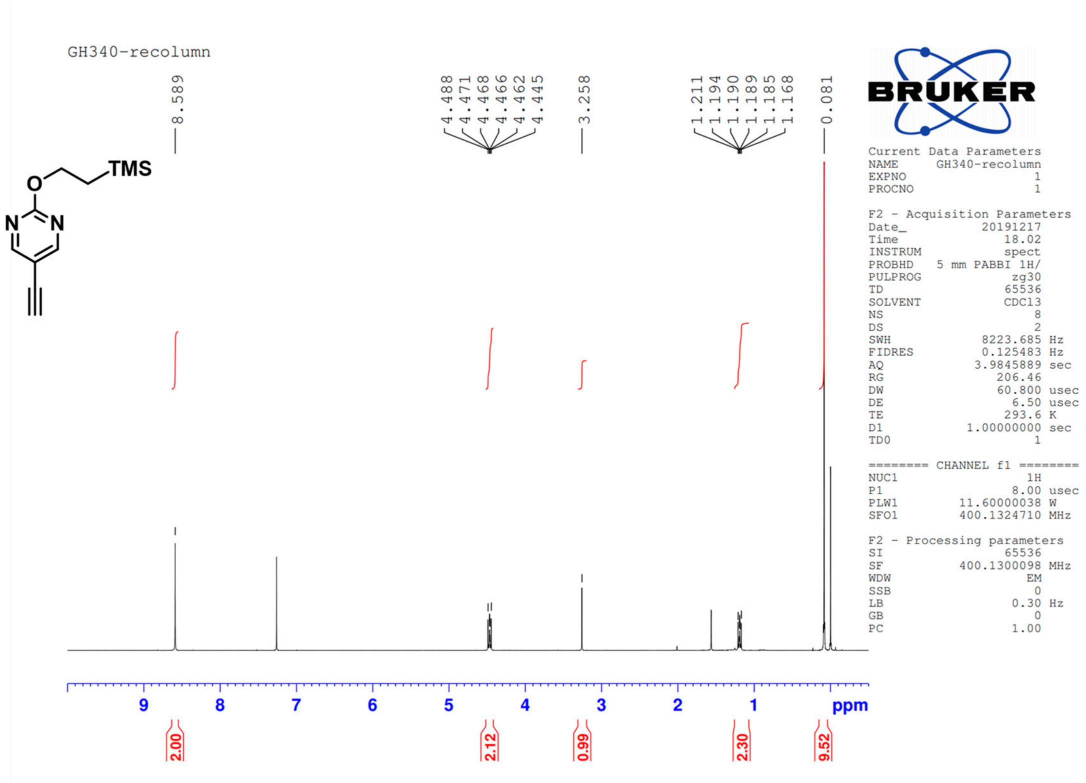
GH340-recolumn
TMS
O
N
N
8.589
4.488
4.471
4.468
4.466
4.462
4.445
3.258
1.211
1.194
1.190
1.189
1.185
1.168
0.081
BRUKER
Current Data Parameters
NAME GH340-recolumn
EXPNO 1
PROCNO 1
F2 - Acquisition Parameters
Date_ 20191217
Time 18.02
INSTRUM spect
PROBHD 5 mm PABBI 1H/
PULPROG zg30
TD 65536
SOLVENT CDCl3
NS 8
DS 2
SWH 8223.685 Hz
FIDRES 0.125483 Hz
AQ 3.9845889 sec
RG 206.46
DW 60.800 usec
DE 6.50 usec
TE 293.6 K
D1 1.00000000 sec
TD0 1
======== CHANNEL f1 ========
NUC1 1H
P1 8.00 usec
PLW1 11.60000038 W
SFO1 400.1324710 MHz
F2 - Processing parameters
SI 65536
SF 400.1300098 MHz
WDW EM
SSB 0
LB 0.30 Hz
GB 0
PC 1.00
9
8
7
6
5
4
3
2
1
ppm
2.00
2.12
0.99
2.30
9.52

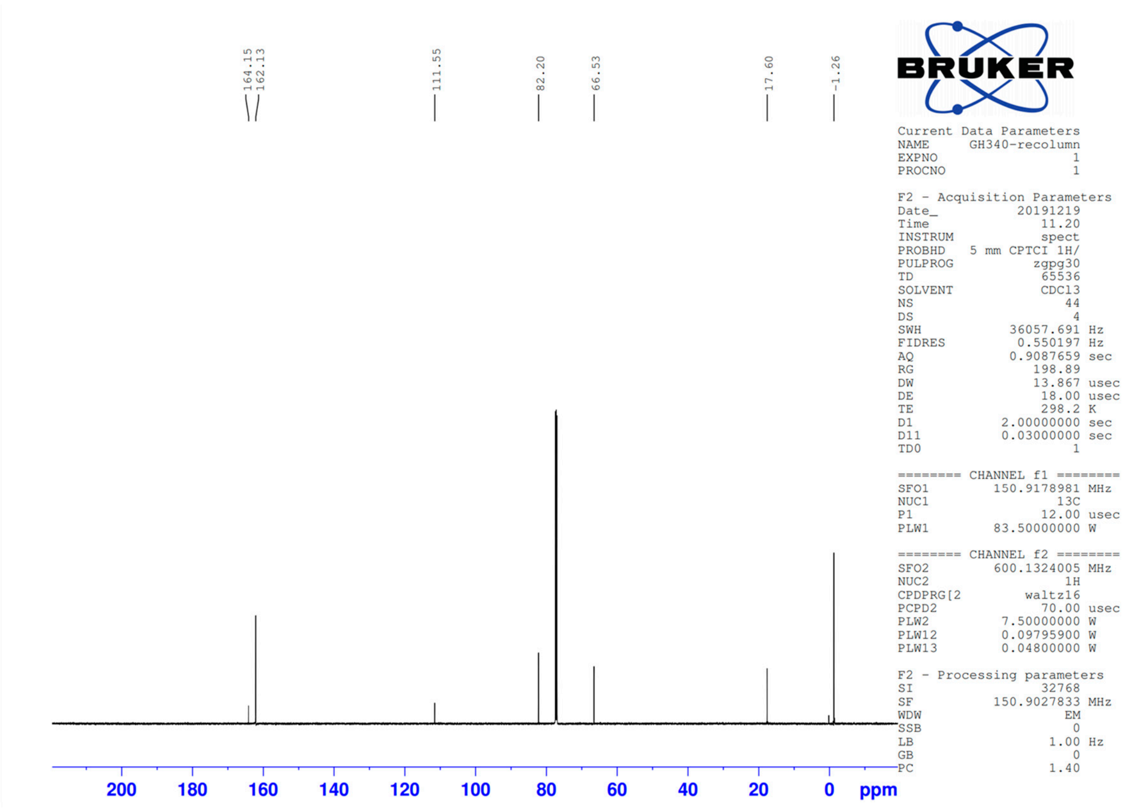
164.15
162.13
111.55
82.20
66.53
17.60
-1.26
BRUKER
Current Data Parameters
NAME GH340-recolumn
EXPNO 1
PROCNO 1
F2 - Acquisition Parameters
Date_ 20191219
Time 11.20
INSTRUM spect
PROBHD 5 mm CPTCI 1H/
PULPROG zgpg30
TD 65536
SOLVENT CDCl3
NS 44
DS 4
SWH 36057.691 Hz
FIDRES 0.550197 Hz
AQ 0.9087659 sec
RG 198.89
DW 13.867 usec
DE 18.00 usec
TE 298.2 K
D1 2.00000000 sec
D11 0.03000000 sec
TD0 1
======== CHANNEL f1 ========
SFO1 150.9178981 MHz
NUC1 13C
P1 12.00 usec
PLW1 83.50000000 W
======== CHANNEL f2 ========
SFO2 600.1324005 MHz
NUC2 1H
CPDPRG[2 waltz16
PCPD2 70.00 usec
PLW2 7.50000000 W
PLW12 0.09795900 W
PLW13 0.04800000 W
F2 - Processing parameters
SI 32768
SF 150.9027833 MHz
WDW EM
SSB 0
LB 1.00 Hz
GB 0
PC 1.40
200
180
160
140
120
100
80
60
40
20
0
ppm

Compound 21

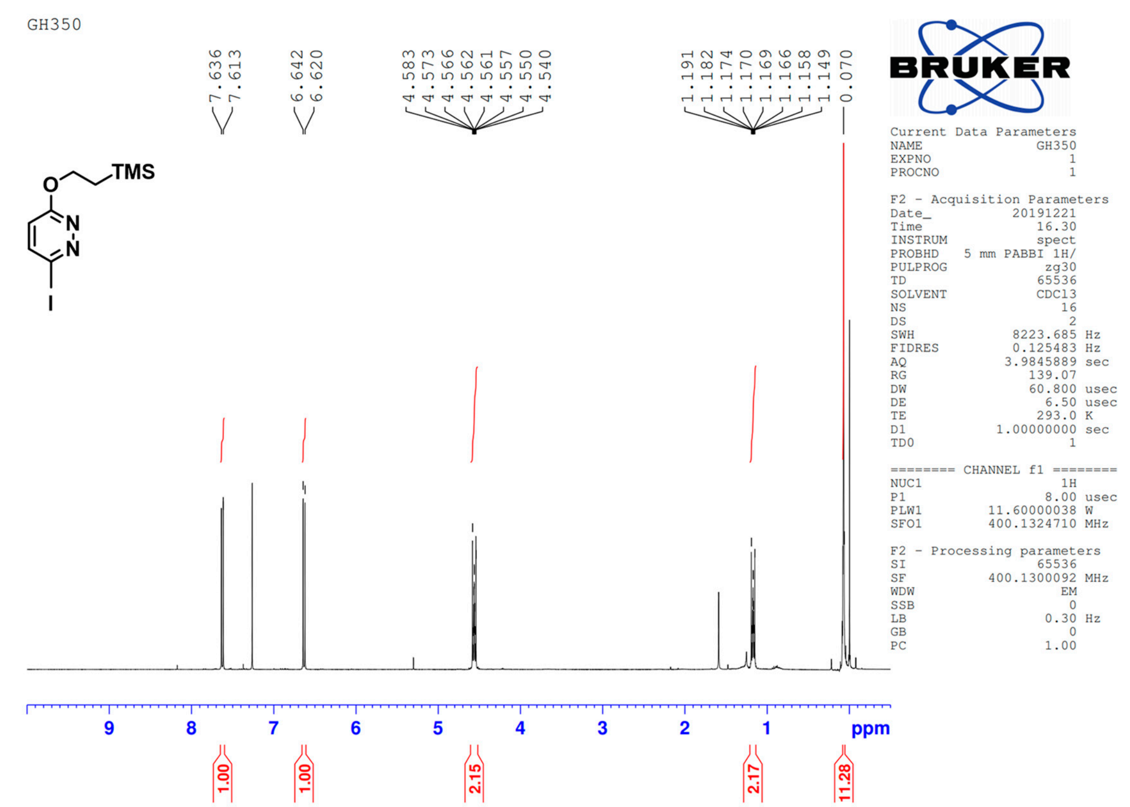
GH350
7.636
7.613
6.642
6.620
4.583
4.573
4.566
4.562
4.561
4.557
4.550
4.540
1.191
1.182
1.174
1.170
1.169
1.166
1.158
1.149
0.070
TMS
O
N
N
I
Current Data Parameters
NAME GH350
EXPNO 1
PROCNO 1
F2 - Acquisition Parameters
Date_ 20191221
Time 16.30
INSTRUM spect
PROBHD 5 mm PABBI 1H/
PULPROG zg30
TD 65536
SOLVENT CDCl3
NS 16
DS 2
SWH 8223.685 Hz
FIDRES 0.125483 Hz
AQ 3.9845889 sec
RG 139.07
DW 60.800 usec
DE 6.50 usec
TE 293.0 K
D1 1.00000000 sec
TD0 1
======== CHANNEL f1 ========
NUC1 1H
P1 8.00 usec
PLW1 11.60000038 W
SFO1 400.1324710 MHz
F2 - Processing parameters
SI 65536
SF 400.1300092 MHz
WDW EM
SSB 0
LB 0.30 Hz
GB 0
PC 1.00
9
8
7
6
5
4
3
2
1
ppm
1.00
1.00
2.15
2.17
11.28

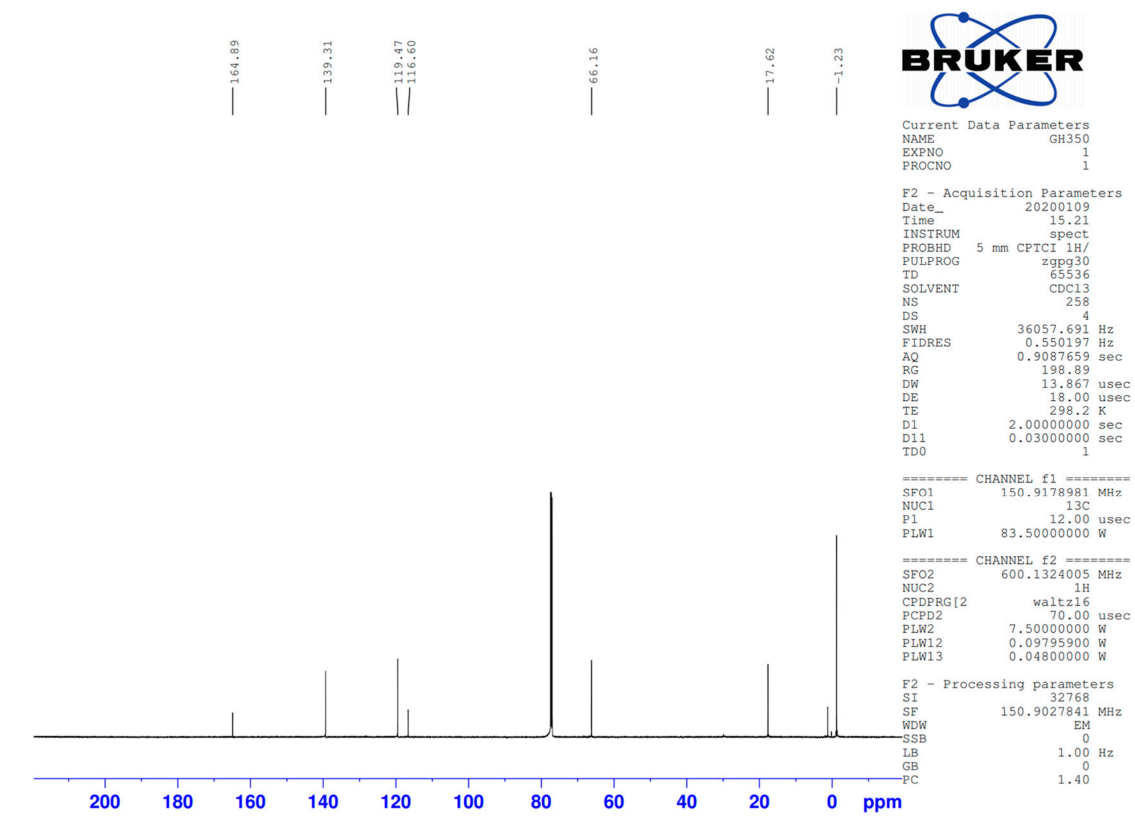
164.89
139.31
119.47
116.60
66.16
17.62
-1.23
Current Data Parameters
NAME GH350
EXPNO 1
PROCNO 1
F2 - Acquisition Parameters
Date_ 20200109
Time 15.21
INSTRUM spect
PROBHD 5 mm CPTCI 1H/
PULPROG zgpg30
TD 65536
SOLVENT CDCl3
NS 258
DS 4
SWH 36057.691 Hz
FIDRES 0.550197 Hz
AQ 0.9087659 sec
RG 198.89
DW 13.867 usec
DE 18.00 usec
TE 298.2 K
D1 2.00000000 sec
D11 0.03000000 sec
TD0 1
======== CHANNEL f1 ========
SFO1 150.9178981 MHz
NUC1 13C
P1 12.00 usec
PLW1 83.50000000 W
======== CHANNEL f2 ========
SFO2 600.1324005 MHz
NUC2 1H
CPDPRG[2 waltz16
PCPD2 70.00 usec
PLW2 7.50000000 W
PLW12 0.09795900 W
PLW13 0.04800000 W
F2 - Processing parameters
SI 32768
SF 150.9027841 MHz
WDW EM
SSB 0
LB 1.00 Hz
GB 0
PC 1.40
200
180
160
140
120
100
80
60
40
20
0
ppm

Compound 22

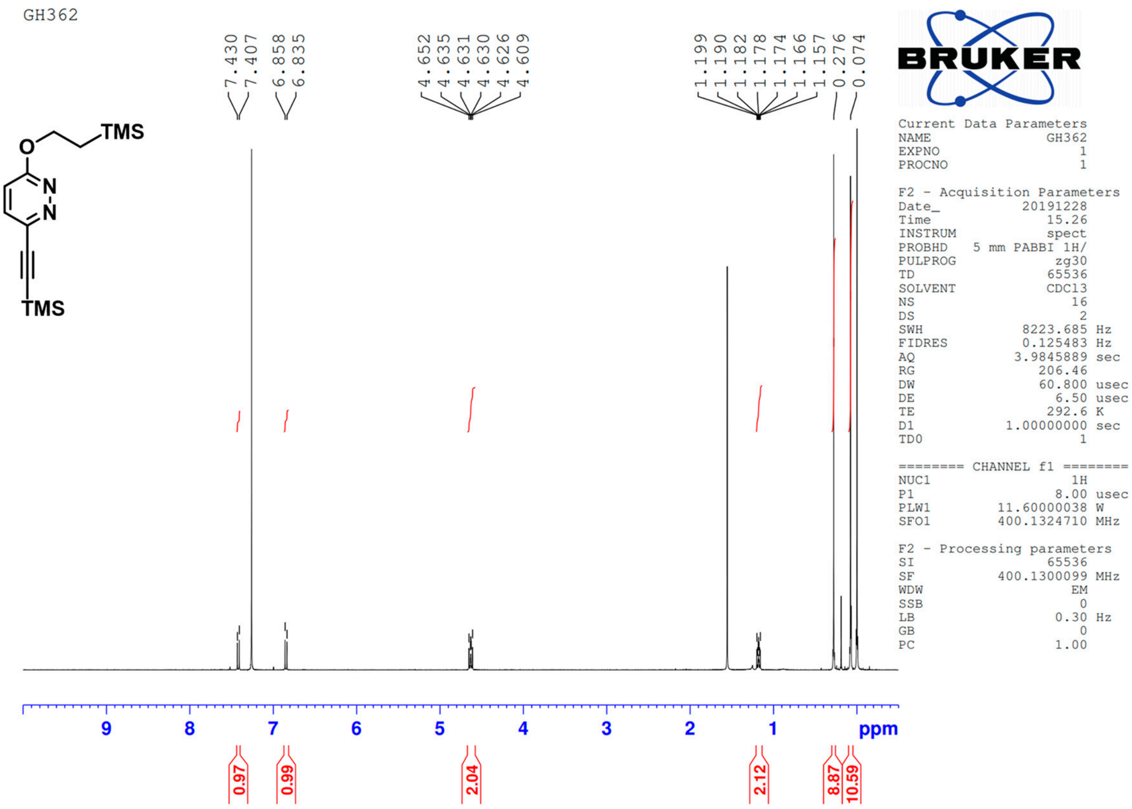
GH362
TMS
O
N
N
TMS
7.430
7.407
6.858
6.835
4.652
4.635
4.631
4.630
4.626
4.609
1.199
1.190
1.182
1.178
1.174
1.166
1.157
0.276
0.074
BRUKER
Current Data Parameters
NAME GH362
EXPNO 1
PROCNO 1
F2 - Acquisition Parameters
Date_ 20191228
Time 15.26
INSTRUM spect
PROBHD 5 mm PABBI 1H/
PULPROG zg30
TD 65536
SOLVENT CDCl3
NS 16
DS 2
SWH 8223.685 Hz
FIDRES 0.125483 Hz
AQ 3.9845889 sec
RG 206.46
DW 60.800 usec
DE 6.50 usec
TE 292.6 K
D1 1.00000000 sec
TD0 1
======== CHANNEL f1 ========
NUC1 1H
P1 8.00 usec
PLW1 11.60000038 W
SFO1 400.1324710 MHz
F2 - Processing parameters
SI 65536
SF 400.1300099 MHz
WDW EM
SSB 0
LB 0.30 Hz
GB 0
PC 1.00
9
8
7
6
5
4
3
2
1
ppm
0.97
0.99
2.04
2.12
8.87
10.59

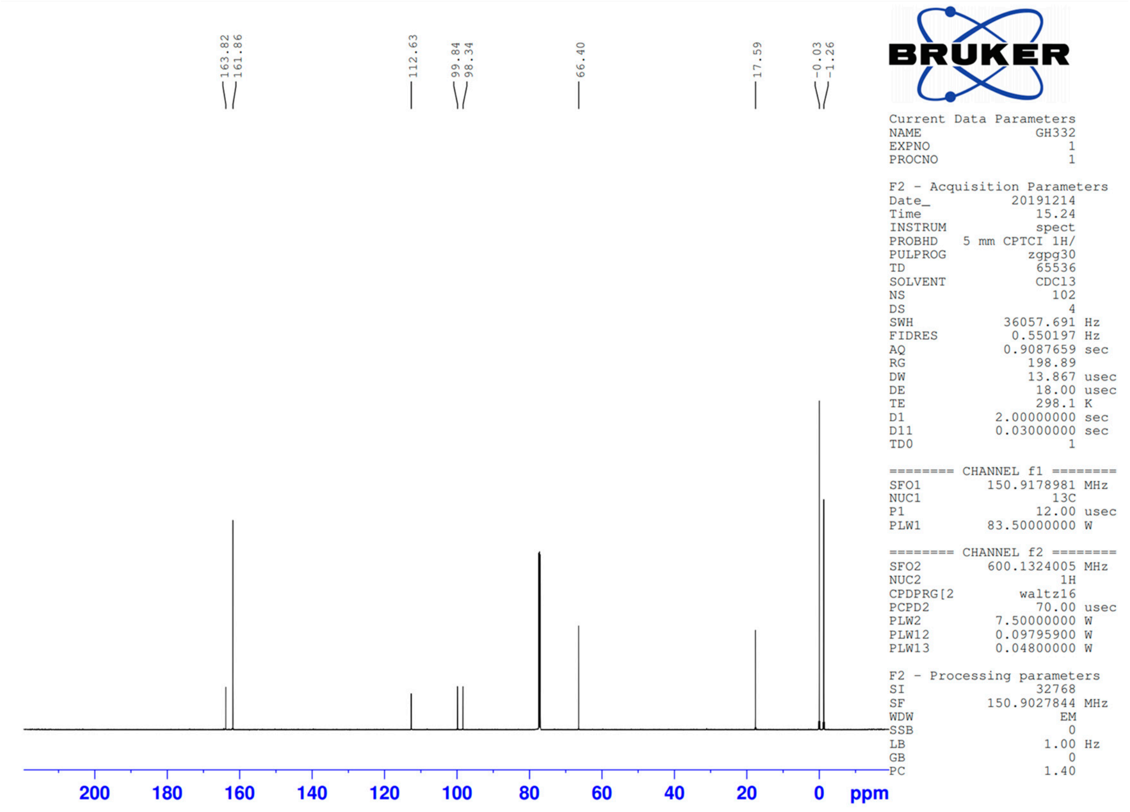
163.82
161.86
112.63
99.84
98.34
66.40
17.59
-0.03
-1.26
BRUKER
Current Data Parameters
NAME GH332
EXPNO 1
PROCNO 1
F2 - Acquisition Parameters
Date_ 20191214
Time 15.24
INSTRUM spect
PROBHD 5 mm CPTCI 1H/
PULPROG zgpg30
TD 65536
SOLVENT CDCl3
NS 102
DS 4
SWH 36057.691 Hz
FIDRES 0.550197 Hz
AQ 0.9087659 sec
RG 198.89
DW 13.867 usec
DE 18.00 usec
TE 298.1 K
D1 2.00000000 sec
D11 0.03000000 sec
TD0 1
======== CHANNEL f1 ========
SFO1 150.9178981 MHz
NUC1 13C
P1 12.00 usec
PLW1 83.50000000 W
======== CHANNEL f2 ========
SFO2 600.1324005 MHz
NUC2 1H
CPDPRG[2 waltz16
PCPD2 70.00 usec
PLW2 7.50000000 W
PLW12 0.09795900 W
PLW13 0.04800000 W
F2 - Processing parameters
SI 32768
SF 150.9027844 MHz
WDW EM
SSB 0
LB 1.00 Hz
GB 0
PC 1.40
200
180
160
140
120
100
80
60
40
20
0
ppm

Compound 23

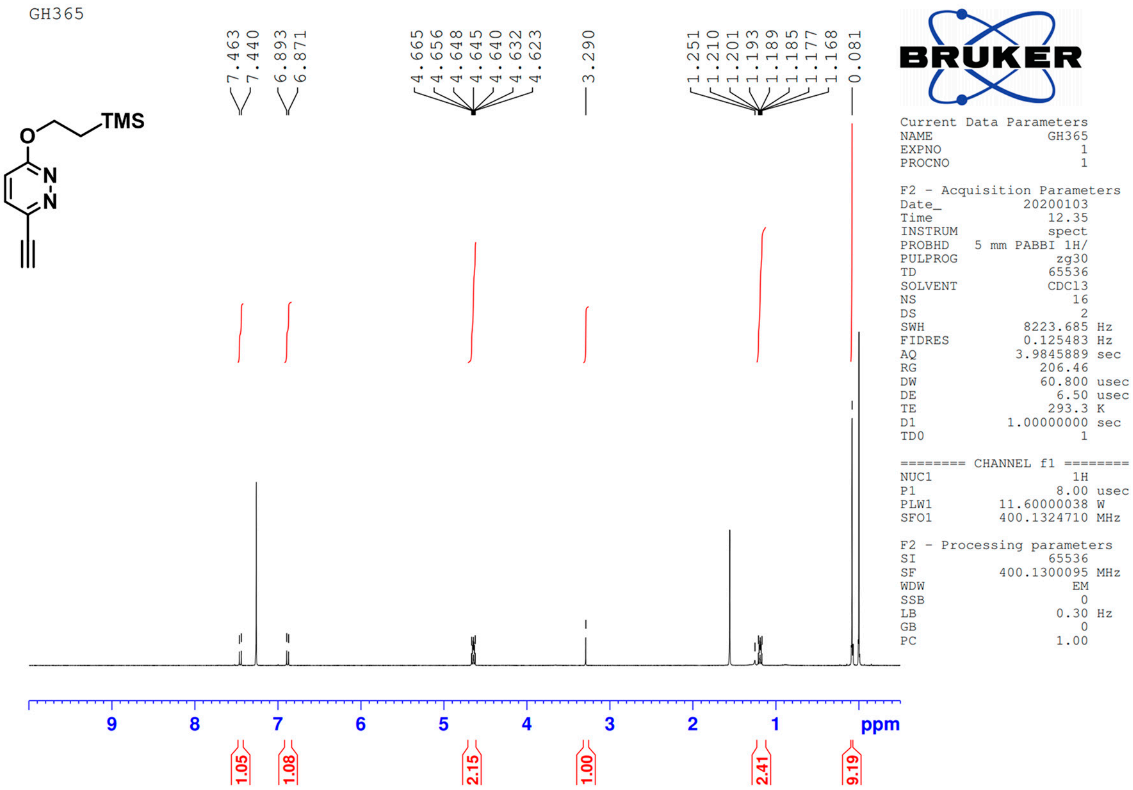

GH365
TMS
O
N
N
7.463
7.440
6.893
6.871
4.665
4.656
4.648
4.645
4.640
4.632
4.623
3.290
1.251
1.210
1.201
1.193
1.189
1.185
1.177
1.168
0.081
9
8
7
6
5
4
3
2
1
ppm
1.05
1.08
2.15
1.00
2.41
9.19
BRUKER
Current Data Parameters
NAME GH365
EXPNO 1
PROCNO 1
F2 - Acquisition Parameters
Date_ 20200103
Time 12.35
INSTRUM spect
PROBHD 5 mm PABBI 1H/
PULPROG zg30
TD 65536
SOLVENT CDCl3
NS 16
DS 2
SWH 8223.685 Hz
FIDRES 0.125483 Hz
AQ 3.9845889 sec
RG 206.46
DW 60.800 usec
DE 6.50 usec
TE 293.3 K
D1 1.00000000 sec
TD0 1
======== CHANNEL f1 ========
NUC1 1H
P1 8.00 usec
PLW1 11.60000038 W
SFO1 400.1324710 MHz
F2 - Processing parameters
SI 65536
SF 400.1300095 MHz
WDW EM
SSB 0
LB 0.30 Hz
GB 0
PC 1.00

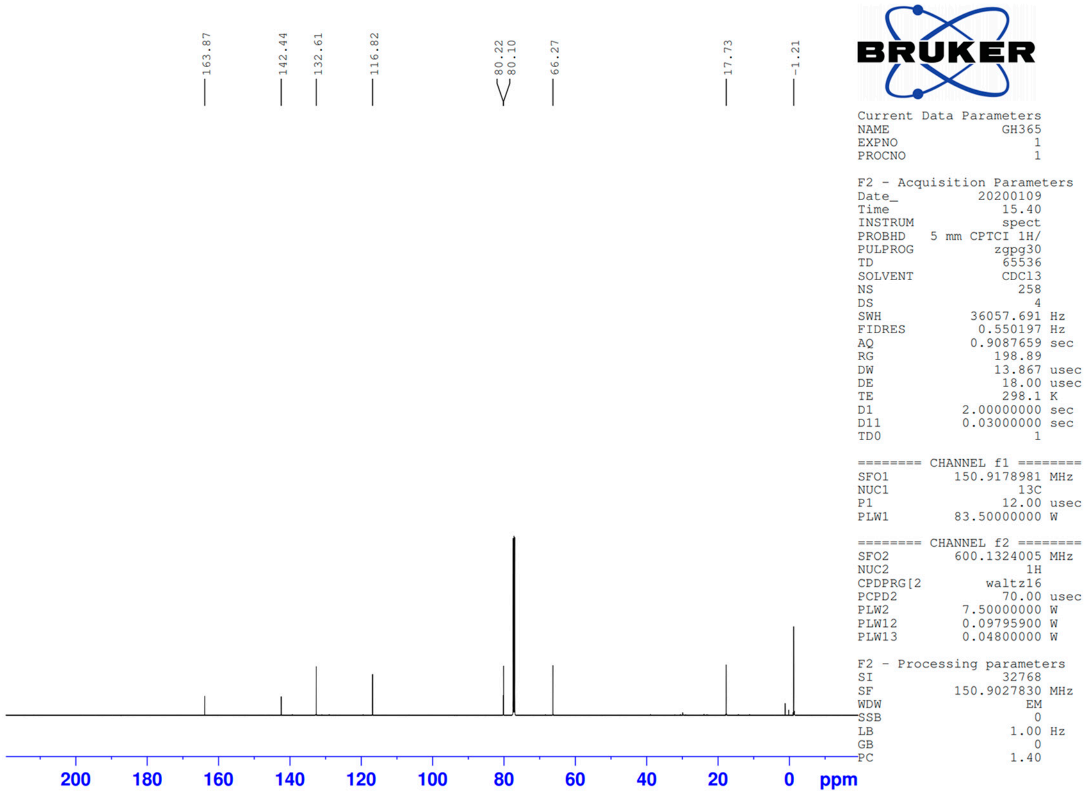

163.87
142.44
132.61
116.82
80.22
80.10
66.27
17.73
-1.21
200
180
160
140
120
100
80
60
40
20
0
ppm
BRUKER
Current Data Parameters
NAME GH365
EXPNO 1
PROCNO 1
F2 - Acquisition Parameters
Date_ 20200109
Time 15.40
INSTRUM spect
PROBHD 5 mm CPTCI 1H/
PULPROG zgpg30
TD 65536
SOLVENT CDCl3
NS 258
DS 4
SWH 36057.691 Hz
FIDRES 0.550197 Hz
AQ 0.9087659 sec
RG 198.89
DW 13.867 usec
DE 18.00 usec
TE 298.1 K
D1 2.00000000 sec
D11 0.03000000 sec
TD0 1
======== CHANNEL f1 ========
SFO1 150.9178981 MHz
NUC1 13C
P1 12.00 usec
PLW1 83.50000000 W
======== CHANNEL f2 ========
SFO2 600.1324005 MHz
NUC2 1H
CPDPRG[2 waltz16
PCPD2 70.00 usec
PLW2 7.50000000 W
PLW12 0.09795900 W
PLW13 0.04800000 W
F2 - Processing parameters
SI 32768
SF 150.9027830 MHz
WDW EM
SSB 0
LB 1.00 Hz
GB 0
PC 1.40

Compound 27

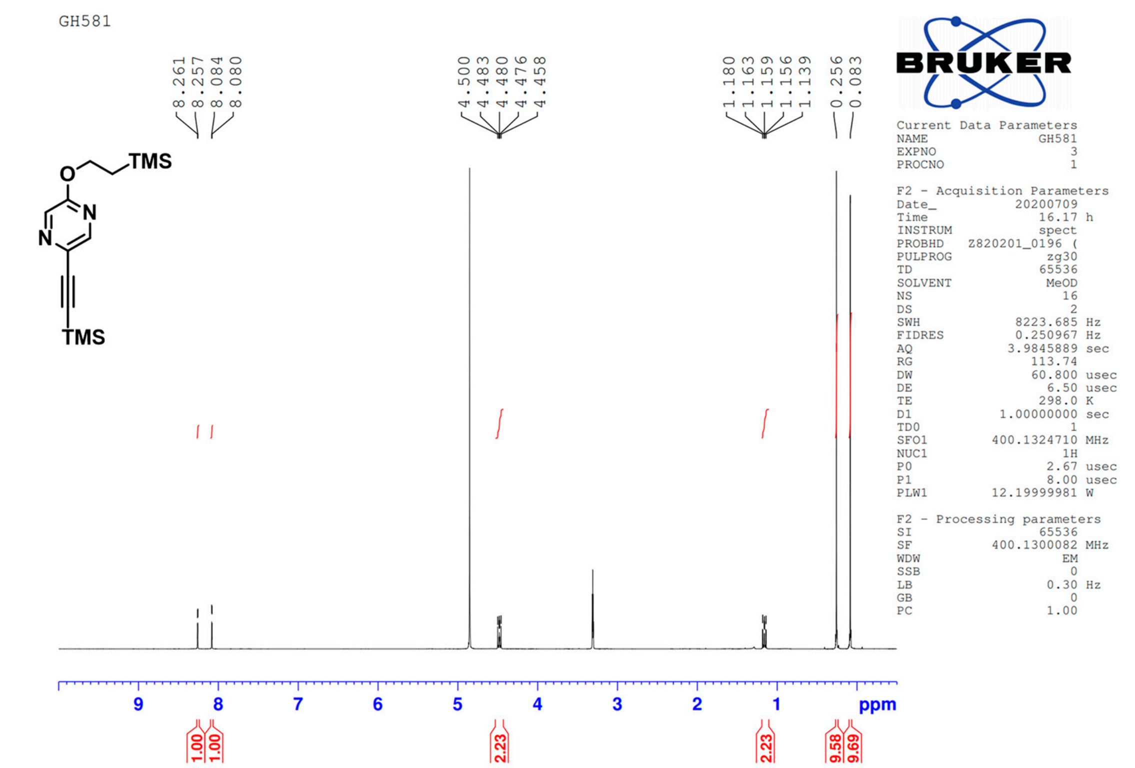
GH581
8.261
8.257
8.084
8.080
4.500
4.483
4.480
4.476
4.458
1.180
1.163
1.159
1.156
1.139
0.256
0.083
TMS
O
N
N
TMS
BRUKER
Current Data Parameters
NAME GH581
EXPNO 3
PROCNO 1
F2 - Acquisition Parameters
Date_ 20200709
Time 16.17 h
INSTRUM spect
PROBHD Z820201_0196 (
PULPROG zg30
TD 65536
SOLVENT MeOD
NS 16
DS 2
SWH 8223.685 Hz
FIDRES 0.250967 Hz
AQ 3.9845889 sec
RG 113.74
DW 60.800 usec
DE 6.50 usec
TE 298.0 K
D1 1.00000000 sec
TD0 1
SFO1 400.1324710 MHz
NUC1 1H
P0 2.67 usec
P1 8.00 usec
PLW1 12.19999981 W
F2 - Processing parameters
SI 65536
SF 400.1300082 MHz
WDW EM
SSB 0
LB 0.30 Hz
GB 0
PC 1.00
9
8
7
6
5
4
3
2
1
ppm
1.00
1.00
2.23
2.23
9.58
9.69

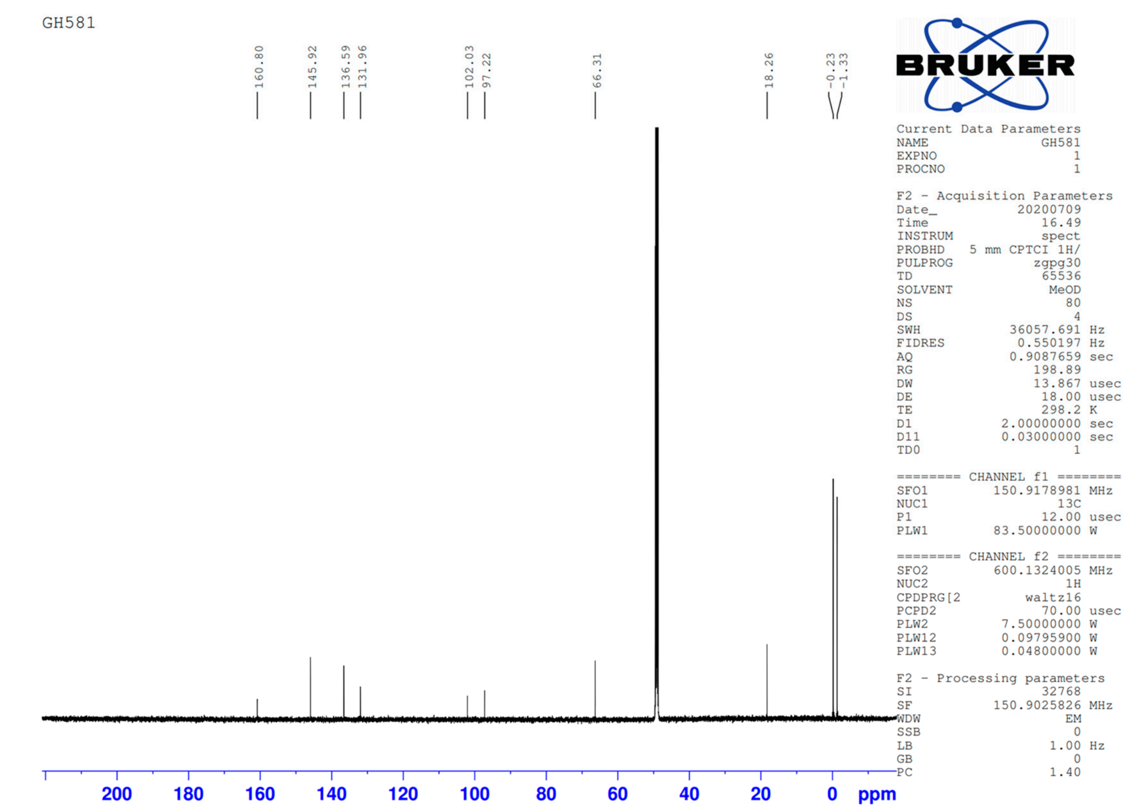
GH581
160.80
145.92
136.59
131.96
102.03
97.22
66.31
18.26
-0.23
-1.33
BRUKER
Current Data Parameters
NAME GH581
EXPNO 1
PROCNO 1
F2 - Acquisition Parameters
Date_ 20200709
Time 16.49
INSTRUM spect
PROBHD 5 mm CPTCI 1H/
PULPROG zgpg30
TD 65536
SOLVENT MeOD
NS 80
DS 4
SWH 36057.691 Hz
FIDRES 0.550197 Hz
AQ 0.9087659 sec
RG 198.89
DW 13.867 usec
DE 18.00 usec
TE 298.2 K
D1 2.00000000 sec
D11 0.03000000 sec
TD0 1
======== CHANNEL f1 ========
SFO1 150.9178981 MHz
NUC1 13C
P1 12.00 usec
PLW1 83.50000000 W
======== CHANNEL f2 ========
SFO2 600.1324005 MHz
NUC2 1H
CPDPRG[2 waltz16
PCPD2 70.00 usec
PLW2 7.50000000 W
PLW12 0.09795900 W
PLW13 0.04800000 W
F2 - Processing parameters
SI 32768
SF 150.9025826 MHz
WDW EM
SSB 0
LB 1.00 Hz
GB 0
PC 1.40
200
180
160
140
120
100
80
60
40
20
0
ppm

Compound 28

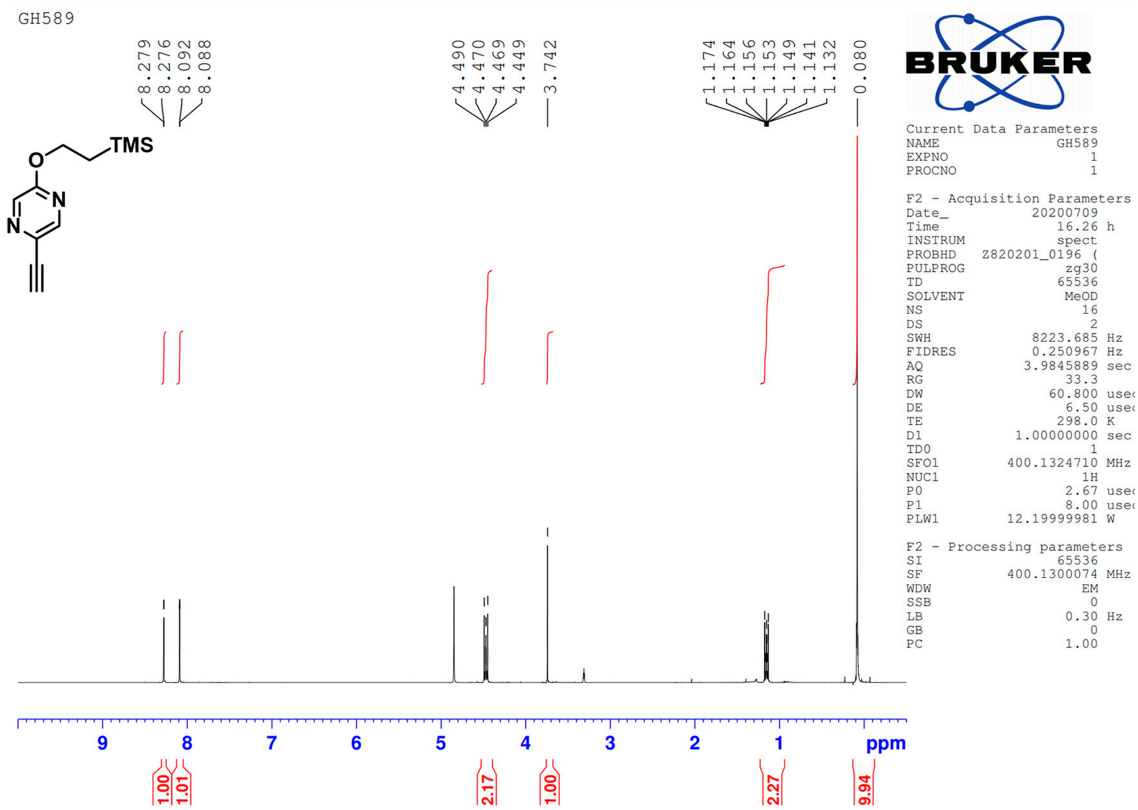
GH589
8.279
8.276
8.092
8.088
4.490
4.470
4.469
4.449
3.742
1.174
1.164
1.156
1.153
1.149
1.141
1.132
0.080
O
TMS
N
N
BRUKER
Current Data Parameters
NAME GH589
EXPNO 1
PROCNO 1
F2 - Acquisition Parameters
Date_ 20200709
Time 16.26 h
INSTRUM spect
PROBHD Z820201_0196 (
PULPROG zg30
TD 65536
SOLVENT MeOD
NS 16
DS 2
SWH 8223.685 Hz
FIDRES 0.250967 Hz
AQ 3.9845889 sec
RG 33.3
DW 60.800 usec
DE 6.50 usec
TE 298.0 K
D1 1.00000000 sec
TD0 1
SFO1 400.1324710 MHz
NUC1 1H
P0 2.67 usec
P1 8.00 usec
PLW1 12.19999981 W
F2 - Processing parameters
SI 65536
SF 400.1300074 MHz
WDW EM
SSB 0
LB 0.30 Hz
GB 0
PC 1.00
9
8
7
6
5
4
3
2
1
ppm
1.00
1.01
2.17
1.00
2.27
9.94

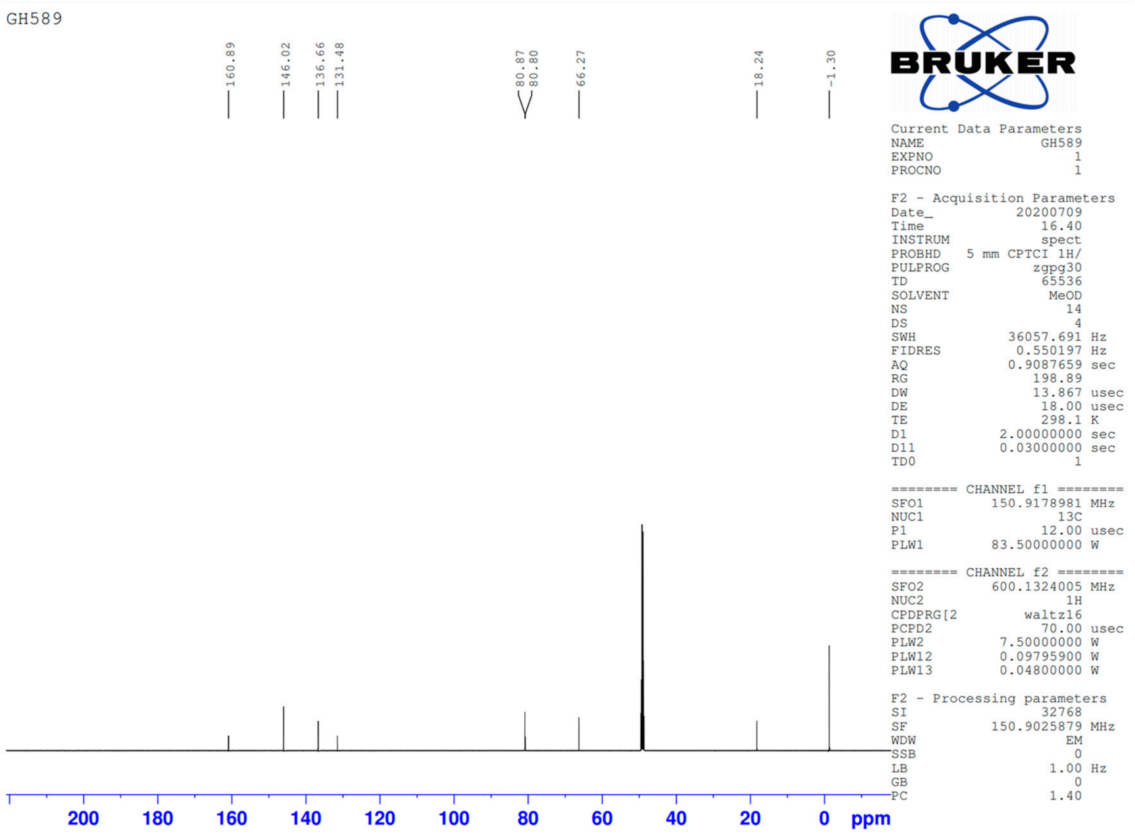
GH589
160.89
146.02
136.66
131.48
80.87
80.80
66.27
18.24
-1.30
BRUKER
Current Data Parameters
NAME GH589
EXPNO 1
PROCNO 1
F2 - Acquisition Parameters
Date_ 20200709
Time 16.40
INSTRUM spect
PROBHD 5 mm CPTCI 1H/
PULPROG zgpg30
TD 65536
SOLVENT MeOD
NS 14
DS 4
SWH 36057.691 Hz
FIDRES 0.550197 Hz
AQ 0.9087659 sec
RG 198.89
DW 13.867 usec
DE 18.00 usec
TE 298.1 K
D1 2.00000000 sec
D11 0.03000000 sec
TD0 1
======== CHANNEL f1 ========
SFO1 150.9178981 MHz
NUC1 13C
P1 12.00 usec
PLW1 83.50000000 W
======== CHANNEL f2 ========
SFO2 600.1324005 MHz
NUC2 1H
CPDPRG[2 waltz16
PCPD2 70.00 usec
PLW2 7.50000000 W
PLW12 0.09795900 W
PLW13 0.04800000 W
F2 - Processing parameters
SI 32768
SF 150.9025879 MHz
WDW EM
SSB 0
LB 1.00 Hz
GB 0
PC 1.40
200
180
160
140
120
100
80
60
40
20
0
ppm

5. MALDI-TOF MS charts of the synthesized ODNs

ODN1-^{N}Pu1

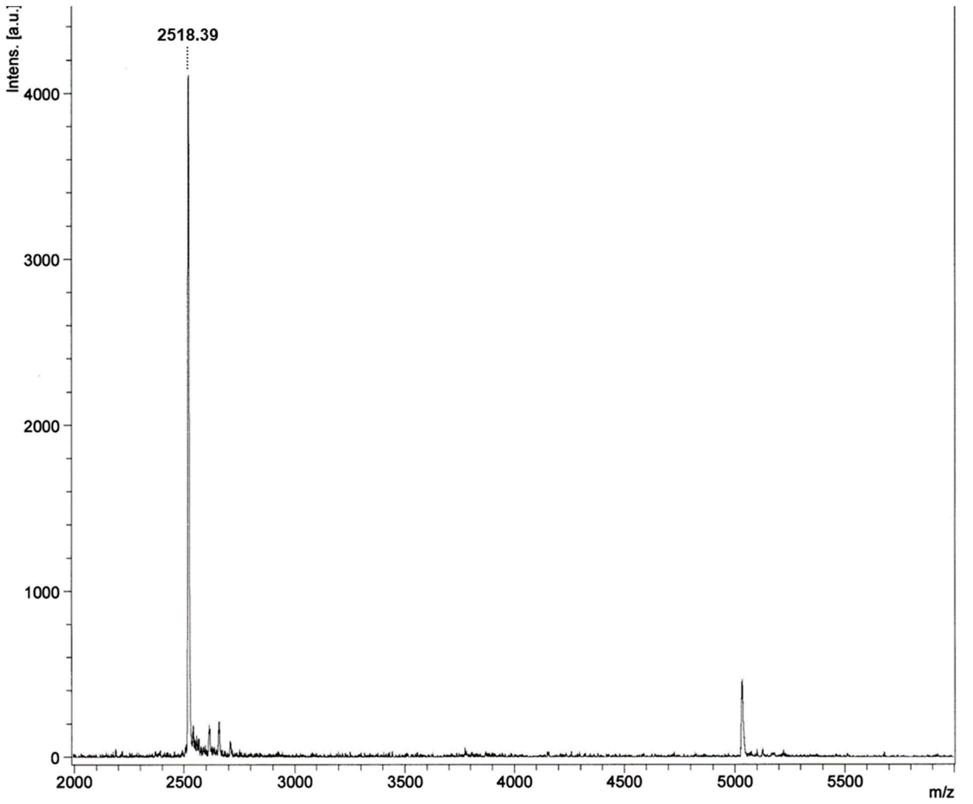

ODN3-^{N}Pu1

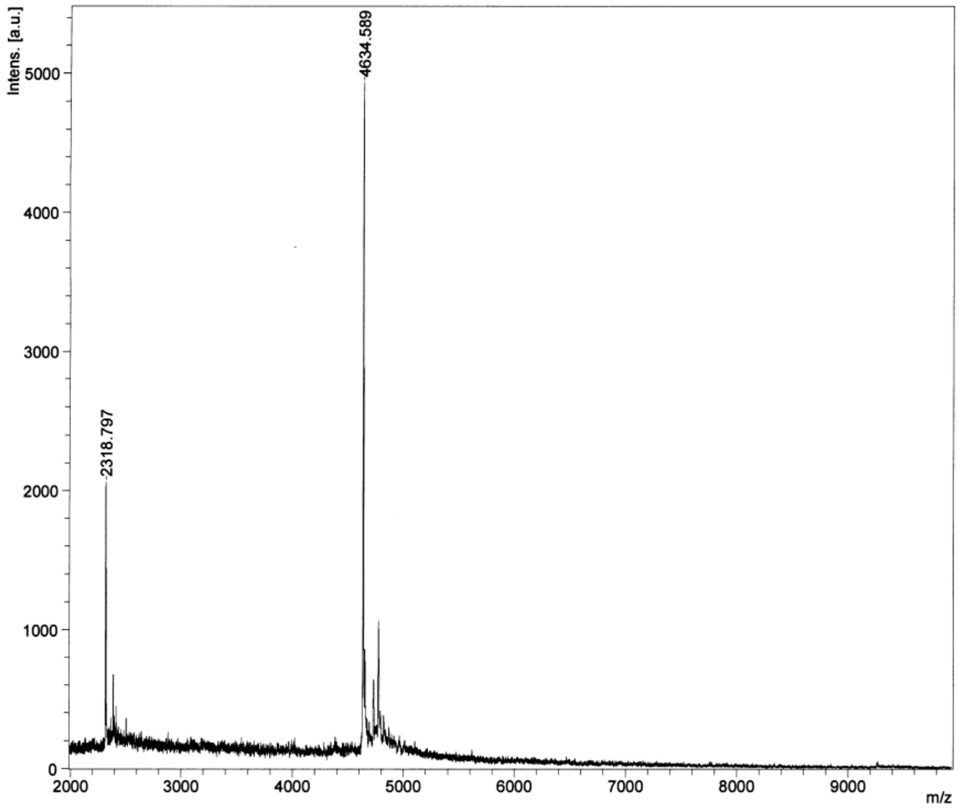

ODN3-^{N}Pu2

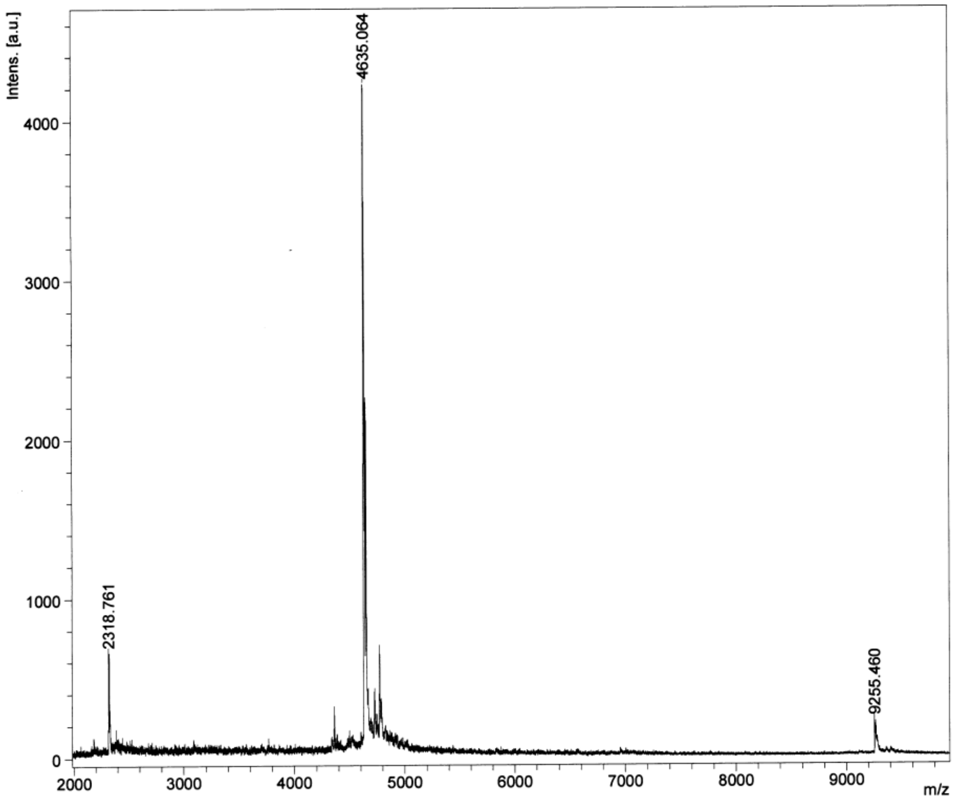

ODN3-^{N}Pu3

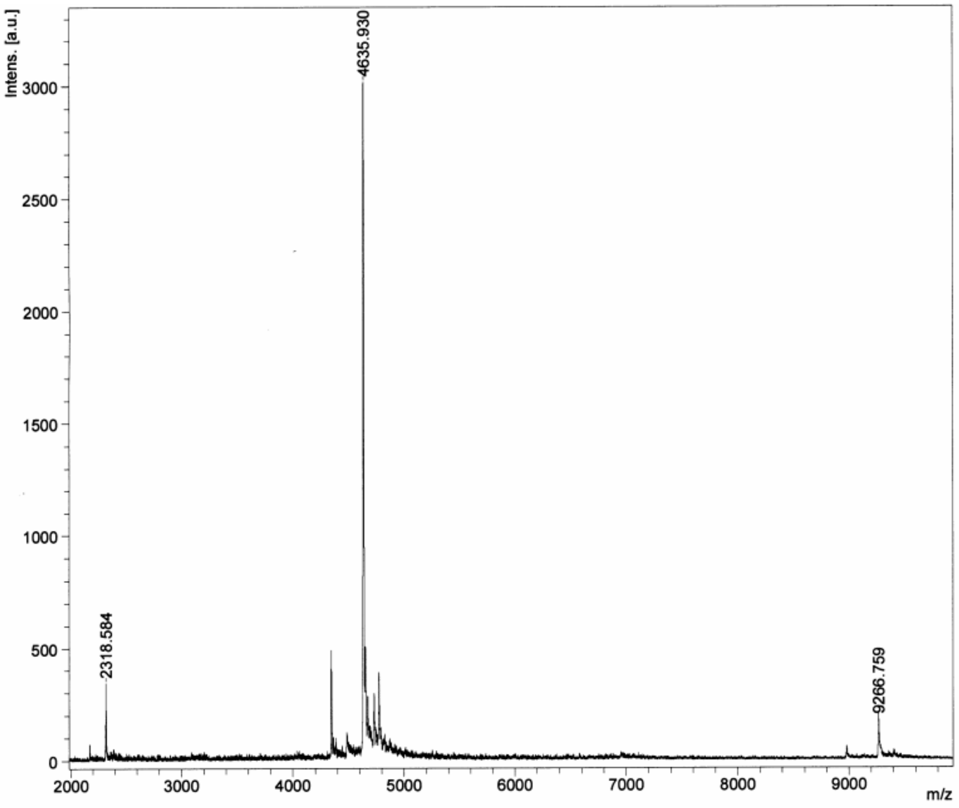

ODN3-^{O}Pu1

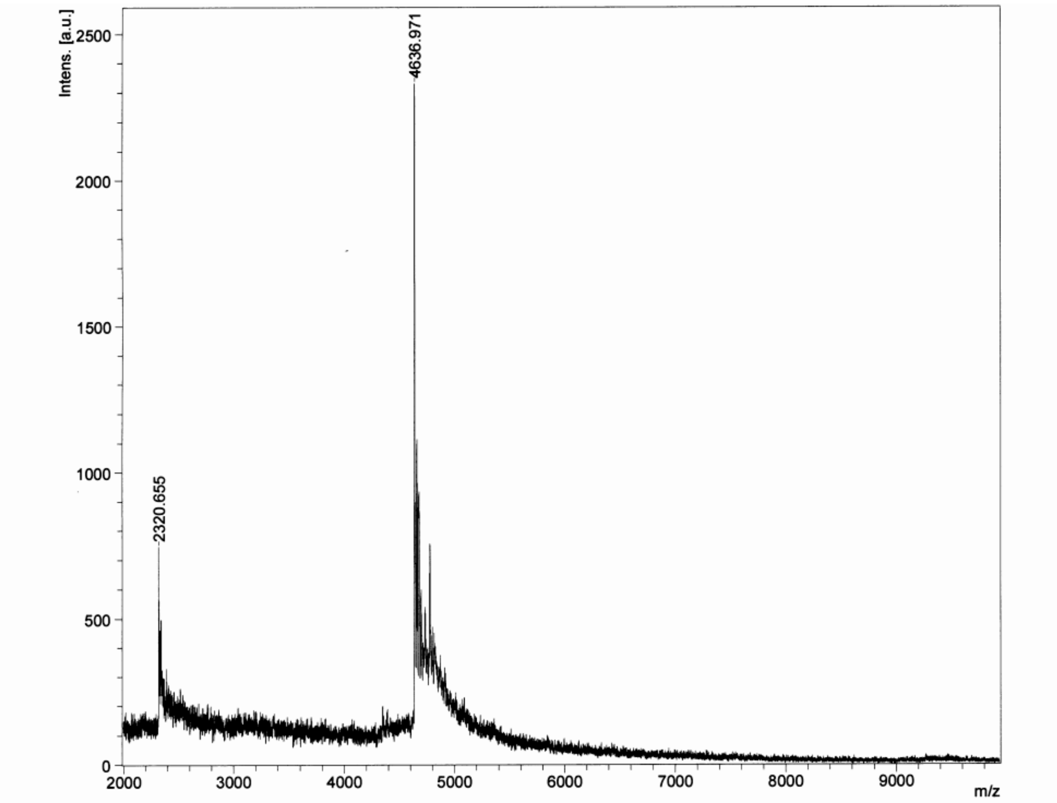

ODN3-^{O}Pu2

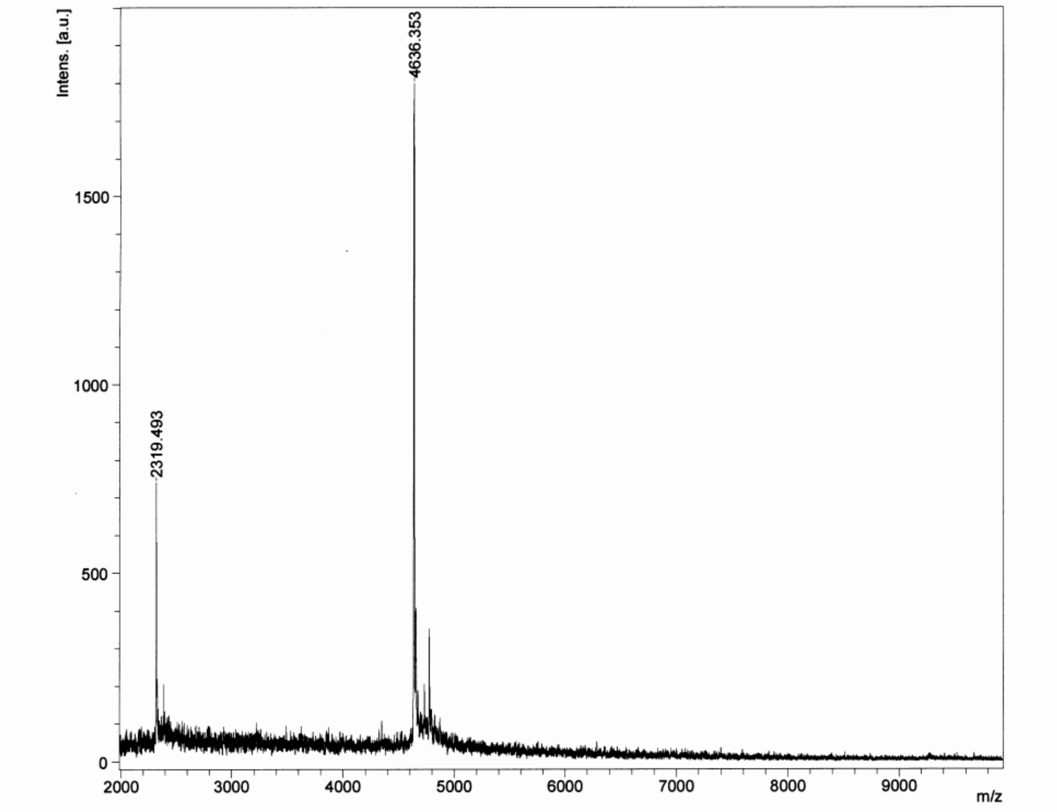

ODN3-^{O}Pu3

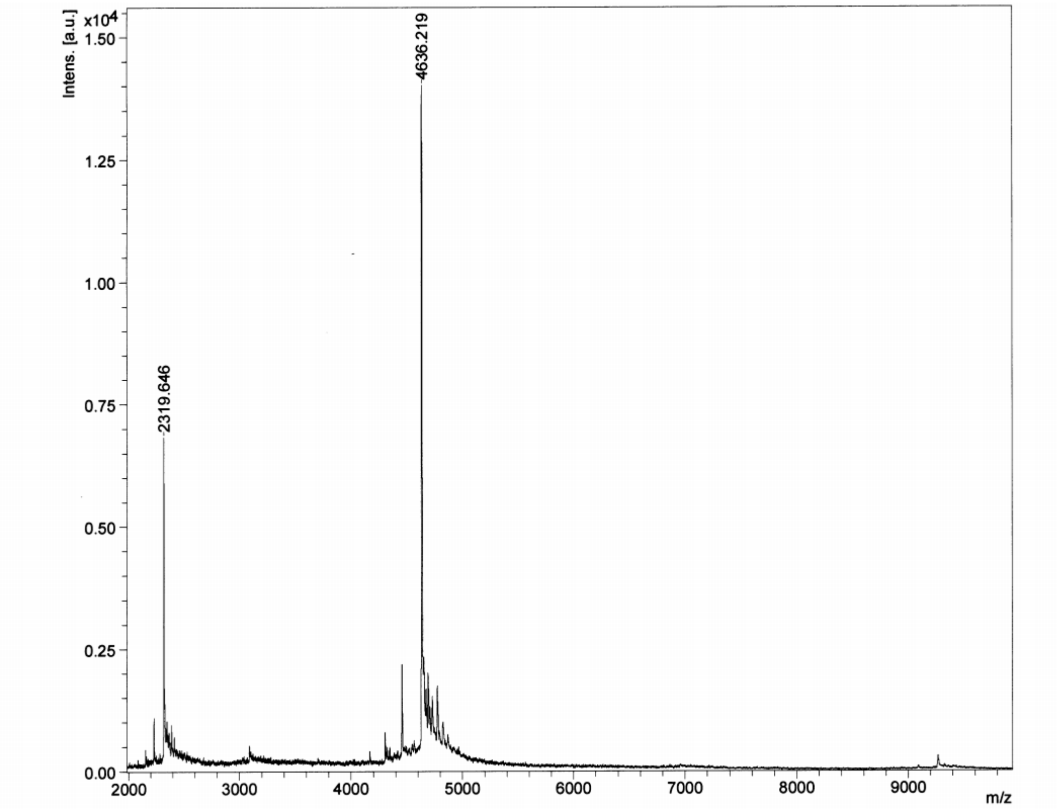

ODN4-^{N}Pu1

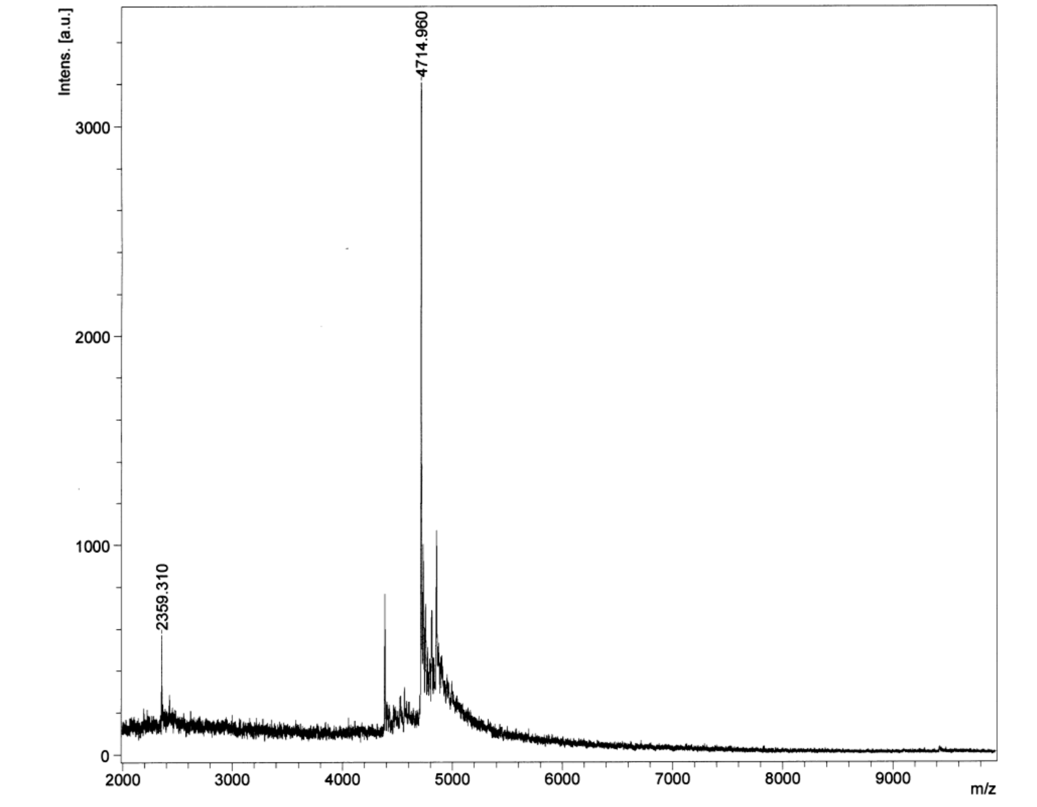

ODN4-^{N}Pu2

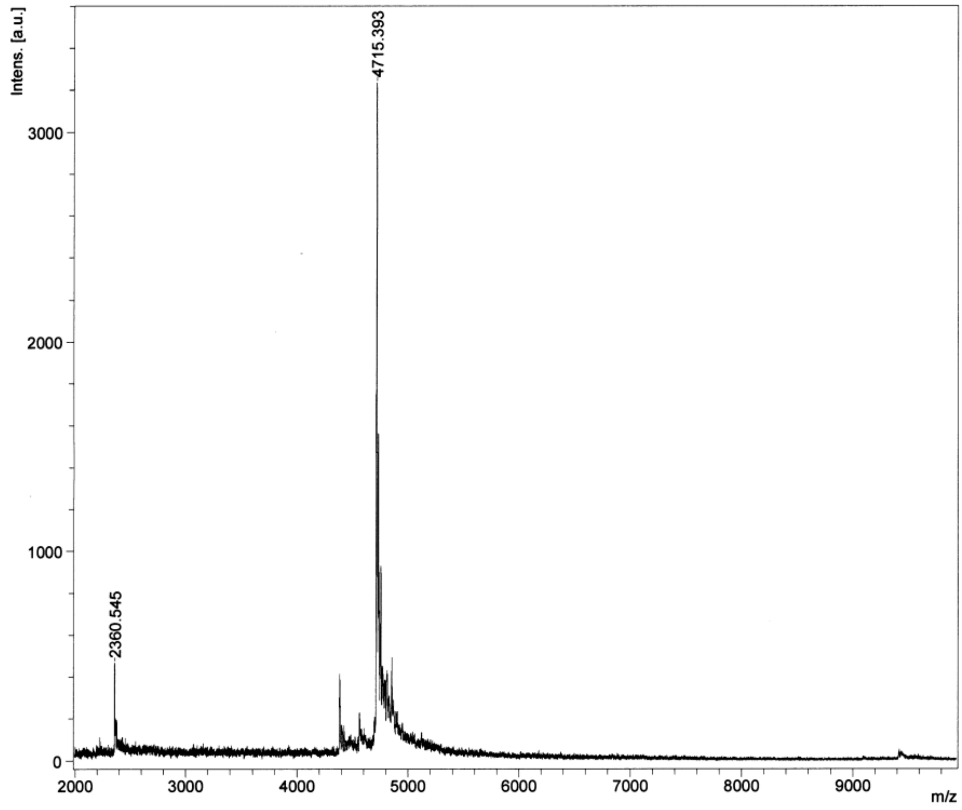

ODN4-^{N}Pu3

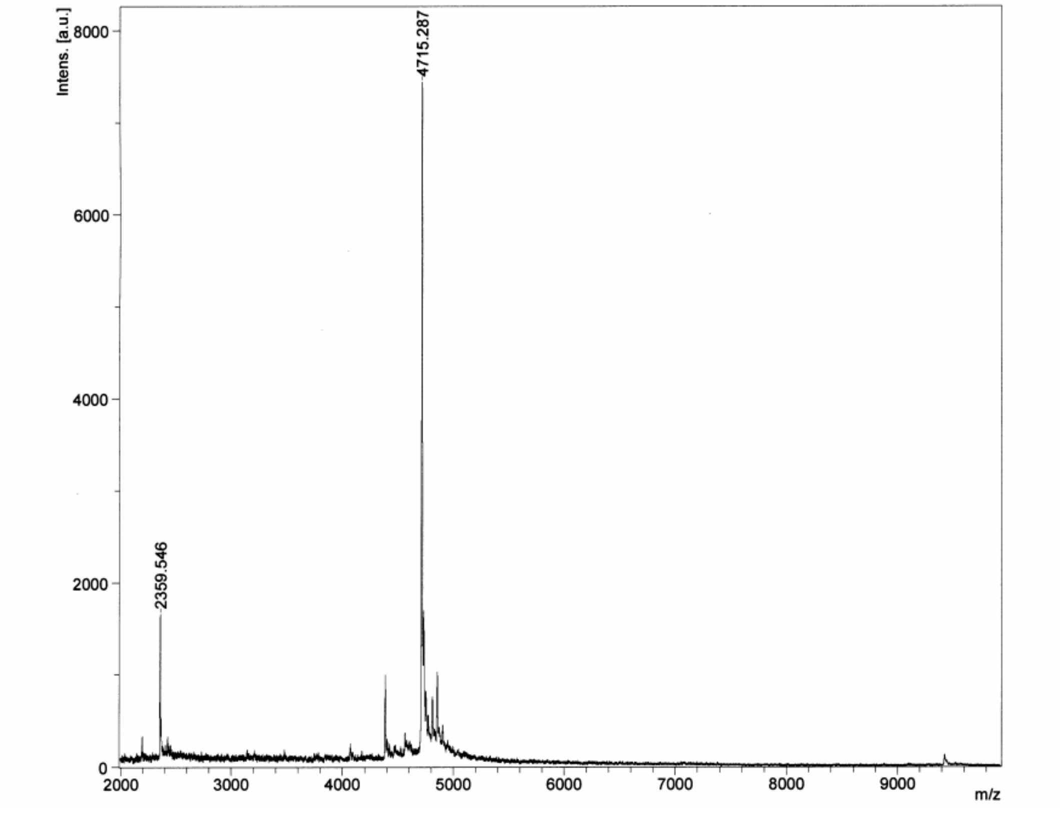

ODN4-^{O}Pu1

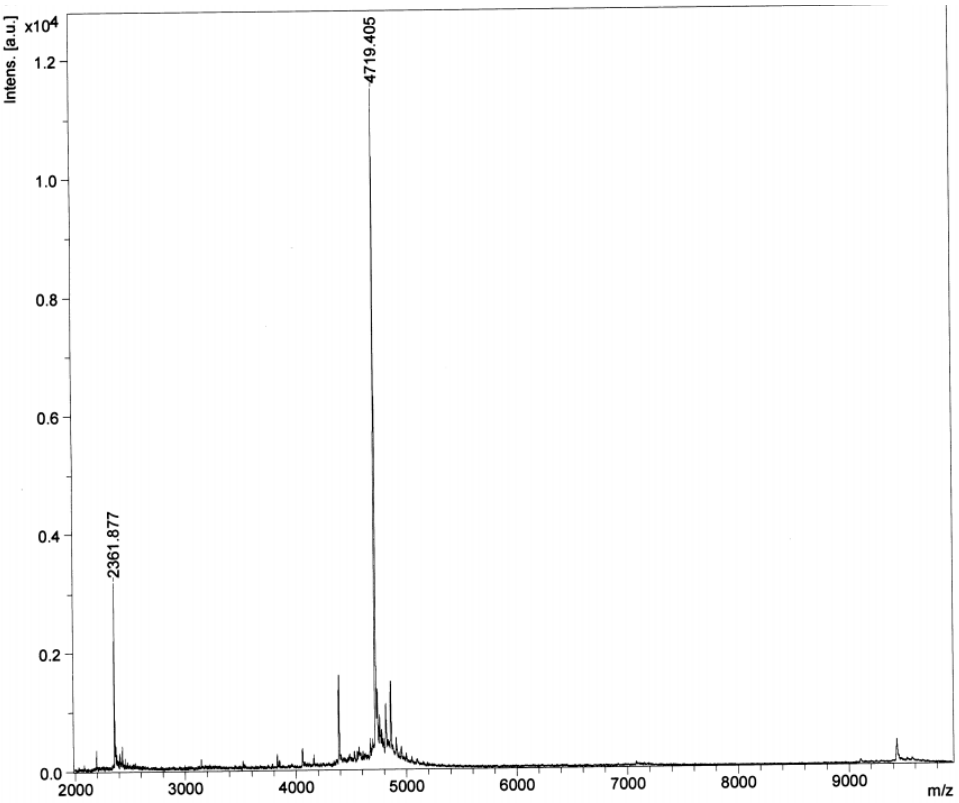

ODN4-^{O}Pu2

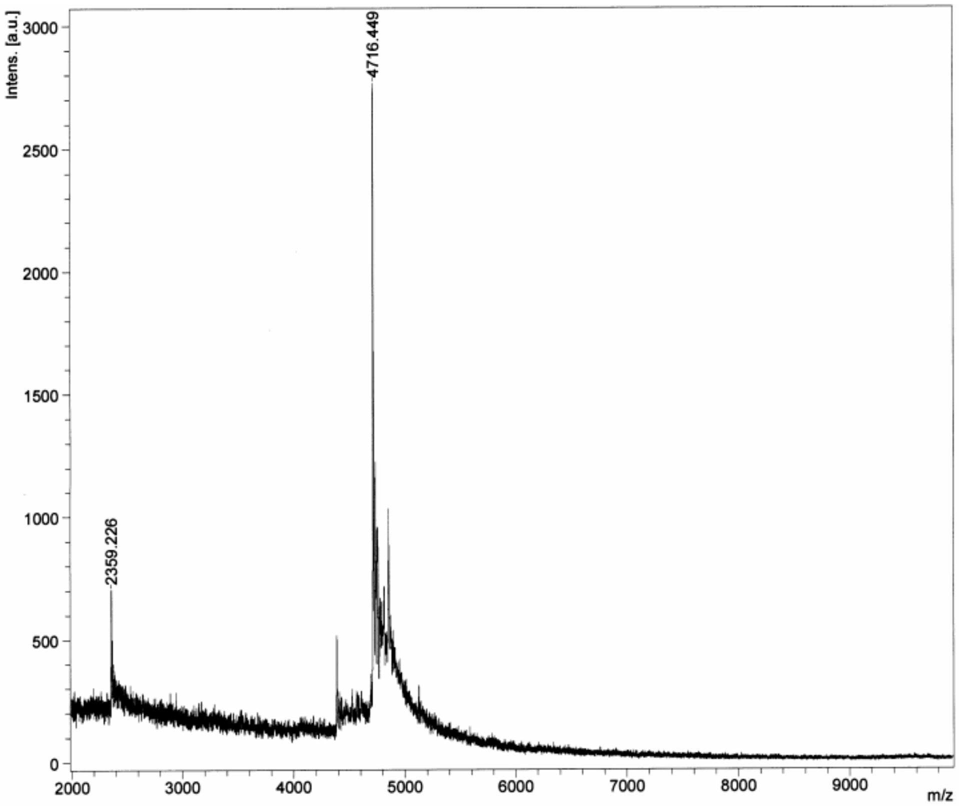

ODN4-^{O}Pu3

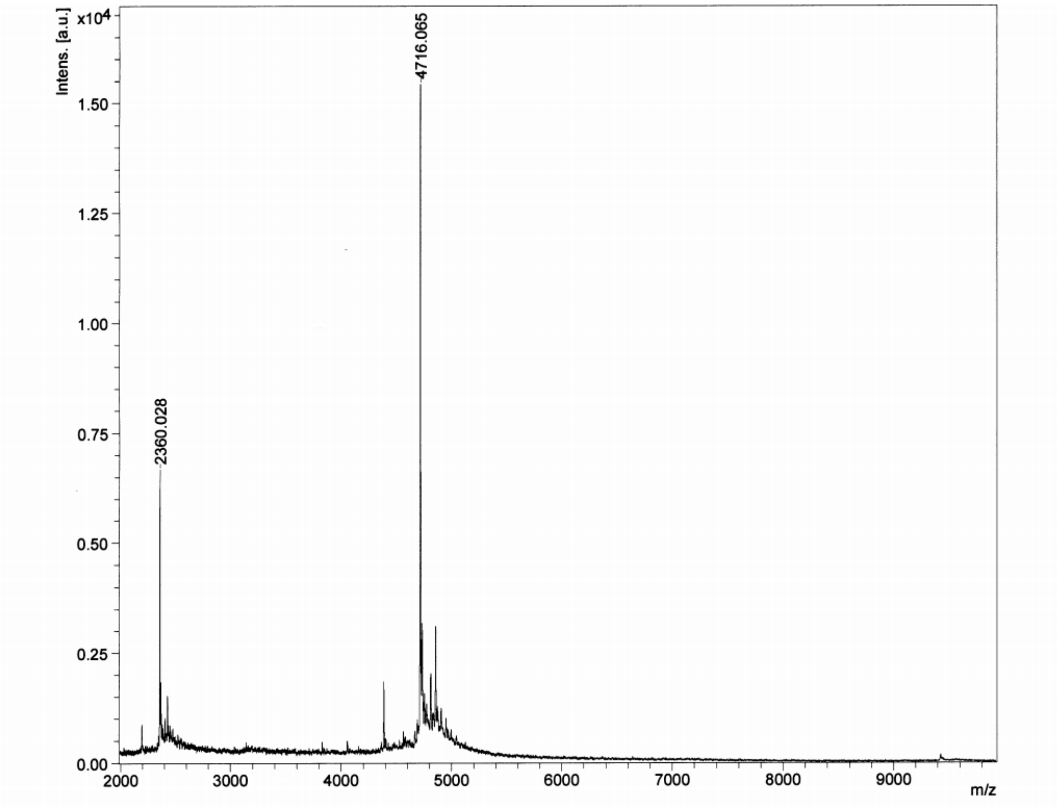
Intens. [a.u.]
x10^4
1.50
1.25
1.00
0.75
0.50
0.25
0.00
4716.065
2360.028
2000
3000
4000
5000
6000
7000
8000
9000
m/z
